# Halogenation as a tool to tune antimicrobial activity of peptoids

**DOI:** 10.1038/s41598-020-71771-8

**Published:** 2020-09-09

**Authors:** Natalia Molchanova, Josefine Eilsø Nielsen, Kristian B. Sørensen, Bala Krishna Prabhala, Paul Robert Hansen, Reidar Lund, Annelise E. Barron, Håvard Jenssen

**Affiliations:** 1 Department of Science and Environment, Roskilde University, 4000 Roskilde, Denmark; 2 Department of Chemistry, University of Oslo, 0315 Oslo, Norway; 3 The Molecular Foundry, Lawrence Berkeley National Laboratory, Berkeley, CA USA; 4 Department of Drug Design and Pharmacology, Faculty of Health and Medical Sciences, University of Copenhagen, 2100 Copenhagen, Denmark; 5 Institute of Physics, Chemistry and Pharmacy, Section for Pharmacy, University of Southern Denmark, Odense, Denmark; 6 Department of Bioengineering, School of Medicine and School of Engineering, Stanford University, Stanford, CA 94305 USA

**Keywords:** Antimicrobials, Antibiotics, Microbiology, Chemical biology, Peptides

## Abstract

Antimicrobial peptides have attracted considerable interest as potential new class of antibiotics against multi-drug resistant bacteria. However, their therapeutic potential is limited, in part due to susceptibility towards enzymatic degradation and low bioavailability. Peptoids (oligomers of *N*-substituted glycines) demonstrate proteolytic stability and better bioavailability than corresponding peptides while in many cases retaining antibacterial activity. In this study, we synthesized a library of 36 peptoids containing fluorine, chlorine, bromine and iodine atoms, which vary by length and level of halogen substitution in position 4 of the phenyl rings. As we observed a clear correlation between halogenation of an inactive model peptoid and its increased antimicrobial activity, we designed chlorinated and brominated analogues of a known peptoid and its shorter counterpart. Short brominated analogues displayed up to 32-fold increase of the activity against *S. aureus* and 16- to 64-fold against *E. coli* and *P. aeruginosa* alongside reduced cytotoxicity. The biological effect of halogens seems to be linked to the relative hydrophobicity and self-assembly properties of the compounds. By small angle X-ray scattering (SAXS) we have demontrated how the self-assembled structures are dependent on the size of the halogen, degree of substitution and length of the peptoid, and correlated these features to their activity.

## Introduction

The rapid emergence and widespread distribution of antibacterial resistance is recognized as one of the most serious global threats to human health^1–4^. Most antibiotics in clinical use are becoming sometimes ineffective in treating infections caused by either Gram-positive or Gram-negative superbugs^5,6^. Hence new antibiotics or alternative therapeutics are of a great importance for clinical treatments.

As an essential part of the innate immune system of nearly all living organisms, antimicrobial peptides (AMPs) are considered a promising therapeutic strategy in fighting bacterial infections^7^. With the rise of antimicrobial resistance and an urgent need for new antibiotics, AMPs have attracted more attention recently, resulting in 36 different AMPs undergoing clinical trials against various infectious diseases^8^. However, AMPs display a somewhat limited practical application due to rapid in vivo degradation and issues with systemic toxicity, as well as high production costs^9^. Poly-*N*-substituted glycines (peptoids), on the other hand, comprise a class of peptidomimetics where the side chains are attached to the backbone amide nitrogen rather than to the α-carbon^10^. Antimicrobial peptoids were first developed in 2003^11^. Over the past two decades, various antimicrobial peptoid designs have proven to efficiently retain their antimicrobial activity, without displaying any apparent stability downsides^12^.

Over 4,000 halogenated compounds isolated from natural sources constitute a diverse group of natural products that display a wide range of biological activities, including anticancer and antimicrobial properties^13,14^. However, until recently, only limited attention has been given to the identification of antimicrobial properties of peptides and peptidomimetics containing halogen atoms. So far, fluorination has received the most interest, though the studies tackling the link between fluorination and antimicrobial activity have led to somewhat inconclusive results. For example, introduction of hexafluoroleucine into magainin and buforin conferred enhanced antimicrobial activity and retained low hemolytic properties, while the presence of fluorine atoms and trifluoromethyl groups improved the potency of short cationic peptides^15–17^. By contrast, incorporation of hexafluoroleucine into protegrin analogs led to decreased potency, while lipopeptides with fluorinated tails demonstrated moderate antibacterial activity combined with pronounced hemolytic properties^18,19^. Bolt et al. have also demonstrated that substitution of fluorine in peptoids led to enhanced antimicrobial activity against both Gram-positive and Gram-negative bacteria with no to low toxicity towards mammalian cells^20^. Recently, Molchanova et.al. reported the link between introduction of fluorine atoms into the peptidomimetic sequences and increased antimicrobial activity against Gram-positive bacteria without significantly enhancing hemolytic potential^21,22^. Importantly, both vancomycin and salinosporamid A require the presence of one to two chlorine substituents to achieve their antimicrobial activity^23,24^. Jia et al. introduced fluorine, chlorine, bromine and iodine atoms into the honeybee peptide Jelleine-1 via halogen-substituted phenylalanine which led to improved protease stability; a fluorinated analogue showed similar antimicrobial activity to the parent peptide while chlorinated, brominated and iodinated analogues displayed two to eightfold increase in their activities in vitro^25^. Interestingly, the in vitro antimicrobial activity of the iodine analogue was the highest, while the chlorinated and brominated versions displayed a more potent efficacy in vivo. Both chlorinated and brominated variants of antibiotic NAI-107 have demonstrated higher antimicrobial activity, where the brominated one displayed slightly higher potency^26^. Peptoid 1 is an example of a well-studied promising antimicrobial peptoid with a wide spectrum of antimicrobial activity, however it exhibits relatively high cytotoxicity in vitro (although it has been tested and was reasonably well tolerated intraperitoneally in vivo against *S. aureus*)^27^. A recent attempt to enhance the antimicrobial potency of Peptoid 1 has also been reported, however the incorporation of fluorine or chlorine atoms via *N*spe units did not lead to any sufficient improvement in the antimicrobial profile^28^.

Introduction of halogens in the chemical structure of peptides or peptoids is known to generally increase the hydrophobicity of the molecules^29^. This may lead to conformational changes and self-assembly into supramolecular nanostructures, driven by increased hydrophobic interactions. Correlation between antimicrobial activity and self-assembly has been extensively discussed in the literature, where the impact on the antimicrobial properties and overall toxicity can trend in both directions^30,31^. While, for instance, Xu and co-workers found a link between increased antimicrobial activity and the self-assembly of defined supramolecular nanofibers, Chu-Kung and co-workers on the other hand found a clear tendency for their fatty acid conjugated peptides to show reduced antimicrobial activity^32,33^. Antimicrobial peptoids are significantly less prone to fold and form secondary structures, and to the authors’ knowledge, there have been no studies directly aimed to explore on the effect of self-assembly on the antimicrobial efficacy of peptoids.

In this study, we present the first structure–activity study of halogenated peptoids. The aim of the study is to investigate the link between the nature of the halogen, the amount of halogen substitution, their ability to self-assemble into nanostructures and their antimicrobial activity. Next, we proceeded to incorporate halogen atoms into the scaffold of the well-known Barron lab compound, Peptoid 1^34,35^, in an attempt to increase its antimicrobial activity, or to modulate its cytotoxicity.

## Results and discussion

### Probing the link between halogenation and antimicrobial activity

**Figure 1:**
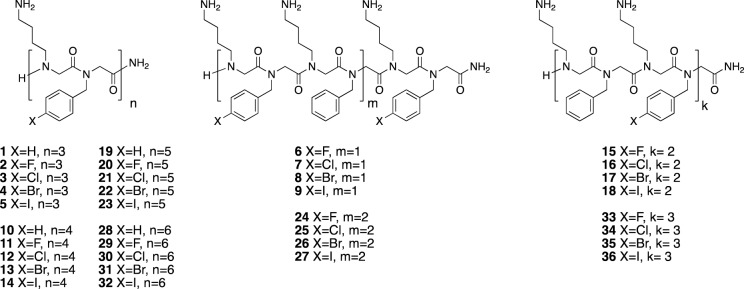
Structures of first-generation peptoids containing halogen atoms.

We synthesized 36 peptoids using a scaffold containing alternating *N*Lys and *N*pm units which vary by length (6-, 8-, 10-, 12-mers), and the level of halogen substitution (full or alternate). Halogen atoms (fluorine, chlorine, bromine or iodine) were introduced via phenyl rings in position 4 and synthesized using submonomer approach (Fig. 1).

All 36 peptoids were tested against seven bacterial strains: five Gram-positive (*Staphylococcus aureus* ATCC 25923 and ATCC 29213, methicillin-resistant *Staphylococcus aureus* USA 300, methicillin-resistant *Staphylococcus epidermidis* ET-024 and ATCC 51625) and two Gram-negative (*Escherichia coli* ATCC 25922, *Pseudomonas aeruginosa* PA01) (Table 1). The non-halogenated peptoids demonstrated no potency against the selected bacterial strains. Thus, compounds **1**, **10**, **19**, **28** (a 6-mer, 8-mer, 10-mer, and 12-mer respectively) represent the inactive controls.

**Table 1:** Minimal inhibitory concentrations (μg/mL) of the first generation peptoids containing halogen atoms.

Cmpd	RP-HPLC RT	SA^a^	SA^b^	MRSA^c^	MRSE^d^	MRSE^e^	EC^f^	PA^g^
**1**	11.49	> 512	> 512	> 512	512	512	> 512	> 512
**2**	12.08	> 512	> 512	512	128	512	512	> 512
**3**	13.06	64	64	16	16	8	512	512
**4**	13.32	16	16	16	4	4	64	> 512
**5**	13.67	8	4	4	4	2	64	> 512
**6**	12.00	> 512	> 512	> 512	256	512	> 512	> 512
**7**	12.53	256	256	128	64	32	256	512
**8**	12.72	128	64	64	32	16	256	256
**9**	12.97	64	16	16	16	4	256	256
**10**	11.69	> 512	> 512	> 512	256	256	256	> 512
**11**	12.30	256	256	256	64	32	128	> 512
**12**	13.32	4	4–8	2	2	1	64	> 512
**13**	13.60	4	8	2	2	4	64	> 512
**14**	13.94	8	16	4	2	4	64	> 512
**15**	12.01	> 512	> 512	> 512	128	128	512	512
**16**	12.55	16	64	16	8	8	256	512
**17**	12.67	16	32	32	4	4	256	512
**18**	12.94	32	32	16	8	4	256	> 512
**19**	11.84	> 512	> 512	> 512	128	128	256	512
**20**	12.41	64	64	32	4	8	128	256
**21**	12.96	2	2	2	1	2	64	> 512
**22**	13.75	2	2	4	1	1	64	> 512
**23**	14.09	32	32	16	4	8	> 512	> 512
**24**	12.20	512	512	256	4	8	256	512
**25**	12.85	8	8	16	2	1	256	512
**26**	13.02	4	4	8	1	1	256	512
**27**	13.25	4	4	4	2	1	128	> 512
**28**	11.93	> 512	512	512	64	32	128	128
**29**	12.52	32	32	16	4–8	4	256	64
**30**	13.57	2	2	2	2	1	64	> 512
**31**	13.85	4	8	2	4	4	64	256
**32**	14.16	64	128	32	32	16	> 512	> 512
**33**	12.25	256	256	128	16	4	256	256
**34**	12.43	16	8	8	4	1	256	> 512
**35**	12.95	8	8	8	8	1	256	> 512
**36**	13.16	8	16	4	4	1	256	> 512

^a^*S. aureus* ATCC 25923.

^b^*S. aureus* ATCC 29213.

^c^Methicillin-resistant *S. aureus* USA 300.

^d^Methicillin-resistant *S. epidermidis* ET-024.

^e^Methicillin-resistant *S. epidermidis* ATCC 51625.

^f^*E. coli* ATCC 25922.

^g^*P. aeruginosa* PA01; RP-HPLC RT -reversed-phase HPLC retention time.

The 36 compounds are divided in four sets according to their length. Each set contains a non-substituted control, four fully substituted peptoids with fluorine, chlorine, bromine or iodine, and four “half substituted” peptoids where every second phenyl ring is substituted with a halogen atom.

Halogenation had no effect on the activity of the peptoids against either *E. coli* and *P. aeruginosa* (Table 1). However, a clear correlation was observed between antimicrobial activity against Gram-positive strains, the level of substitution, and the nature of a halogen, among all sets. The fully halogenated peptoids demonstrated drastically enhanced activity against wild type and resistant strains of both *S. aureus* and *S. epidermidis*. Interestingly, for 6- (**2**–**5**) and 8-mers (**11**–**14**) the activity rose from fluorine to iodine, where the latter was most potent. For the 6-mers, addition of just three iodine atoms led to up to > 64-fold increase against *S. aureus* (MIC for **1** =  > 512 μg/mL; for **5** = 8 μg/mL) and 256-fold increase against MRSE (MIC for **1** = 512 μg/mL; for **5** = 2 μg/mL), while 8-mer **14** exhibited the same activity against *S. aureus*, and 128-fold increase against MRSE (MIC for **10** = 256 μg/mL; for **14** = 2 μg/mL). However, moving to fully substituted 10-mer (**20**–**23**) and 12-mer (**29**–**32**) sets, the antimicrobial trend is lost as compounds bearing iodine atoms displayed lower potency against both *S. aureus* and multidrug resistant *S. epidermidis* compared to their chlorinated and brominated analogues. Bromination of the 10-mer led to > 256-fold and 128-fold increase of activity against *S. aureus* and MRSE compared to the unsubstituted control peptoid (*S. aureus*: MIC for **19** =  > 512 μg/mL; for **22** = 2 μg/mL; MRSE: MIC for **19** = 128 μg/mL; for **22** = 1 μg/mL), while a brominated 12-mer analogue displayed similar or lower activity against both *S. aureus* and *S. epidermidis*.

The “half-substituted” sets fell under a similar trend, though generally displaying similar or lower potency. Interestingly, the half-substituted peptoids bearing bromine exhibited comparable activity to their fully substituted analogues. For example, compound **26** showed MICs between 1–8 μg/mL versus 1–4 μg/mL for the fully substituted analogue **22**.

As expected, the peptoids’ hydrophobicity increased with the addition of halogen atoms, where fluorine displayed a less pronounced effect while incorporation of iodine led to noticeably higher hydrophobicity profiles. In parallel, the peptoids’ antimicrobial activity fell into a well-established correlation, where increase in hydrophobicity led to an increased antimicrobial activity. However, when a certain hydrophobicity threshold was met (e.g. for compounds **23** and **32**), the activity was lost, the phenomena that has been previously observed in other peptide/peptoid studies^36,37^. All in all, we could see that introduction of halogen atoms led to an increase of hydrophobicity that accompanies an increase of antimicrobial activity against Gram-positive bacteria. However, when a certain level of hydrophobicity was met, the activity of peptoids started decreasing.

### Studying the effects of halogen substitutions on peptoid self-assembly in solution

To further understand the impacts of variation in length, size of halogen groups and degree of substitution, the nanostructures of these compounds were studied in detail using Small Angle X-ray Scattering (SAXS).

**Figure 2:**
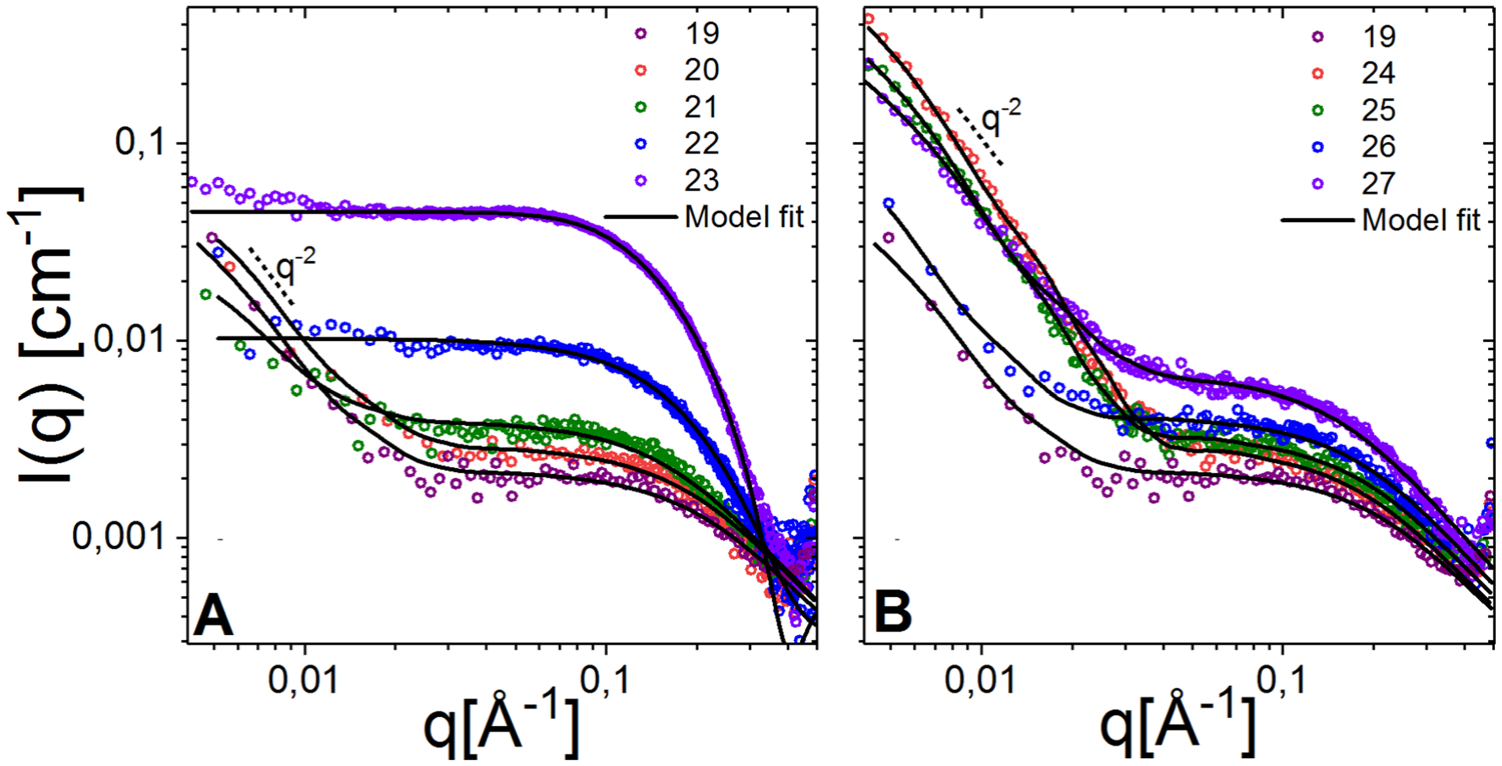
SAXS data showing the scattered intensity plotted towards the modulus of the scattering vector, q for 10-mer peptoids at 5 mg/mL obtained at BM29, ESRF at 37 °C. (**A**) displays the fully halogenated and (**B**) includes half-halogenated peptoids. The non-halogenated peptoid **19** is included in both graphs as a reference. Results indicate predominantly free unstructured peptoid chains with a small fraction of sheet-like filaments for compounds **19–21** and **24–27**, and defined bundles for compounds **22** and **23**, seen as an increase in the scattering intensity and a change in shape of the curve.

SAXS allows for the determination of whether these peptoids self-assemble into nanostructures or instead exist as single molecules in aqueous solution^38–41^. Furthermore, through detailed theoretical modelling, the techniques allow for an accurate estimation of molecular weight, shape and the overall physical structures of peptoid assemblies. The results revealed that the observed structures depend on the length and hydrophobicity of the various peptoids; and self-assembly into defined nanostructures was observed for a few of them. Scattering intensity is plotted as a function of the modulus of the scattering vector, q = 4πsin(θ/2)/λ, where λ is the wavelength of the X-rays and θ is the scattering angle (Fig. 2). It should be noted that 1/q has the dimension of length and the quantity represents a sort of ‘measuring stick’; at low q, large structures are probed, while at high q, SAXS is sensitive to more local structures.

For the 10-mers (compounds **19**–**27**), the scattering curve for the fully brominated **22** and fully iodinated **23** peptoids exhibited significantly higher intensity and a different shape as compared to the rest of the 10-mers (Fig. 2A,B). The latter exhibited typical polymer-like scattering pattern for random (Gaussian) chains although an upturn at low q revealed a small fraction of larger structures or aggregates. The upturn follows a power law of ~ q^−2^ indicating plate-like fibers, and no larger aggregates that would typically follow the power law of ~ q^−4^^42^. Through fit analysis of these data using a model with a combination of free chains and rectangular fibers, we obtained a radius of gyration (Rg) of the free chains of 7–9 Å, and a small mole fraction of only 0.001–0.0005% fiber-like sheets (see supporting information Table S1 for full list of fit parameters).

The scattering for the fully brominated **22** and all iodinated **23** did not exhibit the same upturn at low q and overall shape at intermediate and high q could therefore not be explained with the fit model described above. Instead, the scattering intensity exhibited a flatter q-dependence at low q indicating discrete, smaller nanostructures. The data from both compounds could be analyzed using a bundle model where cylinders representing folded/helical units are assembled into trimeric/tetrameric bundles^39^. The scattering of a concentration range from 5–0.6 mg/mL of both systems was analyzed simultaneously and the obtained fit parameters are listed in Table S1. The fit analysis also revealed the critical aggregation concentration (CAC) for the self-assembled structures and indicated a CAC value of 2.3 mg/mL and 0.4 mg/mL for the fully brominated **22** and iodinated **23**, respectively (see supporting information Figure S1). The lower CAC value of **23** is a result of the high hydrophobicity as reported in Table 1. The increased hydrophobicity might provide an explanation of the observed loss of antimicrobial activity for the fully iodinated 10- and 12-mer (c.f. Table 1). These results suggest that a clear correlation between self-assembly and activity as seen by Chu-Kung and co-workers in the past^32^, cannot be drawn for these compounds as the estimated lowered CAC from SAXS is higher than the MIC values.

**Figure 3:**
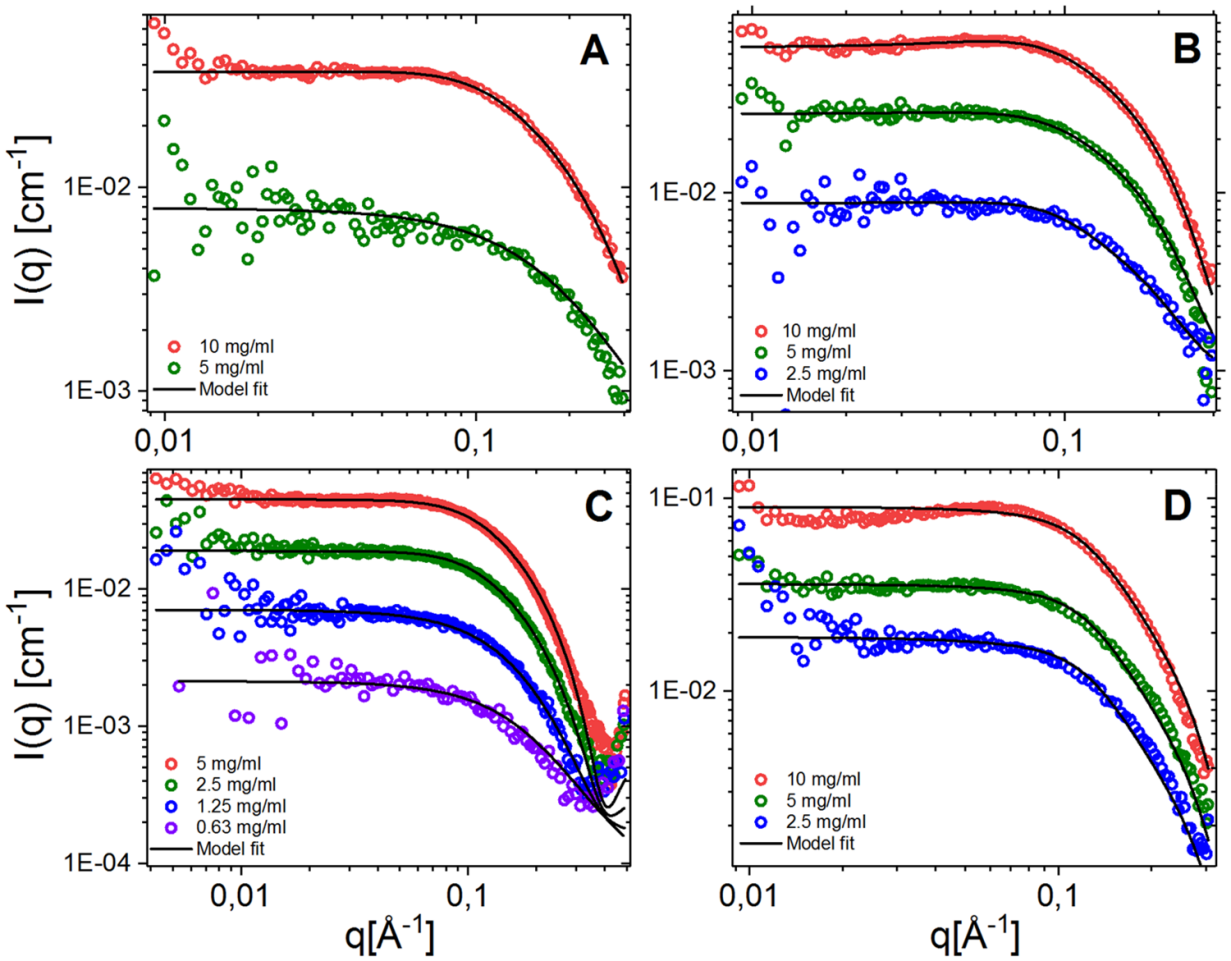
Full SAXS results for the fully iodinated peptoids measured with Bruker NANOSTAR instrumentation (except the 10-mer **23** measured at ESRF) in the indicated concentrations together with model fits (fitted using the bundle model as described in the supplementary information). Panels **A**, **B**, **C** and **D** shows the 6-mer (**5**), 8-mer (**14**), 10-mer (**23**) and 12-mer (**32**) respectively.

To further investigate if the self-assembly properties of the iodinated peptoids, we proceeded with the 6-, 8- and 12-mer of the fully iodinated peptoids (**5**, **14**, **23**, **32**). The results are shown in Fig. 3 (see Table S2 for fit parameters). From the fit analysis, we found a CAC of 2.8 mg/mL for the fully iodinated 6-mer compound **5**, 1.4 mg/mL for the 8-mer **14** and 0.4 mg/mL for the 12-mer **32**.

These results show that even at very short lengths, these peptoids self-assemble in the probed concentration range due to their high hydrophobicity, which is evident from the retention times in Table 1. The detected CAC is highly correlated with the length of the peptoid. However, as seen from the MIC values these shorter peptoids are highly active compared to the 10-mer and 12-mer, indicating a potential threshold of hydrophobicity. Furthermore, the detected CAC is still highly correlated with the length of the peptoid, but a link between CAC and MIC could not be drawn. This is in contrast with the fatty acid conjugated peptides studied by Chu-Kung and co-workers, who found a correlation^32^. The reduction of activity seen for the 10-mer and 12-mer is therefore likely related to the increased hydrophobicity, indicating that there is a threshold to stay within than the self-assembly properties themselves. However, further studies into the consequences of hydrophobicity and self-assembly with the activity and toxicity for peptoids are needed to fully explain the observed trends.

### Halogenation as a tool to improve antimicrobial activity

**Figure 4:**
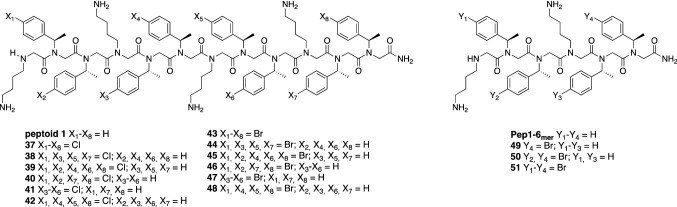
Chlorinated and brominated variants of peptoid 1 and shortened brominated peptoid 1 analogues.

In order to investigate whether halogens can be used as a tool to improve the antimicrobial potency of a known peptoid, we chose the well-studied Peptoid 1 and synthesized two small libraries of chlorinated and brominated analogues as these halogens demonstrated overall higher potency compared to the fluorinated ones, and iodine variants raised the concern of aggregation and loss of activity. Both halogen atoms were introduced via *N*spe units in position 4 on the phenyl rings. The level of substitution varied between full substitution (all phenyl rings bear a halogen atom) and “half” substitution (every other phenyl ring bears a halogen atom). This strategy yielded six chloro- and six bromo- modified versions of Peptoid 1 (Fig. 4).

The library of 12 compounds (**37**–**48**) was tested against the same panel of bacterial strains as the first generation of peptoids. However, none of the modifications led to increased potency, while most caused a loss of activity (Table 2). Judging from long HPLC retention times, this could be explained by the fact that the critical hydrophobicity level was reached, which causes aggregation and loss of activity similar to what we have seen for compounds **23** and **32**, as the highest activity was observed for the compounds with lower retention times. Interestingly, compounds **38**, **40**–**42** have the same number of chlorine atoms, but their hydrophobicity differ. For the brominated derivatives **44**, **46**–**48**, this effect was even more pronounced. It shows that even distribution of halogen atoms across the peptoid chain led to higher hydrophobicity compared to positioning chlorine or bromine atoms at the ends or the middle of the peptoid sequence.

**Table 2:** MICs (μg/mL) of the chlorinated and brominated analogues of Peptoid 1.

Cmpd	RP-HPLC RT	SA^a^	MRSA^b^	MRSE^c^	MRSE^d^	EC^e^	PA^f^
Peptoid 1	15.46	8	8	4	8	8	16
37	18.25	> 64	64	64	> 64	> 64	> 64
38	16.80	32	16	32	16	64	> 64
39	16.93	16	16	16	16	64	> 64
40	16.09	8	8	8	8	> 64	16
41	16.20	8	8	8	8	64	16
42	16.86	16	16	16	16–32	64	> 64
43	18.88	> 64	> 64	> 64	> 64	> 64	> 64
44	17.03	64	32	32	32	64	> 64
45	17.21	32	32	32	64	> 64	> 64
46	16.23	16	8	8	8	> 64	16
47	15.68	8	8	4	8	64	16
48	17.14	64	32	32	32	64	> 64

^a^*S. aureus* ATCC 25923.

^b^Methicillin-resistant *S. aureus* USA 300.

^c^Methicillin-resistant *S. epidermidis* ET-024.

^d^Methicillin-resistant *S. epidermidis* ATCC 51625.

^e^*E. coli* ATCC 25922.

^f^*P. aeruginosa* PA01 H103; RP-HPLC RT Reversed-Phase HPLC retention time.

### Short brominated Peptoid 1 analogues

As seen from the increased values of retention times, we have hit the hydrophobicity ceiling during the introduction of halogens into the sequence of Peptoid 1 (Table 2), hence we decided to shorten the length of Peptoid 1 from twelve to six residues, cutting its length in half, and synthesized a small library of four analogues with different levels of halogenation (Fig. 4). As seen from Tables 1 and 2, introduction of fluorine didn’t result in the desired increase of activity, while bromination had a more pronounced effect on the hydrophobicity than chlorination and similar to one displayed by the iodine-containing peptoids. Hence, three brominated short Peptoid 1 analogues (**49**–**51**) and one non-halogenated peptoid **Pep1-6**_**mer**_ were synthesized and tested against the same bacterial strains as the previous peptoids. Data are shown in Table 3 where the new data set is compared with the original Peptoid 1 data^27^.

**Table 3:** MICs (μg/mL) and IC_50_ (μg/mL) towards HaCaT cell line of short brominated Peptoid 1 analogues.

cmpd	RP-HPLC RT	SA^a^	MRSA^b^	MRSE^c^	MRSE^d^	EC^e^	PA^f^	IC_50_ (μg/mL)
Peptoid 1	3.68	8	8	4	8	8	16	35.0 (30.9 to 39.9)
Pep1-6_mer_	3.08	256	256	64	64	1,024	256	632.4 (554.6 to 731.5)
49	3.59	64	32	16	8	> 64	64	250 (222.2 to 280.5)
50	3.84	8	8	4	2	32	16	146.9 (131.6 to 165.3)
51	4.02	8	8	4	8	16	16	92.6 (75.7 to 112.3)

^a^*S. aureus* ATCC 25923.

^b^Methicillin-resistant *S. aureus* USA 300.

^c^Methicillin-resistant *S. epidermidis* ET-024.

^d^Methicillin-resistant *S. epidermidis* ATCC 51625.

^e^*E. coli* ATCC 25922.

^f^*P. aeruginosa* PA01 H103; RP-HPLC RT Reversed-Phase HPLC retention time.

Compound **49** has only one terminal bromine, **50** has two bromine atoms and for **51**—all four phenyl rings are substituted with a bromine atom. The addition of just two bromine atoms was enough to improve the activity of a non-active short analogue, the **Pep1-6**_**mer**,_ 16–32-fold against both *S. aureus* and *S. epidermidis*. Incorporation of two extra bromines yielding four in total failed to significantly affect the activity. Addition of one terminal halogen atom, on the other hand, was already enough to see a several-fold increase in antimicrobial activity. Despite the previous data, where introduction of halogens did not improve the activity against Gram-negative bacteria, in this case addition of two bromines was enough to reach sufficient hydrophobicity in order to obtain an MIC against *P. aeruginosa* that is close to the original Peptoid 1 and reach 32-fold increase of activity against *E. coli* compared to the **Pep1-6**_**mer**_.

**Figure 5:**
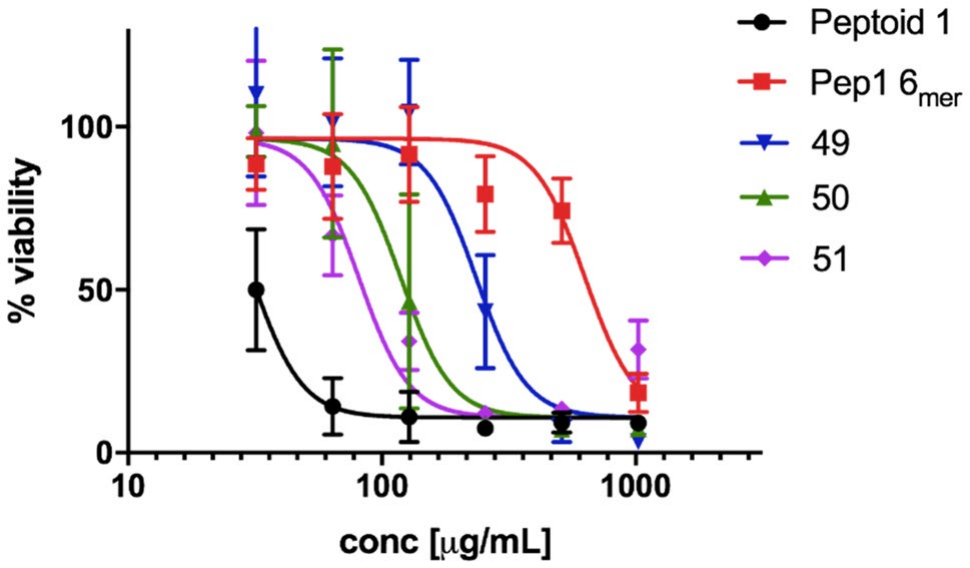
IC_50_ curves of the selected peptoids toward HaCaT cell line.

As we have shown that the incorporation of halogens can improve the activity of inactive peptoids, we decided to examine their cytotoxicity profiles. Peptoid 1, Pep1-6_mer_ and the three brominated analogues were tested towards a HaCaT cell line for 1 h. The results were obtained using MTS/PMS assay (Fig. 5, Table 3).

We have observed a clear trend between the number of halogen atoms and corresponding increased cytotoxicity, however while compound **50** demonstrated the same antimicrobial activity profile to Peptoid 1 it was less cytotoxic with IC_50_ 146.9 μg/mL versus only 35.0 μg/mL for the latter. All in all, initial results indicate reduced cytotoxicity of the brominated analogues when compared to Peptoid 1, though compounds **50** and **51** displayed similar activity and hydrophobicity profiles.

## Conclusions

In this study, we have investigated the effect of fluorine, chlorine, bromine and iodine on the antimicrobial activity of peptoids. First, using an inactive model (*N*lys-*N*pm)_n_ peptoid scaffold, we have identified that incorporation of chlorine or bromine led to an improvement of antimicrobial activity against Gram-positive bacteria, while fluorination did not display any pronounced effect. Introduction of iodine in 6- and 8-mers analogues dramatically increased the activity, but led to loss of activity due to aggregation in 10- and 12-mers. Afterwards, we attempted to improve the antibacterial potency of Peptoid 1 by incorporating chlorine or bromine atoms via *N*spe units, which led to overall loss of activity. We interpret this oberservation as sign of the hydrophobicity limits being reached. However, bromination of a shorter inactive 6-mer analogue of Peptoid 1 resulted in the same activity as the 12-mer Peptoid 1 against some bacteria, while noticeably improving its cytotoxic profile.

Therefore, here we have shown that halogenation, and particularly bromination, can be used to readily modify and alter the physicochemical and antibacterial properties of peptoids but the effect strongly depends on the choice of the halogen. In addition, the effect is quite sequence- and length- specific, and inclusion of halogens can also lower antimicrobial activity.

## Methods

### General information

Solvents, amines, and resins were purchased from Iris Biotech, Sigma‐Aldrich, and Merck and used without further purification. For compounds 1–48: purity was determined as described previously^49^, by analytical HPLC using a Waters 717 plus Autosampler, In-line Degasser AF, 600 Controller, and 2,996 Photodiode Array Detector; the column used was a Waters Symmetry C18, 5 µm, 4.6 mm × 250 mm. An aqueous acetonitrile (MeCN) gradient with 0.1% trifluoroacetic acid (TFA) added (eluent A: 5:95 MeCN‐H_2_O + 0.1% TFA, eluent B: 95:5 MeCN‐H_2_O + 0.1% TFA) was employed using water filtered through a 0.22‐μm Millipore membrane. All tested compounds had a purity of at least 95%. Preparative HPLC was performed by using a Waters XSelect Peptide CSH C18 OBDTM, 5 µm, 19 mm × 250 mm column and the same eluents as for analytical HPLC. High‐resolution mass spectrometry (HRMS) spectra were obtained by using a Waters QTOF premier mass spectrometer equipped with an electrospray ionization source and a Quadruple and time of flight MS detector. For compounds 49–51, pep1-6mer and peptoid 1: Product purity was determined by means of analytical UPLC/MS using a Water Acquity UPLC system, equipped with an Acquity Diode Array UV detector and a Waters SQD2 mass spectrometer. As stationary phase, a Waters Acquity UPLC Peptide BEH C18 Column (300 Å pore size, 1.7 µm particle size, 2.1 mm × 100 mm) with an Acquity UPLC BEH C18 VanGuard pre‐column (1.7 μm, 2.1 mm × 5 mm) was employed. Purification by means of preparative HPLC was carried out using a Waters Prep150LC system, equipped with a Waters 2,489 UV/Visable detector and a Waters Fraction Collector III collector. As stationary phase, a Waters XBridge BEH300 Prep C18 column (5 μm particle size, 19 mm × 100 mm) with a Waters XBridge Peptide BEH300 C18 guard column (5 μm particle size, 19 mm × 10 mm) was employed.

### General synthesis of peptoids

All peptoids have been synthesized manually on Rink amide resin (Novabiochem, 0.65 mmol/g) according to the submonomer method^10^. After synthesis, oligomers were cleaved and deprotected in trifluoroacetic acid (TFA)/triisopropylsilane/water (95:2.5:2.5 by vol.) for 30 min.

### Determination of minimal inhibitory concentration

Bacterial growth inhibition was determined by using broth microdilution according to the Clinical Laboratory Standards Institute^43^. The antibacterial activity of peptoids was tested against *S. aureus* ATCC 25923, ATCC29213, and methicillin resistant *S. aureus* USA 300, methicillin resistant *S. epidermidis* ATCC 51625, a biofilm producing methicillin resistant *S. epidermidis* ET-024^44^, *P. aeruginosa* Pa01 (H103), and *E. coli* ATCC 25922. Bacteria, grown on agar plates for 18 h at 37 °C, were diluted to ∼1 × 10^8^ CFU/mL in Mueller‐Hinton Broth II (MHB II). Twofold serial dilutions of peptoids in MHB II were inoculated with bacteria to achieve a final concentration of 5 × 10^5^ CFU/mL in polypropylene 96 U‐well microtiter plates (Corning 3,897; ThermoFisher Scientific, Roskilde), followed by incubation at 37 °C in ambient air for 18 h. The MIC values were determined as the lowest concentration showing no visible bacterial growth. Experiments were performed twice (in technical triplicates) on 2 different days.

### Small angle X-ray scattering

SAXS experiments of the 10-mer peptoids were performed at the automated BM29 bioSAXS beamline at the European Synchrotron Radiation Facility (ESRF) in Grenoble, France^45^. The data was obtained using an energy of 12.5 keV and a detector distance of 2.87 m, covering a q range ($q=4\pi \mathrm{sin}(\theta /2)/\lambda$), where $\theta$ is the scattering angle and $\lambda$ is the X-ray wavelength) of about 0.0047 to 0.5 Å^−1^. The data set was calibrated to an absolute intensity scale using water as a primary standard. 40 µL samples were run through a capillary using the flow mode of the automated sample changer^46^. SAXS data was collected in ten successive frames of 0.5 s each to monitor radiation damage and the data reduction was done using the standard tool at BM29^47^.

The SAXS experiments on the fully iodinated 6-, 8-, and 12-mers to determine the CAC of the compounds were performed using a Bruker NANOSTAR equipped with a microfocus X-ray source (IμS Cu, Incoatec, Germany) and a VÅNTEC-2000 detector. Raw scattering data was calibrated to absolute intensity scale using water as a primary standard and radially averaged in order to obtain the 1D scattered intensity profile as a function of the scattering vector, with a wavelength of 1.54 Å. Two concentrations of compound **23** and **19** was also run on the NANOSTAR to verify that the results were comparable with the results from synchrotron SAXS at ESRF.

The modelling fit analysis of the scattering data is explained in detail in the supplementary information.

### Cell culturing

An immortalized human keratinocytes (HaCaT) cell line (Gift from David G. Naym at Bispebjerg Hospital) were cultured to ∼90% confluence after 21–25 h of growth at 5% CO_2_ and 37 °C as previously^48^. In brief, the cells were cultured in Dulbecco’s Modified Eagle’s Medium supplemented with 10% (v/v) fetal bovine serum, penicillin (100 IU/mL), and streptomycin (100 μg/mL), purchased from Sigma-Aldrich (St. Louis, MO, United States).

### Cell viability assay

Cell viability assessment was performed on cell monolayers grown to ∼90% confluence in 96-well plates Corning Costar (Sigma-Aldrich, Brøndby, Denmark) by using the MTS/PMS assay as previously described^48^. Briefly, the adhered cells were washed with 37 °C PBS solution (ThermoFisher Scientific, Roskilde) and exposed for 1 h at 37 °C to 100 μL of peptoids dissolved in the medium also used for culturing of the cell line (at concentrations in the range 0–1,000 μg/mL). The precise exposure-time were selected to enable comparison with related peptoid compounds^21,35^. Then the cells were washed twice with 37 °C PBS and then 100 μL of an MTS/PMS solution in media, consisting of 240 μg/mL MTS (Promega, Madison, WI, United States) and 2.4 mg/mL PMS (Promega, Madison, WI, United States), were added to the cells, which then were incubated for 1 h at 37 °C protected from light. A plate reader (SpectraMax i3X; Molecular devices, San Jose, CA) was used to measure the absorbance at 492 nm. The relative viability was calculated by using 0.2% (w/v) sodium dodecyl sulfate (SDS) as the positive control, while cells exposed to medium without test compound were used as the negative control. Data were obtained in three independent biological replicates performed on separate passages of cells and on separate days with a total number of six replicates.

## Supplementary information

Supplementary information

## References

[1] Bassetti M, Merelli M, Temperoni C, Astilean A (2013). New antibiotics for bad bugs: Where are we?. Ann. Clin. Microbiol. Antimicrob..

[2] Mulani MS, Kamble EE, Kumkar SN, Tawre MS, Pardesi KR (2019). Emerging strategies to combat ESKAPE pathogens in the era of antimicrobial resistance: A review. Front. Microbiol..

[3] Chen CH, Lu TK (2020). Development and challenges of antimicrobial peptides for therapeutic applications. Antibiotics (Basel)..

[4] WHO. "Critically Important Antimicrobials for Human Medicine." 6th.

[5] Dickey SW, Cheung GYC, Otto M (2017). Different drugs for bad bugs: Antivirulence strategies in the age of antibiotic resistance. Nat. Rev. Drug Discov..

[6] Friedman ND, Temkin E, Carmeli Y (2016). The negative impact of antibiotic resistance. Clin. Microbiol. Infect..

[7] Zasloff M (2002). Antimicrobial peptides of multicellular organisms. Nature.

[8] Koo HB, Seo J (2019). Antimicrobial peptides under clinical investigation. Pept. Sci..

[9] Mahlapuu M, Hakansson J, Ringstad L, Bjorn C (2016). Antimicrobial peptides: An emerging category of therapeutic agents. Front. Cell Infect. Microbiol..

[10] Zuckermann RN, Kerr JM, Kent SBH, Moos WH (1992). Efficient method for the preparation of peptoids [oligo(N-substituted glycines)] by submonomer solid-phase synthesis. J. Am. Chem. Soc..

[11] Patch JA, Barron AE (2003). Helical peptoid mimics of magainin-2 amide. J. Am. Chem. Soc..

[12] Molchanova N, Hansen PR, Franzyk H (2017). Advances in development of antimicrobial peptidomimetics as potential drugs. Molecules.

[13] Neumann CS, Fujimori DG, Walsh CT (2008). Halogenation strategies in natural product biosynthesis. Chem. Biol..

[14] Gribble GW (2004). Natural organohalogens: a new frontier for medicinal agents?. J Chem. Educ..

[15] Gimenez D (2006). The introduction of fluorine atoms or trifluoromethyl groups in short cationic peptides enhances their antimicrobial activity. Bioorg. Med. Chem..

[16] Meng H, Kumar K (2007). Antimicrobial activity and protease stability of peptides containing fluorinated amino acids. J. Am. Chem. Soc..

[17] Paulsen MH (2018). An amphipathic cyclic tetrapeptide scaffold containing halogenated beta(2,2)-amino acids with activity against multiresistant bacteria. J. Pept. Sci..

[18] Gottler LM, de la Salud BR, Shelburne CE, Ramamoorthy A, Marsh EN (2008). Using fluorous amino acids to probe the effects of changing hydrophobicity on the physical and biological properties of the beta-hairpin antimicrobial peptide protegrin-1. Biochemistry.

[19] Findlay B, Zhanel GG, Schweizer F (2012). Investigating the antimicrobial peptide 'window of activity' using cationic lipopeptides with hydrocarbon and fluorinated tails. Int. J. Antimicrob. Agents.

[20] Bolt HL (2017). Exploring the links between peptoid antibacterial activity and toxicity. Med. Chem. Commun..

[21] Molchanova N, Hansen PR, Damborg P, Nielsen HM, Franzyk H (2017). Lysine-based alpha-peptide/beta-peptoid peptidomimetics: Influence of hydrophobicity, fluorination, and distribution of cationic charge on antimicrobial activity and cytotoxicity. ChemMedChem.

[22] Molchanova N, Hansen PR, Damborg P, Franzyk H (2018). Fluorinated antimicrobial lysine-based peptidomimetics with activity against methicillin-resistant *Staphylococcus pseudintermedius*. J. Pept. Sci..

[23] Harris CM, Kannan R, Kopecka H, Harris TM (1985). The role of the chlorine substituents in the antibiotic vancomycin—preparation and characterization of monodechlorovancomycin and didechlorovancomycin. J. Am. Chem. Soc..

[24] Groll M, Huber R, Potts BC (2006). Crystal structures of Salinosporamide A (NPI-0052) and B (NPI-0047) in complex with the 20S proteasome reveal important consequences of beta-lactone ring opening and a mechanism for irreversible binding. J. Am. Chem. Soc..

[25] Jia F (2019). The effect of halogenation on the antimicrobial activity, antibiofilm activity, cytotoxicity and proteolytic stability of the antimicrobial peptide Jelleine-I. Peptides.

[26] Cruz JC (2015). Brominated variant of the lantibiotic NAI-107 with enhanced antibacterial potency. J. Nat. Prod..

[27] Czyzewski AM (2016). In vivo, in vitro, and in silico characterization of peptoids as antimicrobial agents. PLoS ONE.

[28] Lee J (2018). Effect of side chain hydrophobicity and cationic charge on antimicrobial activity and cytotoxicity of helical peptoids. Bioorg. Med. Chem. Lett..

[29] Gentry CL (1999). The effect of halogenation on blood-brain barrier permeability of a novel peptide drug. Peptides.

[30] Häffner SM, Malmsten M (2018). Influence of self-assembly on the performance of antimicrobial peptides. Curr. Opin. Colloid Interface Sci..

[31] Tian X, Sun F, Zhou XR, Luo SZ, Chen L (2015). Role of peptide self-assembly in antimicrobial peptides. J. Pept. Sci..

[32] Chu-Kung AF, Nguyen R, Bozzelli KN, Tirrell M (2010). Chain length dependence of antimicrobial peptide-fatty acid conjugate activity. J. Colloid Interface Sci..

[33] Xu D (2015). Designed supramolecular filamentous peptides: Balance of nanostructure, cytotoxicity and antimicrobial activity. Chem. Commun..

[34] Chongsiriwatana NP (2017). Intracellular biomass flocculation as a key mechanism of rapid bacterial killing by cationic, amphipathic antimicrobial peptides and peptoids. Sci. Rep..

[35] Chongsiriwatana NP (2008). Peptoids that mimic the structure, function, and mechanism of helical antimicrobial peptides. Proc. Natl. Acad. Sci. USA.

[36] Frederiksen N, Hansen PR, Bjorkling F, Franzyk H (2019). Peptide/peptoid hybrid oligomers: The Influence of hydrophobicity and relative side-chain length on antibacterial activity and cell Selectivity. Molecules.

[37] Chen Y (2007). Role of peptide hydrophobicity in the mechanism of action of alpha-helical antimicrobial peptides. Antimicrob. Agents Chemother..

[38] Schroer MA, Svergun DI (2018). Recent developments in small-angle X-ray scattering and hybrid method approaches for biomacromolecular solutions. Emerg. Top Life Sci..

[39] Lund R, Shu J, Xu T (2013). A small-angle X-ray scattering study of α-helical bundle-forming peptide–polymer conjugates in solution: Chain conformations. Macromolecules.

[40] Narayanan TWH (2017). Recent applications of synchrotron radiation and neutrons in the study of soft matter. Crystallogr. Rev..

[41] Nielsen JE, Bjornestad VA, Lund R (2018). Resolving the structural interactions between antimicrobial peptides and lipid membranes using small-angle scattering methods: The case of indolicidin. Soft Matter.

[42] Lindner P, Zemb Th (2002). Neutrons, X-rays, and Light: Scattering Methods Applied to Soft Condensed Matter.

[43] Standards, N. C. f. C. L. Methods for dilution antimicrobial susceptibility tests for bacteria that grow aerobically, Approved standard (2012).

[44] Vandecandelaere I, Van Nieuwerburgh F, Deforce D, Nelis HJ, Coenye T (2014). Draft genome sequence of methicillin-resistant *Staphylococcus epidermidis* strain ET-024, isolated from an endotracheal tube biofilm of a mechanically ventilated patient. Genome Announc..

[45] Pernot P (2013). Upgraded ESRF BM29 beamline for SAXS on macromolecules in solution. J. Synchrotron Radiat..

[46] Round A (2015). BioSAXS sample changer: a robotic sample changer for rapid and reliable high-throughput X-ray solution scattering experiments. Acta Crystallogr. D Biol. Crystallogr..

[47] de Maria AA (2015). ISPyB for BioSAXS, the gateway to user autonomy in solution scattering experiments. Acta Crystallogr. D Biol. Crystallogr..

[48] Mouritzen MV (2018). Neurotensin, substance P, and insulin enhance cell migration. J. Pept. Sci..

[49] Nielsen JE (1861). A biophysical study of the interactions between the antimicrobial peptide indolicidin and lipid model systems. BBA Biomembranes.

