# Supplementary material for: Halogenation as a tool to tune antimicrobial activity of peptoids
Source: Sci Rep. 2020 Sep 9;10:14805. doi: 10.1038/s41598-020-71771-8 (PMC7481290; doi:10.1038/s41598-020-71771-8)

Halogenation as a tool to tune antimicrobial activity of peptoids

Natalia Molchanova[1,3], Josefine Eilsø Nielsen[2,3], Kristian B. Sørensen[1], Bala Krishna Prabhala[5], Paul Robert Hansen[4], Reidar Lund[2], Annelise E. Barron[6*], Håvard Jenssen[1*]

[1]Department of Science and Environment, Roskilde University, 4000 Roskilde, Denmark

[2]Department of Chemistry, University of Oslo, 0315 Oslo, Norway

[3]The Molecular Foundry, Lawrence Berkeley National Laboratory, Berkeley, California, USA

[4]Department of Drug Design and Pharmacology, Faculty of Health and Medical Sciences, University of Copenhagen, 2100 Copenhagen, Denmark

[5]Institute of Physics, Chemistry and Pharmacy, Section for Pharmacy, University of Southern Denmark,

Odense, Denmark

[6]Department of Bioengineering, School of Medicine and School of Engineering, Stanford University, Stanford, California, USA 94305

*Co-corresponding authors: Annelise Barron: aebarron@stanford.edu, Håvard Jenssen: jenssen@ruc.dk

Supplementary information

Theoretical modelling of data from SAXS:

Random polymer-like chains with fiber-like clusters

In order to extract accurate and detailed structural information, the SAXS data for the pure peptoids chains were analysed using a combination of free chains and rectangular fibres characterized by dimensions a< b<c where c is the length of the fibers and a and b is the X and Y direction of the cross section.[1]

$$I(q) = \phi \cdot V_p \cdot \Delta\rho^2 \cdot (P_{chain}(q) \cdot f_{chain} + N_p \cdot P_{sheet}(q) \cdot (1 - f_{chain})) \tag{1}$$

where ϕ is the volume fraction of the polymer, V_p is the volume of the polymer, Δρ is the excess scattering length density and f_{chain} is the fraction of free chains. N_p, the average number of peptides in each sheet, is defined as $N_p = \frac{abc}{V_p}$. $P_{chain}(q)$ is the form factor of the free peptoid chains given by the Debye expression for Gaussian chains:

$$P_{chain}(q) = \frac{2 \cdot exp\left[-\left(qR_g\right)^2\right] - 1 + \left(qR_g\right)^2}{\left(qR_g\right)^4} \tag{2}$$

where R_g is the radius gyration of the peptoid chains.

Under the assumption that the length of the peptoid sheets are much greater than the lateral dimension, i.e. c>>a,b, the form factor $P_{sheet}(q)$ is given by

$$P_{sheet}(q) = F_c(q) \frac{1}{2\pi} \int_0^{2\pi} A_{sheet}(q, \alpha)^2 \, d\alpha \tag{3}$$

where the amplitude is given by

$$A_{sheet}(q,\alpha) = \frac{sin(qb\ cos(\alpha)/2)}{qb\ cos(\alpha)/2} \cdot \frac{sin(qa\ sin(\alpha)/2)}{qa\ sin(\alpha)/2)} \tag{4}$$

and

$$F_c(q) = \ (2\,Si(qc)/(qc) - 4\,sin^2(qc/2)/(qc)^2 \tag{5}$$

where $\mathrm{Si}(x) = \int_0^x t^{-1} \sin t\ \mathrm{dt}$.

Self assembled peptides in solution: Peptide cylindrical bundle model

Each peptoid involved in the bundle is approximated as a simple solid cylinder given by[2]:

$$P(q)_{cyl} \ = \ \int_0^{\pi/2} \left|A(q,\alpha)_{cyl}\right|^2 \sin\alpha\ d\alpha\,; A(q,\alpha)_{cyl} = \frac{2J_1(qR\sin\alpha)}{qR\sin\alpha}\frac{\sin(qL\cos\alpha/2)}{qL\cos\alpha/2} \tag{6}$$

where L is the total length of the cylinder, R is the radius and α is the angle between the momentum transfer vector q and the cylinder axis parallel to L. J_1 is the first order Bessel function.

The form factor describing the scattering for a bundle consisting of parallel cylinders can be calculated using the expression given by Oster and Riley[3]:

$$P(q)_{bund} = \frac{P(q)_{cyl}}{N_{cyl}^2} \sum_{i=1}^{N_{cyl}} \sum_{j=1}^{N_{cyl}} J_0\left(qd_{ij}\right) \tag{7}$$

J_0 is the zeroth order Bessel function and the d_{ij} is the distance between the centers of the different cylinders. The above expression gives a generic expression that can, in principle, be evaluated for an arbitrary collection and number of cylinders. In this work we have used tetrameric bundle of cylinders where we assume that the bundles are arranged in a square with the center of each cylinder located in each corner. All inter-cylinder distances are then $d = 2fR$ except for the diagonal distance which is $d = \sqrt{8}fR$, where f is a swelling factor that regulates the distance between the cylinders. Equation 7 can be rewritten in terms of a form factor, $P(q)_{cyl}$ and the structure factor $S(q)_{bund0}^{(i)}$ as follows:

$$P(q)^{(i)}_{bund} = P(q)_{cyl} S(q)^{(i)}_{bund0};$$

$$S(q)^{(i)}_{bund0} = \frac{1}{16}\left(4 + 8J_0(2qRf) + 4J_0\left(\sqrt{8}qRf\right)\right) \quad (8)$$

The interaction between peptoid bundles seen at some of the highest concentrations, can be modeled using an expression from the polymer reference interaction site model (PRISM) given by the structure factor[4]:

$$S_{PRISM}(q) = \frac{1}{1 + \nu c(q) P_{cyl}(q)} \quad (9)$$

Where ν is a measure of the excluded volume and c(q) is the form factor of an infinitely thin rod[5] :

$$P_{rod}(q) = 2Si\frac{qL}{qL} - \frac{4\sin^2(qL/2)}{q^2L^2} \quad (10)$$

The total expression for the intensity is given by:

$$I = \frac{\phi}{V_p} \cdot V_p^2 \cdot \Delta\rho^2 P_{bound}(q) \cdot S_{PRISM}(q) \cdot f_{agg} + \left(1 - f_{agg}\right) \cdot P_{chain}(q) \quad (11)$$

Where f_{agg} is the fraction of peptoid chains aggregated in bundles, allowing for calculation of a CAC from $\phi_{CAC} = \phi - (f_{agg} * \phi)$.

Nanostructure of peptoids in solution- SAXS results

Figure S1. Full SAXS results for all 10-mers measured at ESRF in the indicated concentrations together with model fits (**19**-**21** and **24**-**27** is fitted using the Random polymer-like chains with fiber-like clusters model (eq, 1), while **22** and **23** is fitted using the bundle model (eq. 6)).

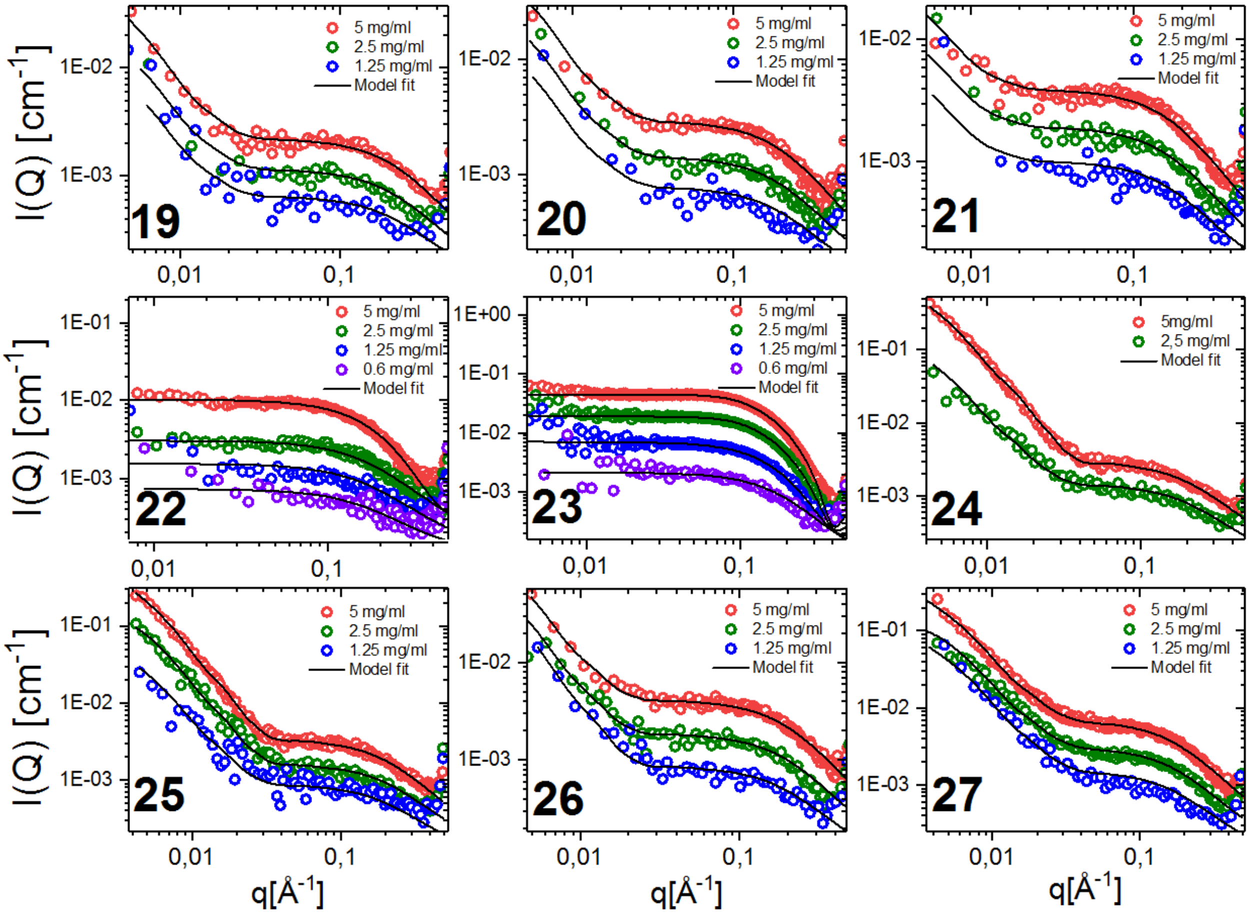

Table S1: Full fit parameters for the 10-mers.

Compound	CAC	Rg*	Fraction of sheets	Dimensions:	Model
**19**	>5mg/mL	6.8Å	<0.001 % sheets (consistent for concentration range 5-1.25mg/mL	Sheet dimensions: 560Å × 150Å × >600Å	Gaussian chains (with sheets)
**Fully halogenated:**					
**20**	>5mg/mL	7.5Å	<0.001 % sheets (consistent for concentration range 5-1.25mg/mL)	Sheet dimensions: 550Å × 145Å × >600Å	Gaussian chains (with sheets)
**21**	>5mg/mL	8.7Å	<0.0005 % sheets (consistent for concentration range 5-1.25mg/mL)	Sheet dimensions: 550Å × 190Å × >600Å	Gaussian chains (with sheets)
**22**	2.3 mg/mL	9.4Å	-		Bundles
**23**	0.4 mg/mL	9Å	-		Bundles
**Half halogenated:**					
**24**	>5mg/mL	7.7Å	0.006 % sheets at 5 mg/mL and 0.002 % for 2.5mg/mL	Sheet dimensions: 550Å × 150Å × >600Å	Gaussian chains (with sheets
**25**	>5mg/mL	7.7Å	0.004 % sheets at 5 mg/mL, 0.003 % for 2.5mg/mL and 0.001% for 1.25 mg/mL	Sheet dimensions: 580Å × 150Å × >600Å	Gaussian chains (with sheets
**26**	>5mg/mL	7.8Å	0.0005 % sheets at 5 mg/mL, 0.0006 % for 2.5mg/mL and 0.001% for 1.25 mg/mL	Sheet dimensions: 550Å × 200Å × >600Å	Gaussian chains (with sheets
**27**	>5mg/mL	9.1 Å	<0.002 % sheets (consistent for concentration range 5-1.25mg/mL)	Sheet dimensions: 550Å × 160Å × >600Å	Gaussian chains (with sheets

**The Rg of the fraction free Gaussian chains.*

Table S2: Full fit parameters for the fully Iodinated peptoids with increasing legth.

Compound	n-mers	CAC (mg/mL)	Rg* (Å)	R (Å)	L (Å)	v	fa	Model
**5**	6	2.8	8	3.7	24	0.2	7.1	Bundles
**14**	8	1.4	8	4.5	26	0.3-0.6	6.6	Bundles
**23**	10	0.5	9	4.2	34	0.3-1	6.7	Bundles
**32**	12	0.4	13	4	56	1-1.1	5.7	Bundles

**The Rg of the fraction free Gaussian chains.*

1 Nielsen, J. E., Bjornestad, V. A. & Lund, R. Resolving the structural interactions between antimicrobial peptides and lipid membranes using small-angle scattering methods: the case of indolicidin. *Soft Matter* **14**, 8750-8763, doi:10.1039/c8sm01888j (2018).

2 Lund, R., Shu, J., Xu, T. A small-angle X-ray scattering study of α-helical bundle-forming peptide–polymer conjugates in solution: chain conformations. Macromolecules 46 (4), 1625–1632, doi:10.1021/ma301310h (2013).

3 Oster, G., Riley, D.P. . Scattering from Cylindrically Symmetric Systems. *Acta Cryst* **5**, 272 (1951).

4 Schweizer, K. S., and J. G. Curro. PRISM theory of the structure, thermodynamics, and phase transitions of polymer liquids and alloys." Atomistic Modeling of Physical Properties. . *Atomistic Modeling of Physical Properties. Springer, Berlin, Heidelberg*, 319-377 (1994.).

5 Arleth L., B. M., Pedersen J. S. . Small-angle neutron scattering study of the growth behavior, flexibility, and intermicellar interactions of wormlike SDS micelles in NaBr aqueous solutions. *Langmuir.* **18(14)**, 5343-5353. (2002).

Compound 1. Charge: +4. t_R = 11.490 min. Gradient: 0-100% B over 20 min. B = 90% MeCN + 0.1% TFA. HRMS: Exact Mass 842.51668, Base peak ion: m/z 422.2891 $(M+2H)^{2+}$

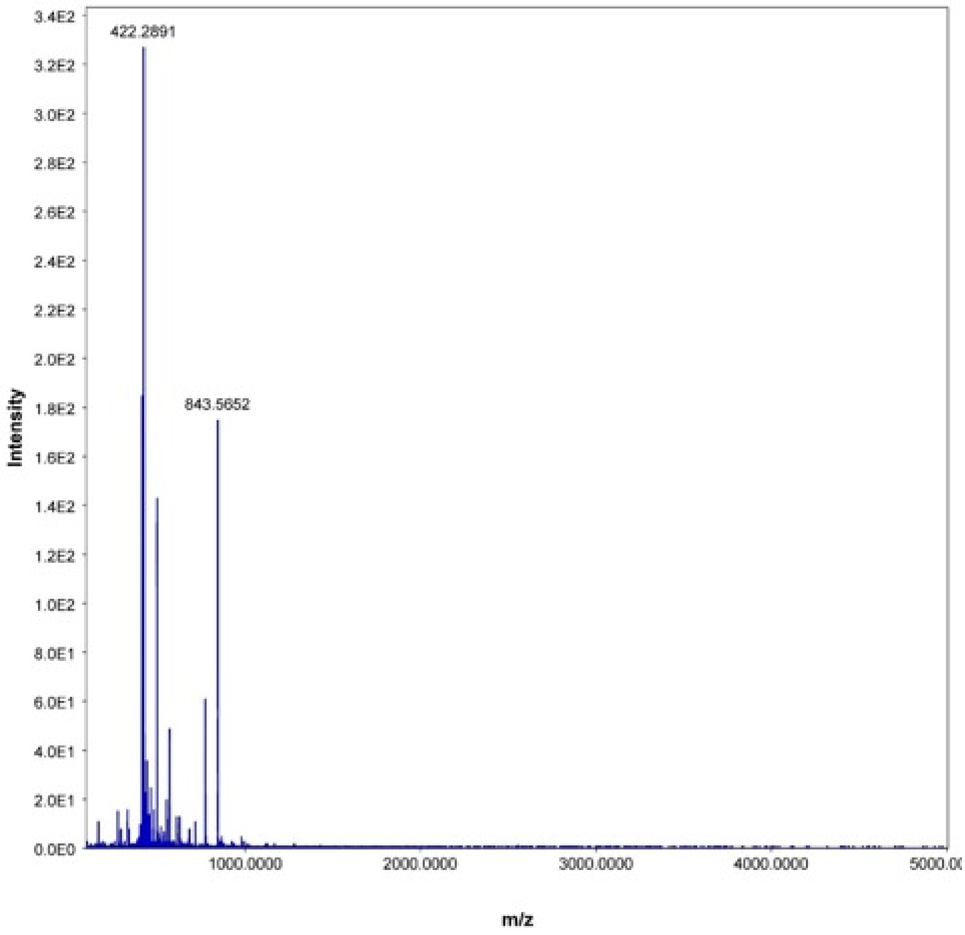

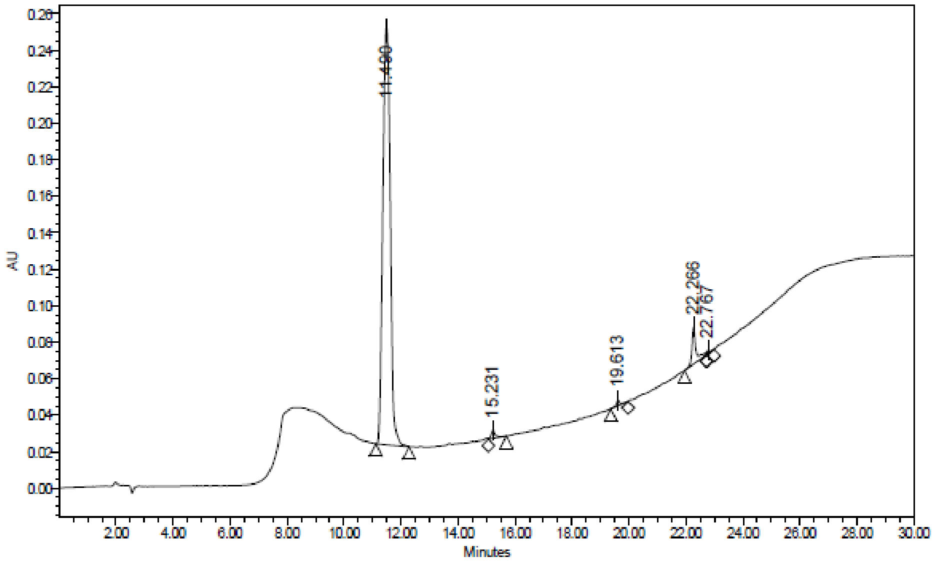

Peak Results

	Name	RT	Area	Height	% Area
1		11.49[illegible]	4136149	228477	93.45

Compound 2. Charge: +4. t_R = 12.080 min. Gradient: 0-100% B over 20 min. B = 90% MeCN + 0.1% TFA. HRMS: Exact Mass 896.4884, Base peak ion: m/z 449.2450 $(M+2H)^{2+}$

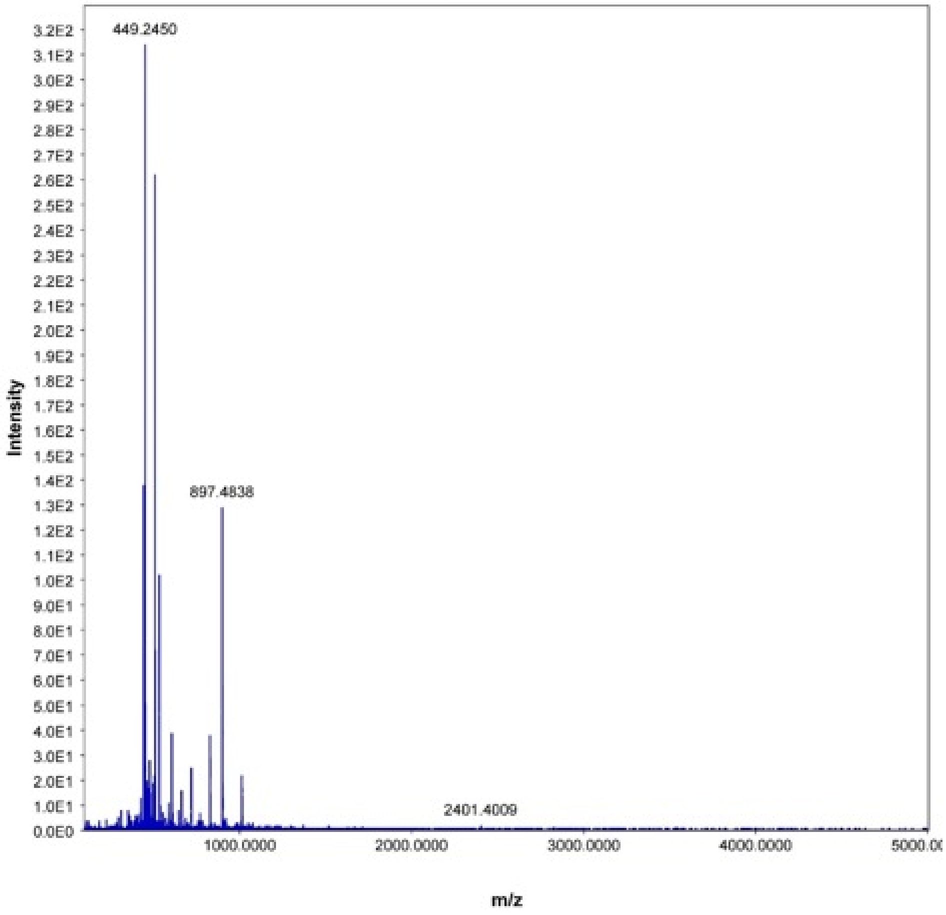

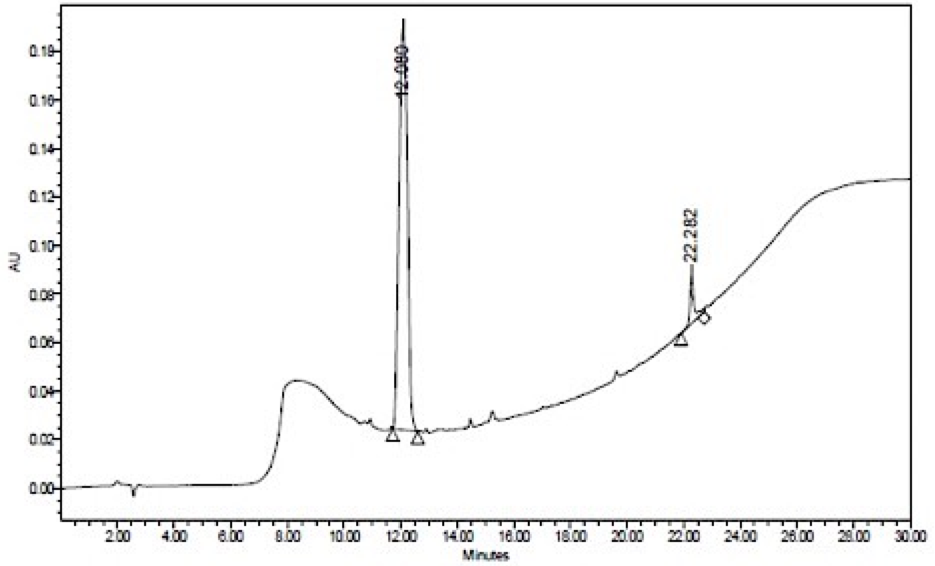

Peak Results

	Name	RT	Area	Height	% Area
1		12.080	3436821	165499	94.53

Compound 3. Charge: +4. t_R = 13.057 min. Gradient: 0-100% B over 20 min. B = 90% MeCN + 0.1% TFA. HRMS: Exact Mass: 944.3998, Base peak ion: m/z 473.218 $(M+2H)^{2+}$

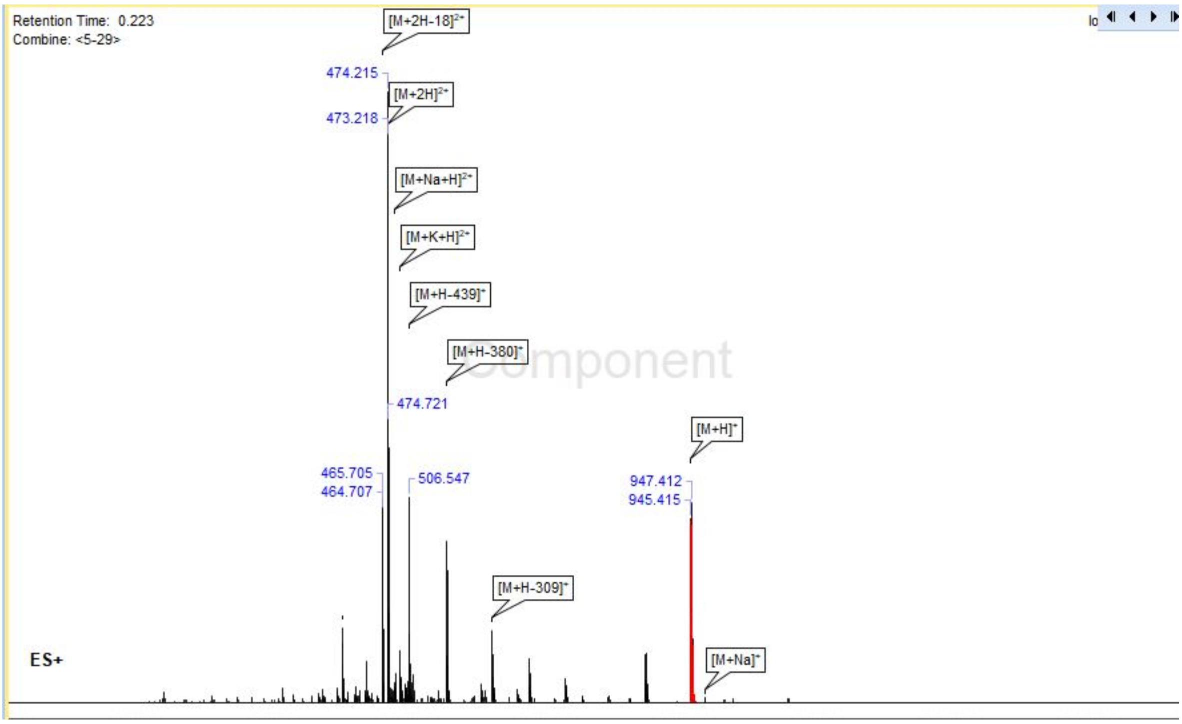

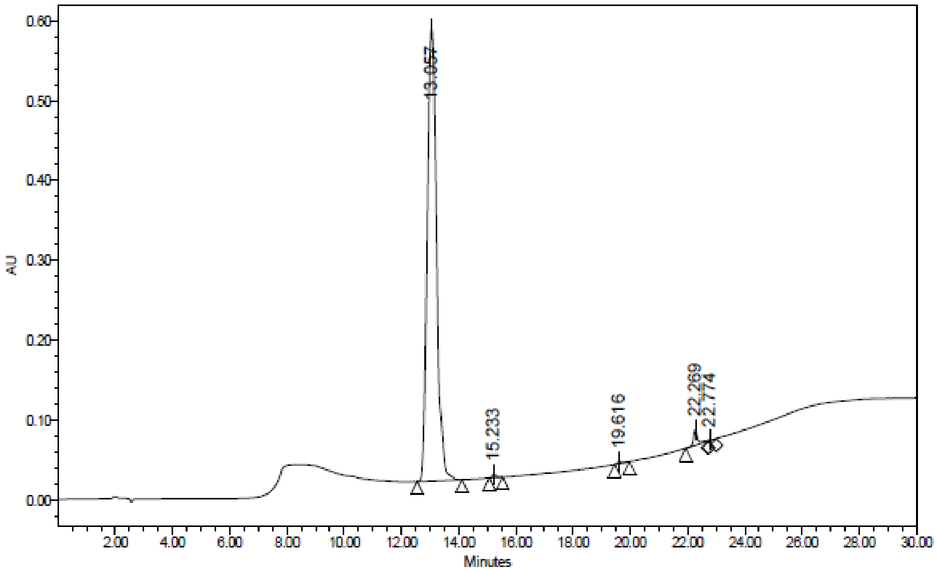

Peak Results

	Name	RT	Area	Height	% Area
1		13.057	12785624	567153	97.86

Compound 4. Charge: +4. t_R = 13.321 min. Gradient: 0-100% B over 20 min. B = 90% MeCN + 0.1% TFA. HRMS: Exact Mass: 1076.2482, Base peak ion: m/z 540.154 $(M+2H)^{2+}$

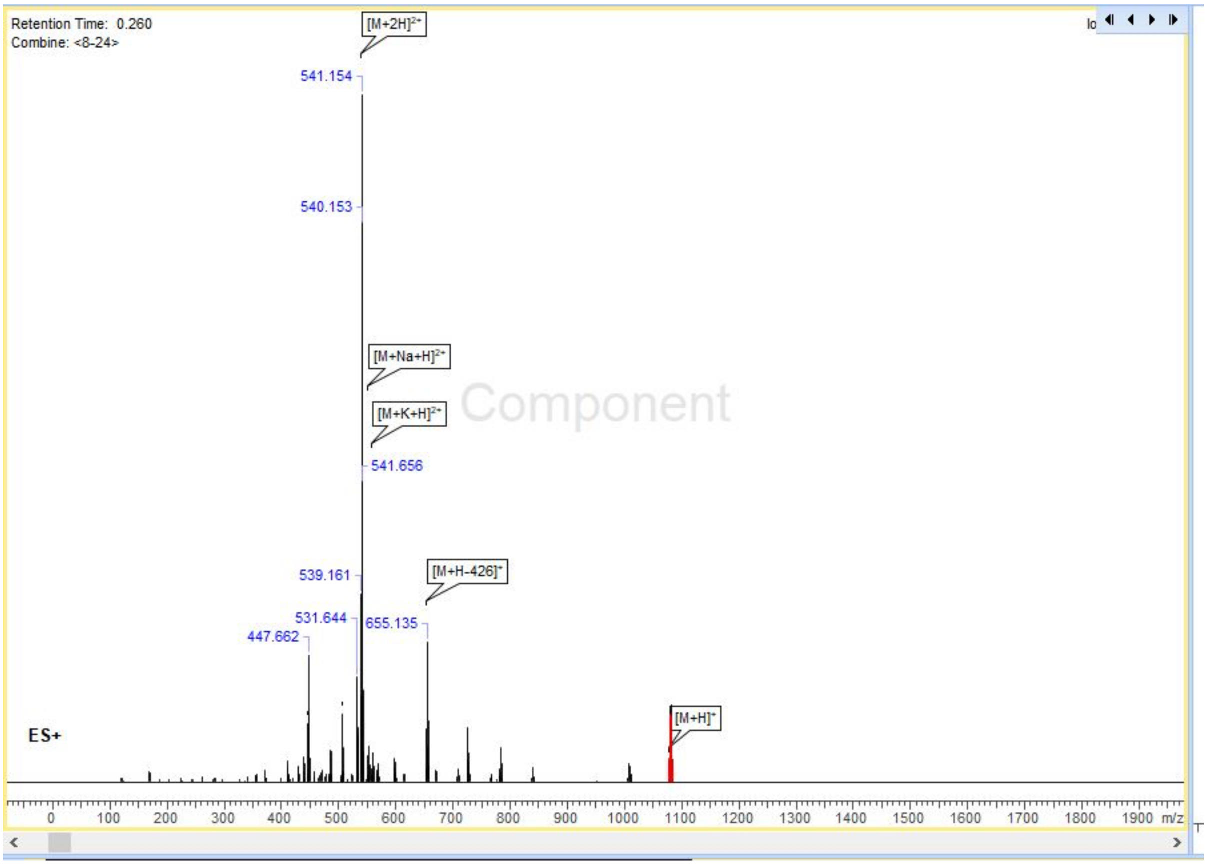

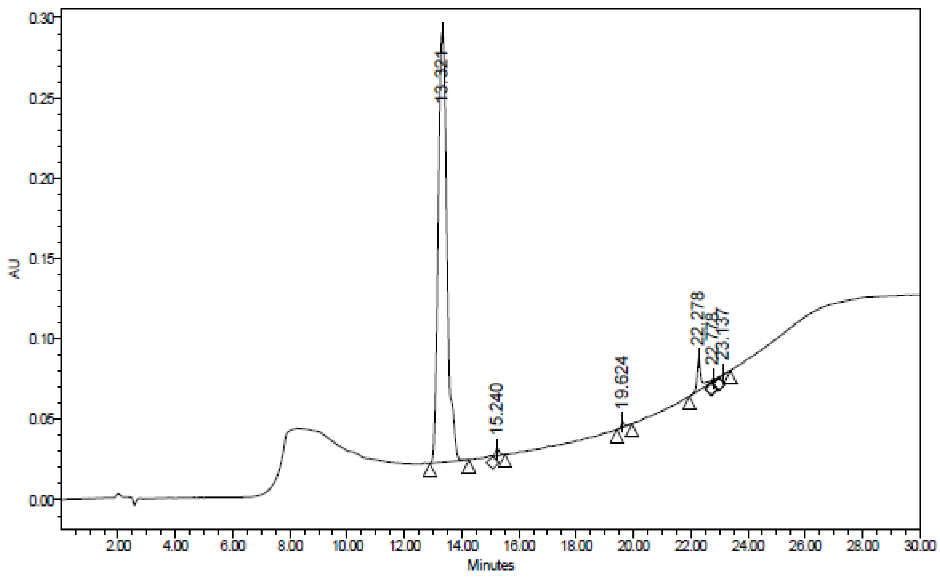

Peak Results

	Name	RT	Area	Height	% Area
1		13.321	5709782	267629	95.03

Compound 5. Charge: +4. t_R = 13.667 min. Gradient: 0-100% B over 20 min. B = 90% MeCN + 0.1% TFA. HRMS: Exact Mass: 1220.2066, Base peak ion: m/z 611.1436 $(M+2H)^{2+}$

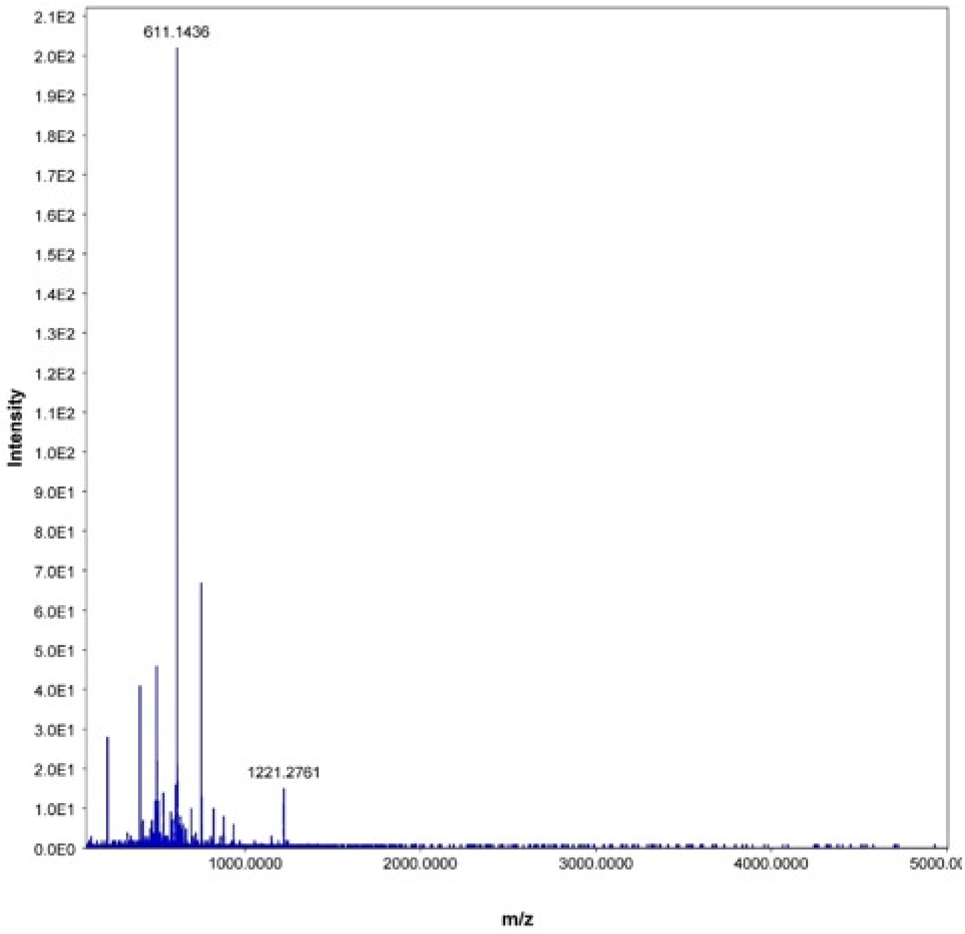

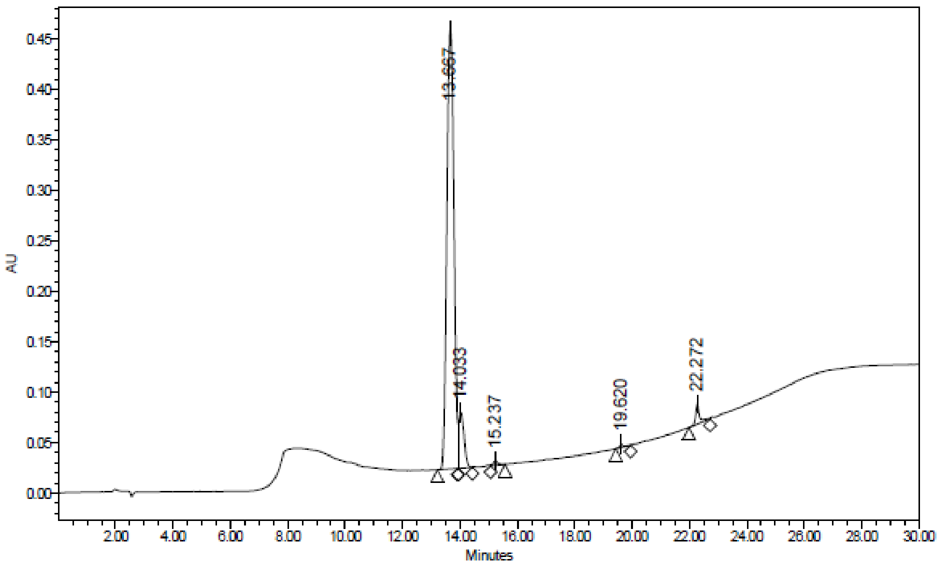

Peak Results

	Name	RT	Area	Height	% Area
1		13.667	7414283	434415	88.11
2		14.033	729726	54954	8.67

Compound 6. Charge: +4. t_R = 11.996 min. Gradient: 0-100% B over 20 min. B = 90% MeCN + 0.1% TFA. HRMS: Exact Mass: 878.4978, Base peak ion: m/z 879.3983 $(M+H)^+$

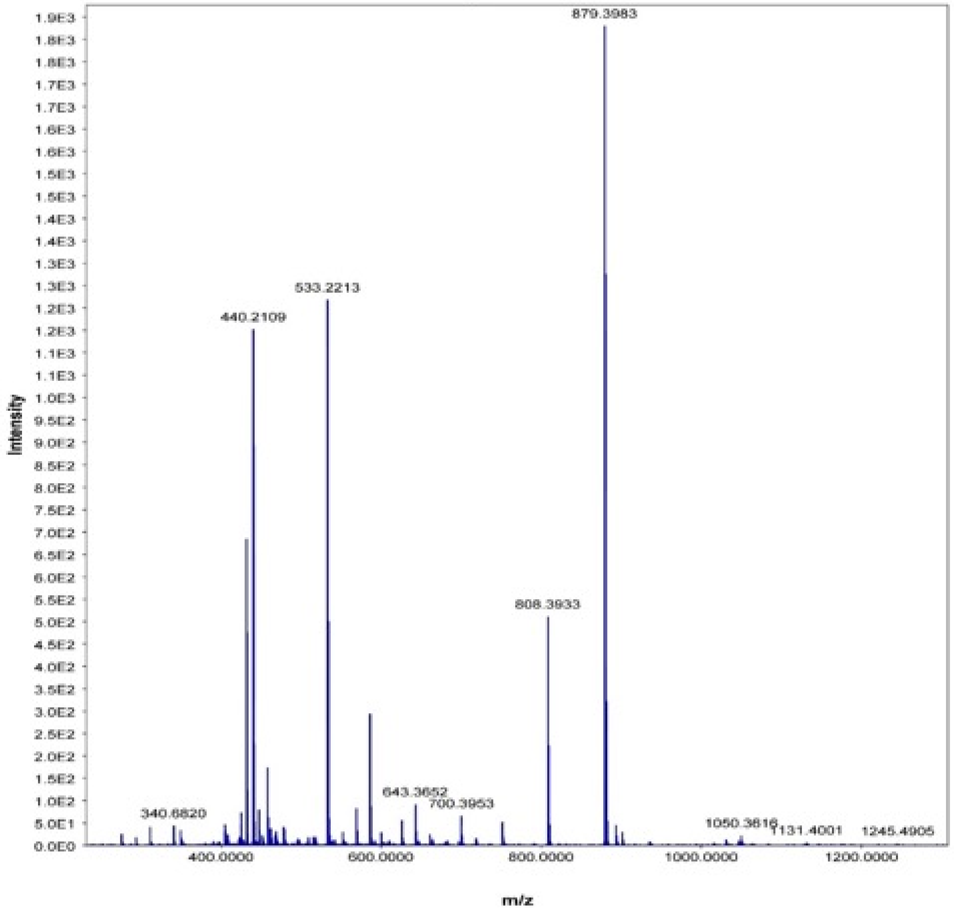

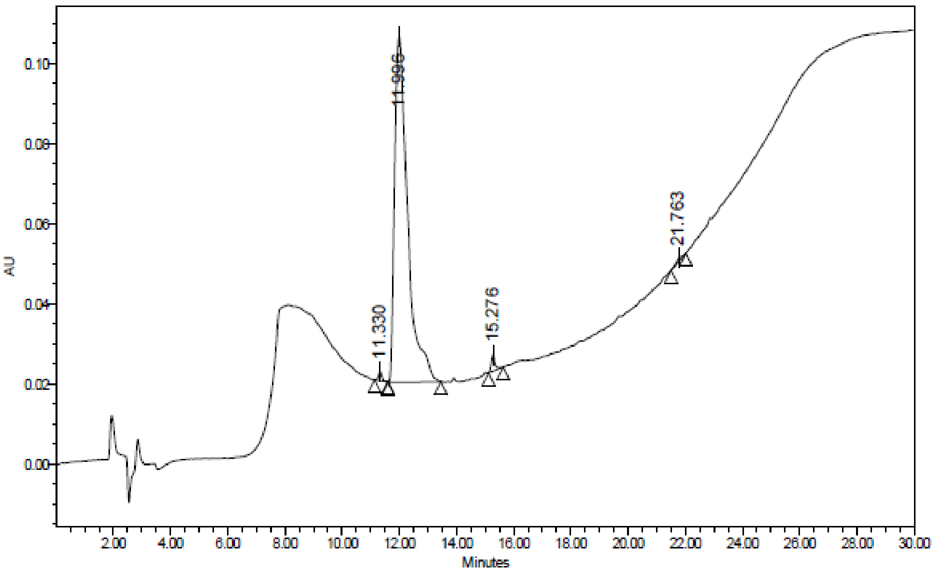

Peak Results

	Name	RT	Area	Height	% Area
1		11.330	18491	2320	0.66
2		11.996	2737996	86204	97.70

Compound 7. Charge: +4. t_R =12.526 min. Gradient: 0-100% B over 20 min. B = 90% MeCN + 0.1% TFA. HRMS: Exact Mass 910.4387, Base peak ion: m/z 456.2621 $(M+2H)^{2+}$

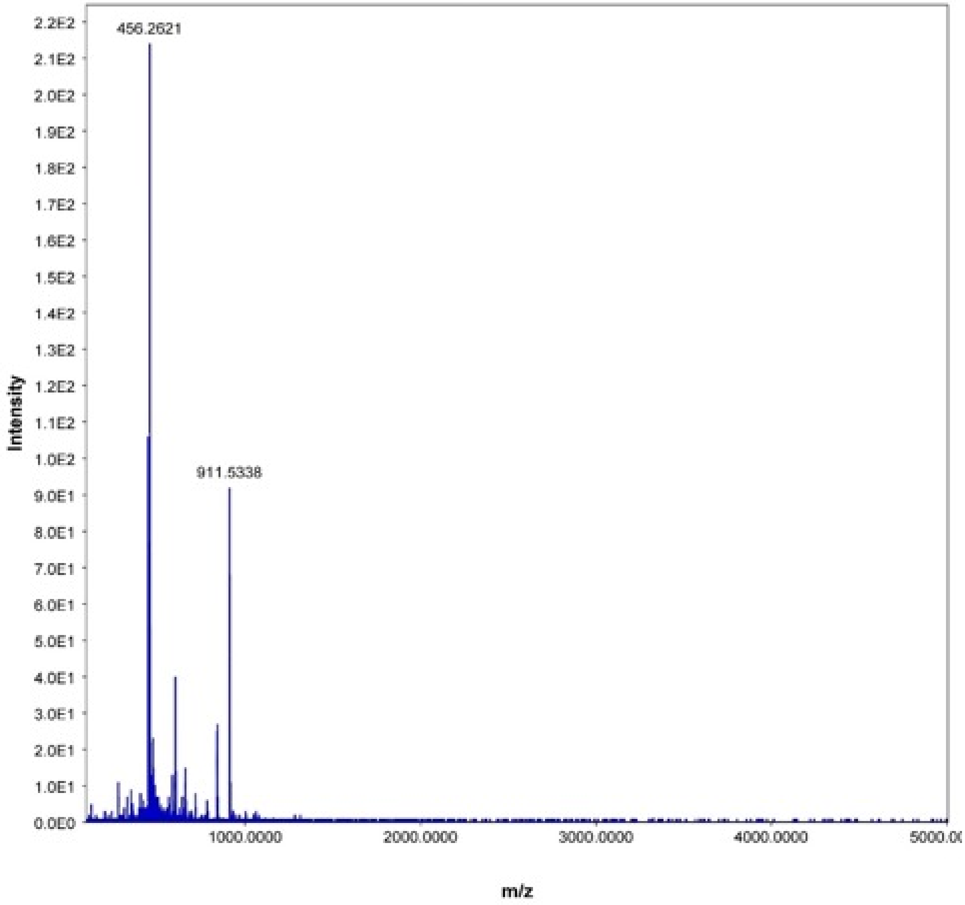

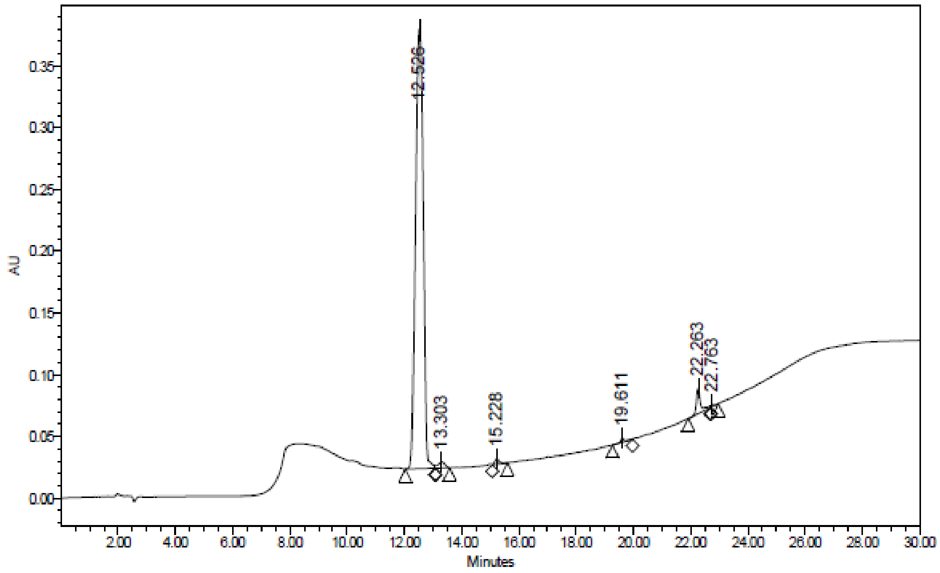

Peak Results

	Name	RT	Area	Height	% Area
1		12.526	6678167	356322	94.73

Compound 8. Charge: +4. t_R = 12.716 min. Gradient: 0-100% B over 20 min. B = 90% MeCN + 0.1% TFA. HRMS: Exact Mass: 998.3377, Base peak ion: m/z 500.235 $(M+2H)^{2+}$

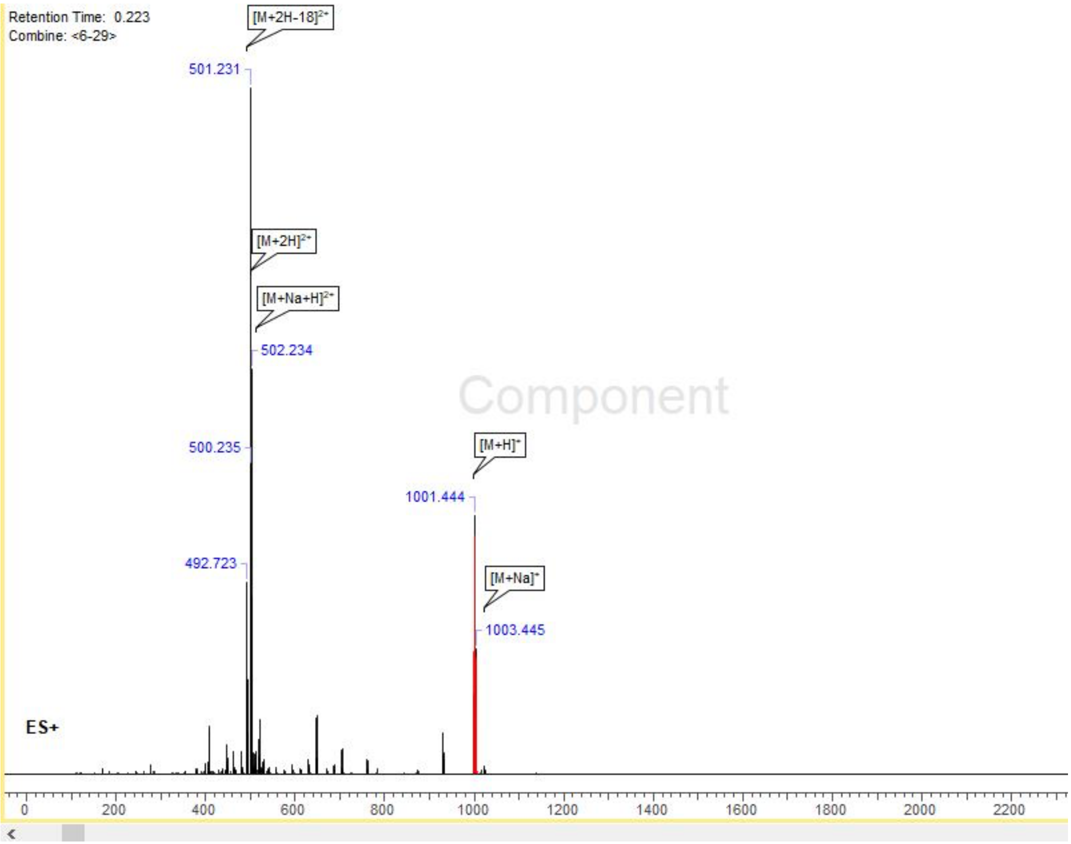

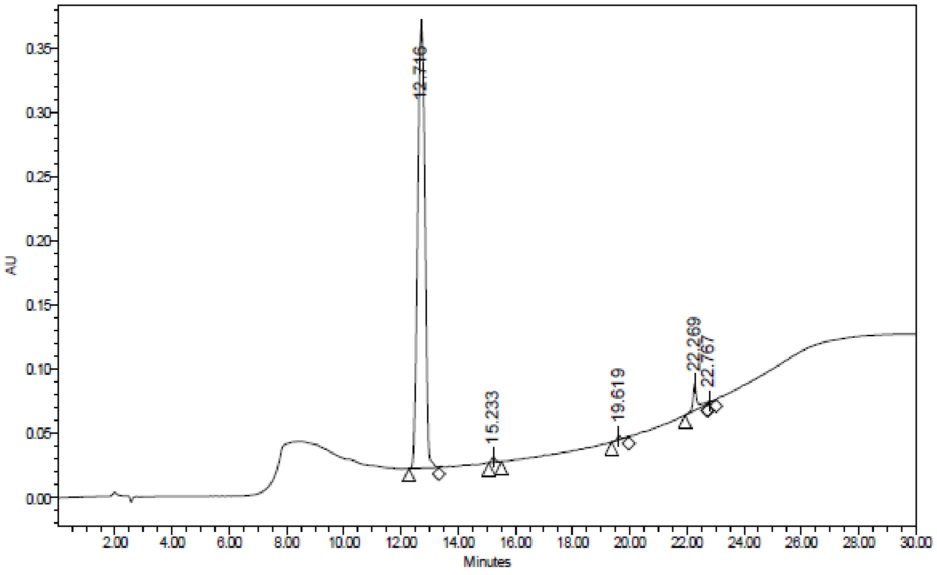

Peak Results

	Name	RT	Area	Height	% Area
1		12.716	6102819	341788	95.39

Compound 9. Charge: +4. t_R = 12.972 min. Gradient: 0-100% B over 20 min. B = 90% MeCN + 0.1% TFA. HRMS: Exact Mass: 1094.3100: Base peak ion: m/z 548.2147 $(M+2H)^{2+}$

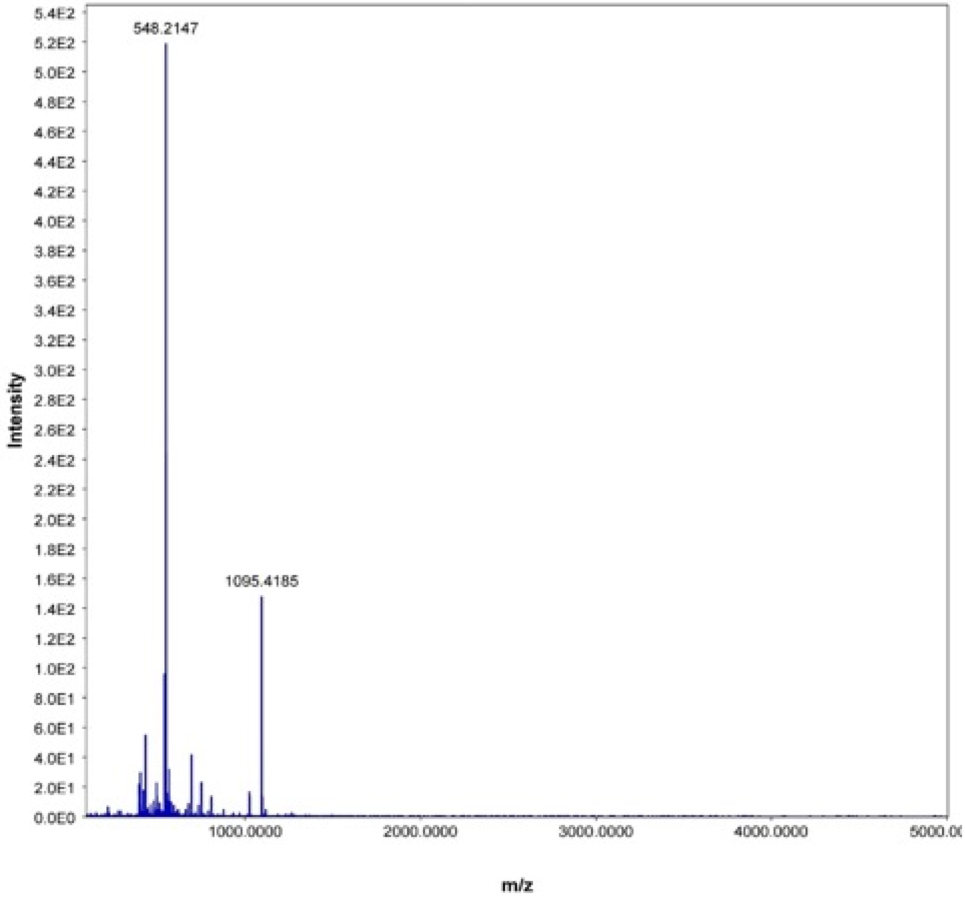

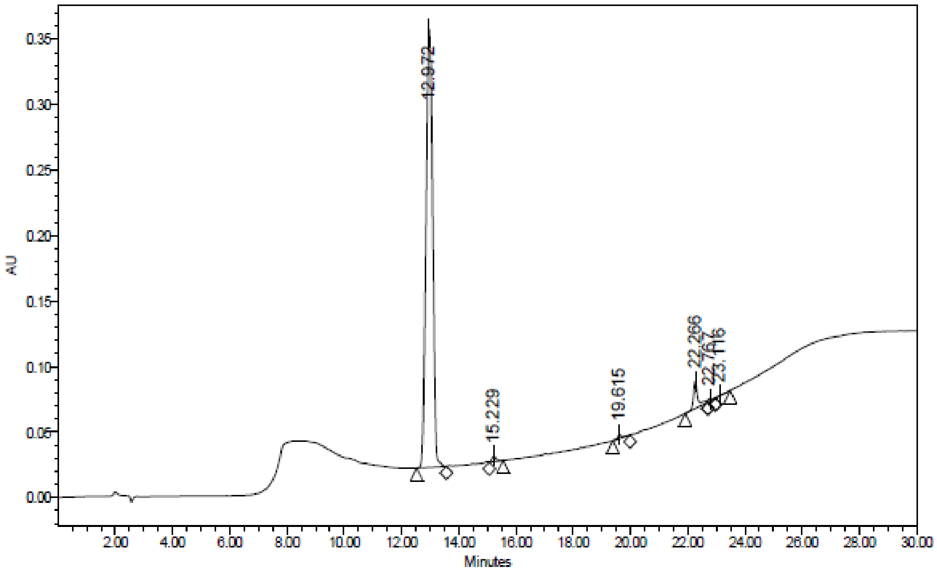

Peak Results

	Name	RT	Area	Height	% Area
1		12.972	5423357	335428	94.53

Compound 10. Charge: +5. t_R = 11.693 min. Gradient: 0-100% B over 20 min. B = 90% MeCN + 0.1% TFA. HRMS: Exact Mass: 1117.6801, Base peak ion: 559.9152 $(M+2H)^{2+}$

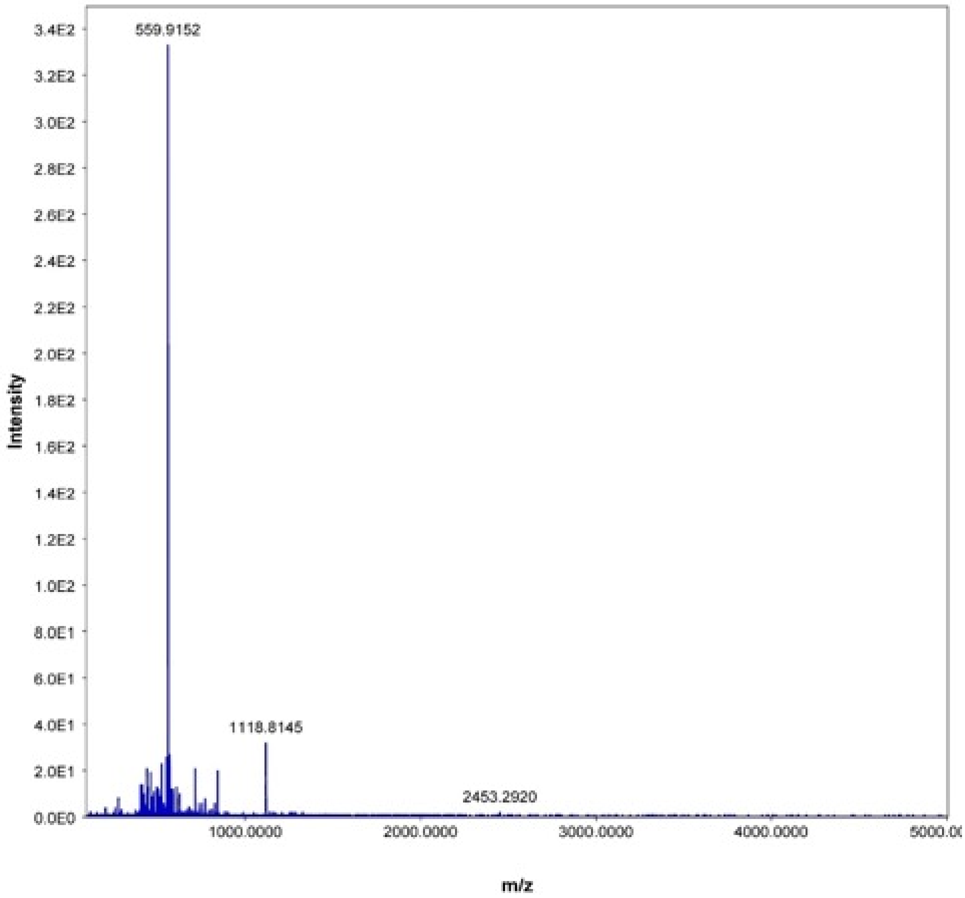

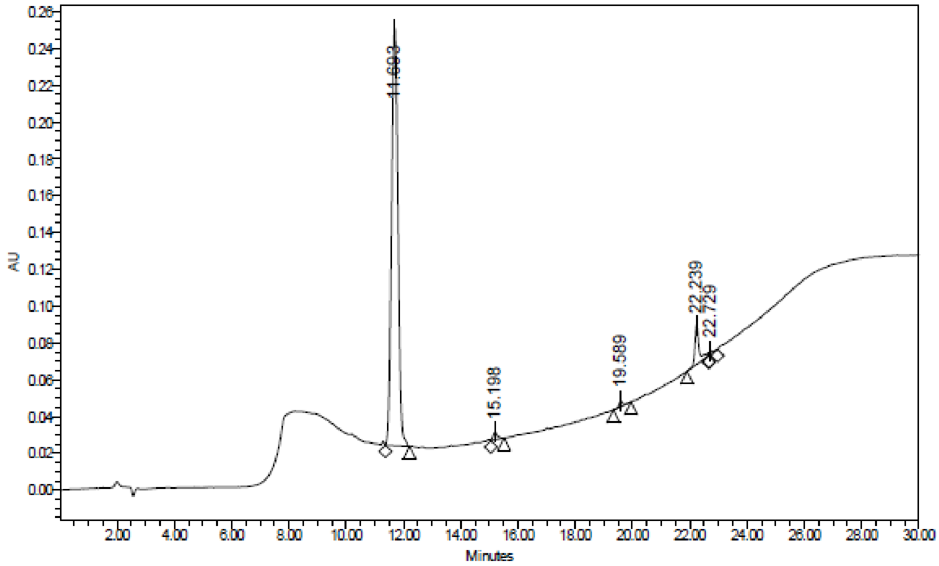

Peak Results

	Name	RT	Area	Height	% Area
1		11.693	3511602	227306	92.19

Compound 11. Charge: +5. t_R = 12.303 min. Gradient: 0-100% B over 20 min. B = 90% MeCN + 0.1% TFA. HRMS: Exact Mass: 1189.6424, Base peak ion: m/z 595.8840 $(M+2H)^{2+}$

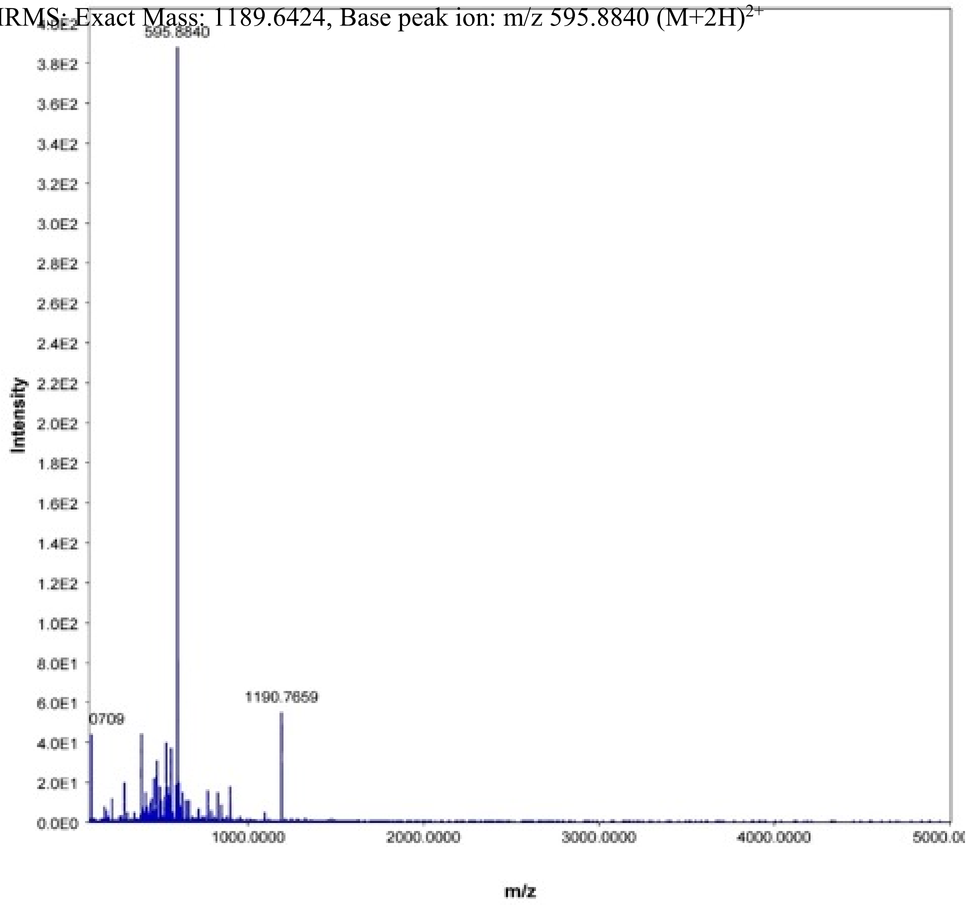

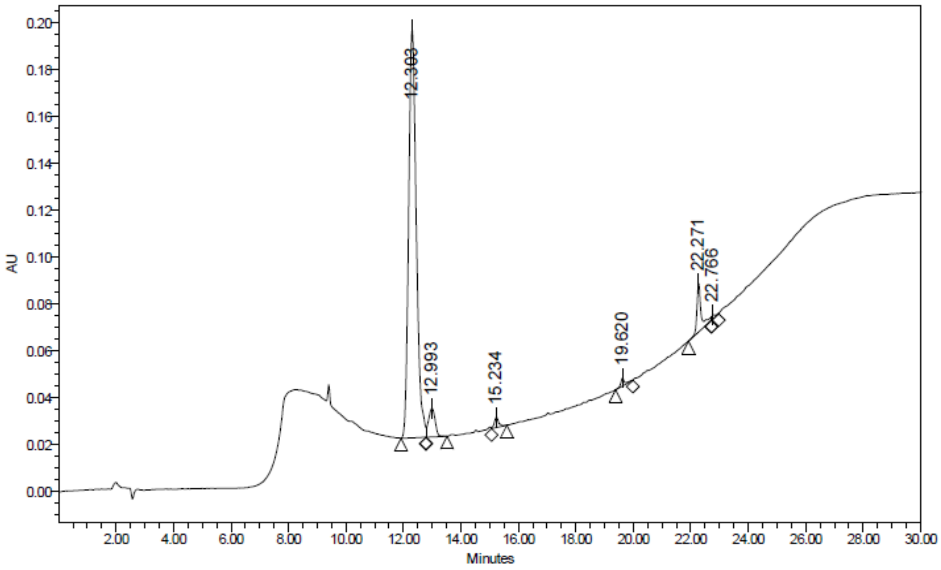

Peak Results

	Name	RT	Area	Height	% Area
1		12.303	3201802	174108	86.60

Compound 12. Charge: +5. t_R = 13.324 min. Gradient: 0-100% B over 20 min. B = 90% MeCN + 0.1% TFA. HRMS: Exact Mass: 1253.5242, Base peak ion: m/z 627.853 $(M+2H)^{2+}$

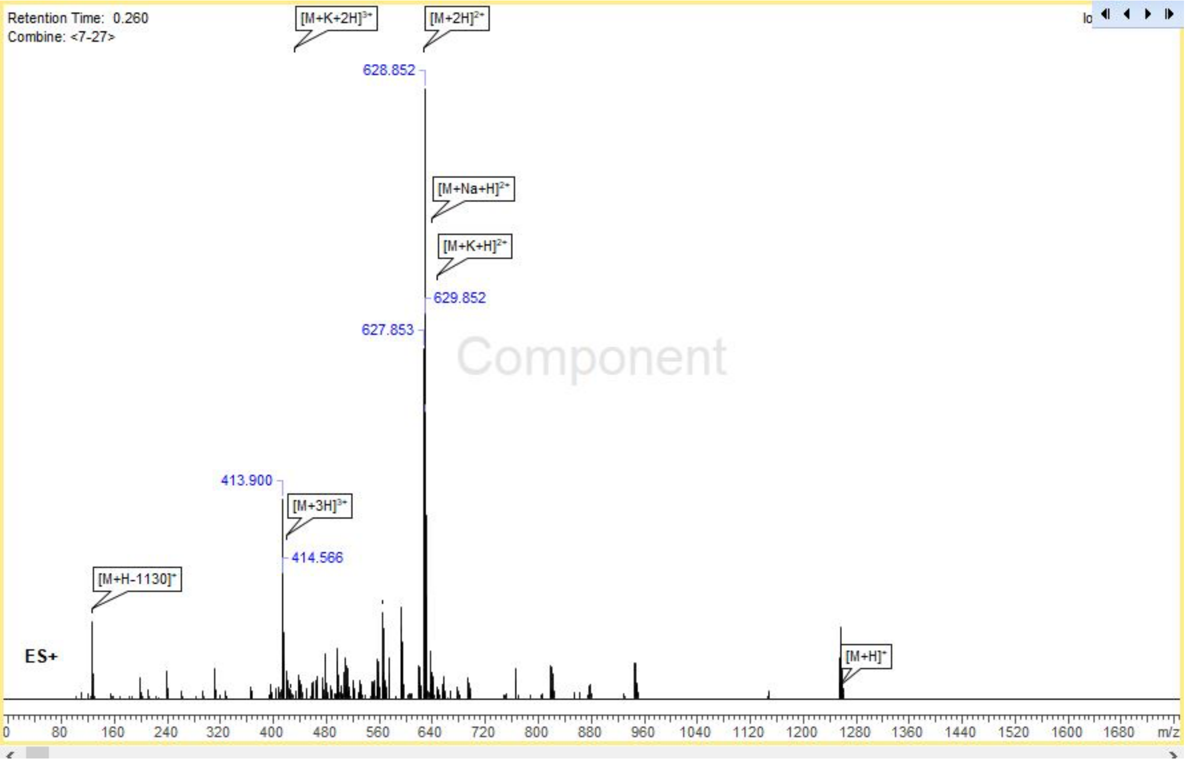

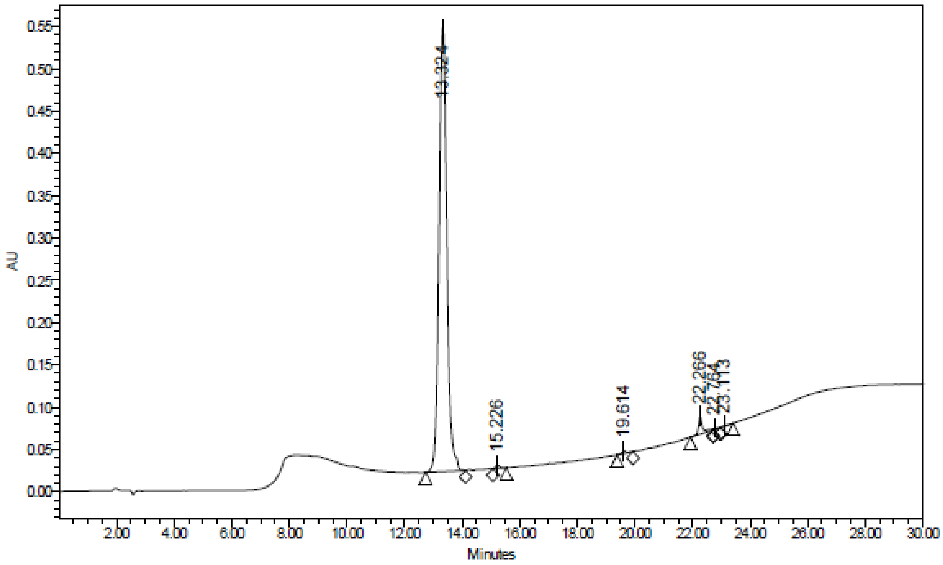

Peak Results

	Name	RT	Area	Height	% Area
1		13.324	9841160	524899	96.97

Compound 13. Charge: +5. t_R = 13.598 Gradient: 0-100% B over 20 min. B = 90% MeCN + 0.1% TFA. HRMS: Exact Mass: 1429.3221, Base peak ion: m/z 716.764 $(M+2H)^{2+}$

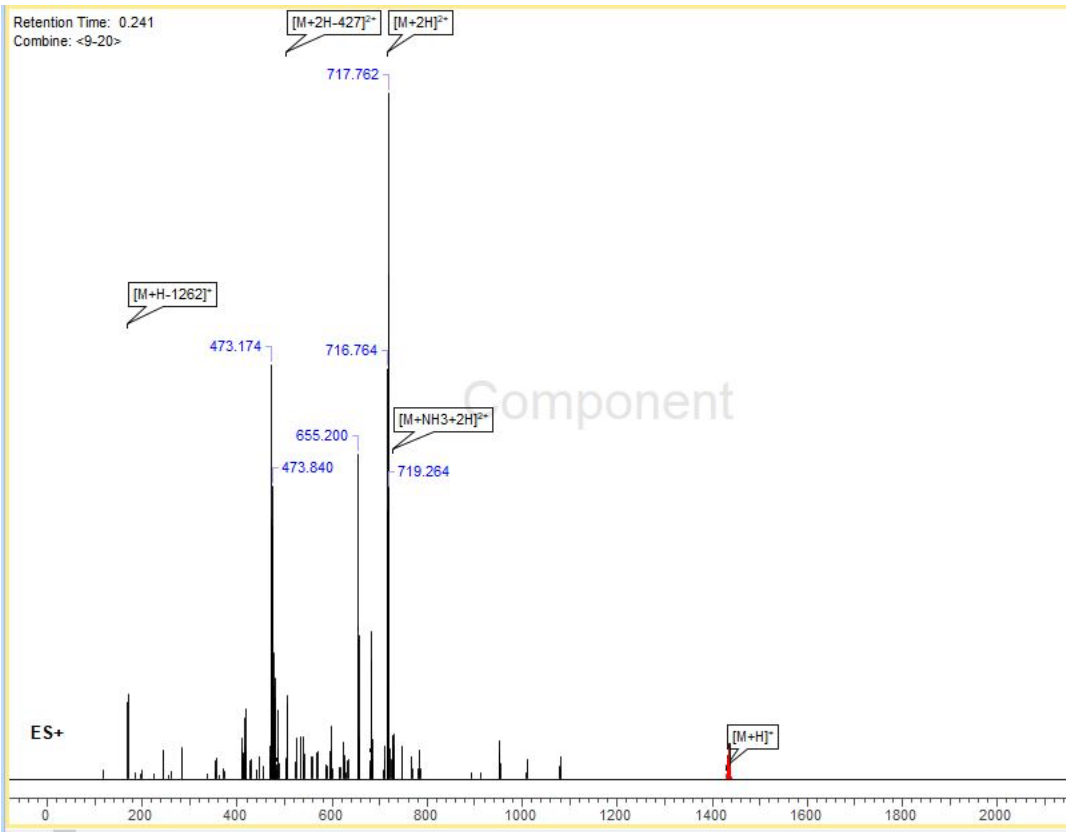

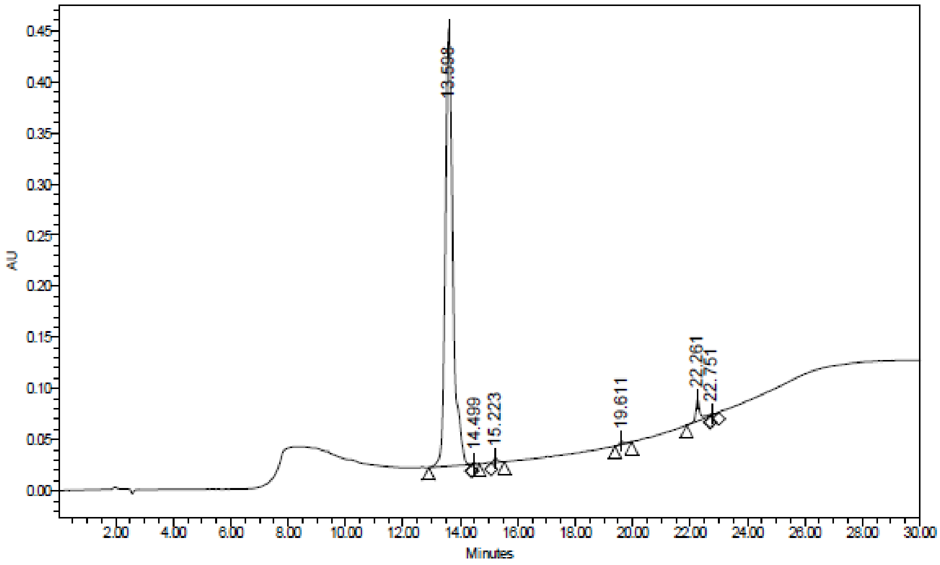

Peak Results

	Name	RT	Area	Height	% Area
1		13.598	7875186	428376	96.20

Compound 14. Charge: +5. t_R = 13.939 min. Gradient: 0-100% B over 20 min. B = 90% MeCN + 0.1% TFA. HRMS: Exact Mass: 1621.2666, Base peak ion: m/z 811.7253 $(M+2H)^{2+}$

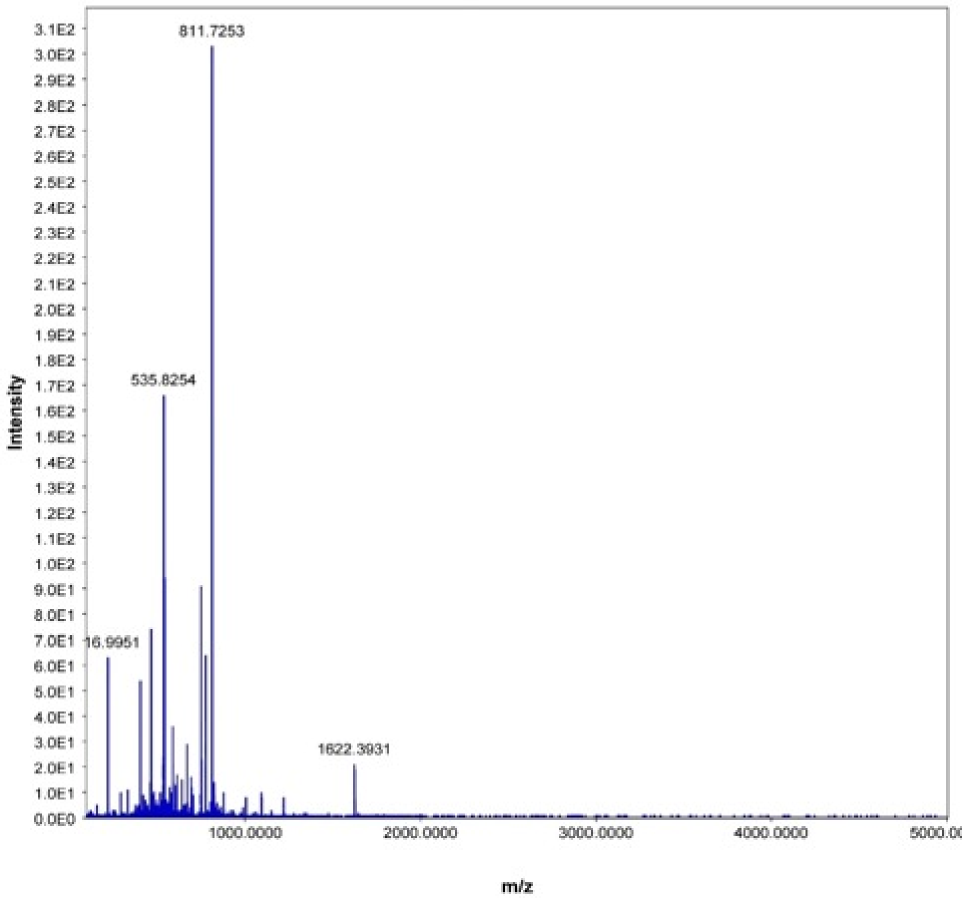

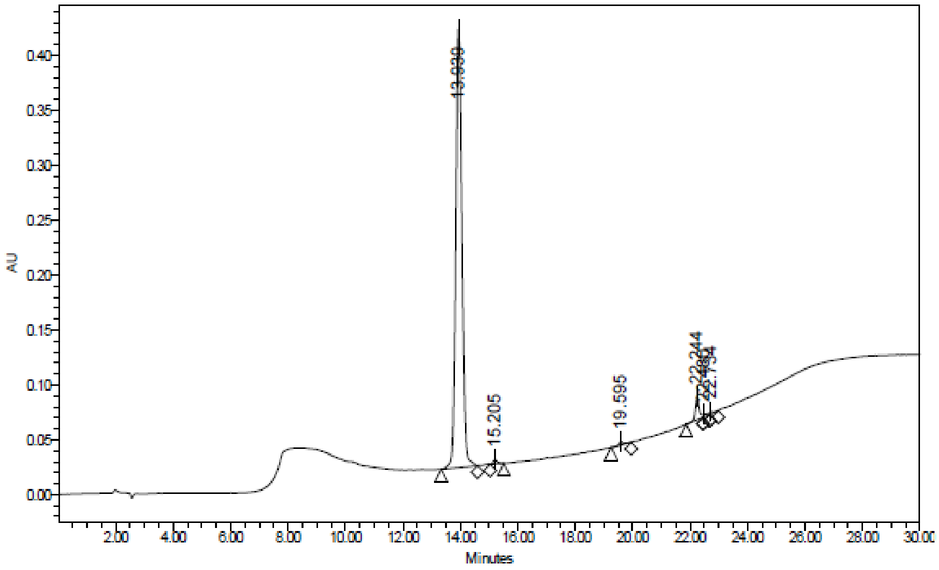

Peak Results

	Name	RT	Area	Height	% Area
1		13.939	5945259	400645	95.19

Compound 15. Charge: +5. t_R = 12.006 min. Gradient: 0-100% B over 20 min. B = 90% MeCN + 0.1% TFA. HRMS: Exact Mass: 1153.6612, Base peak ion: m/z 577.9105 $(M+2H)^{2+}$

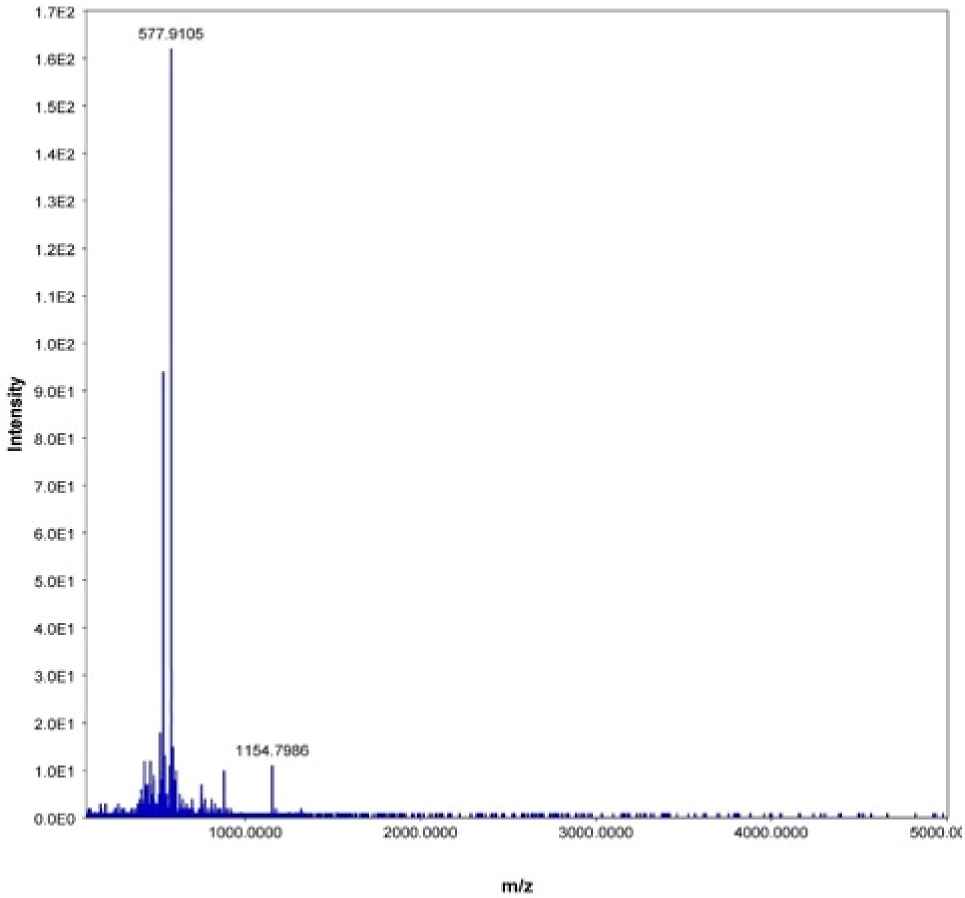

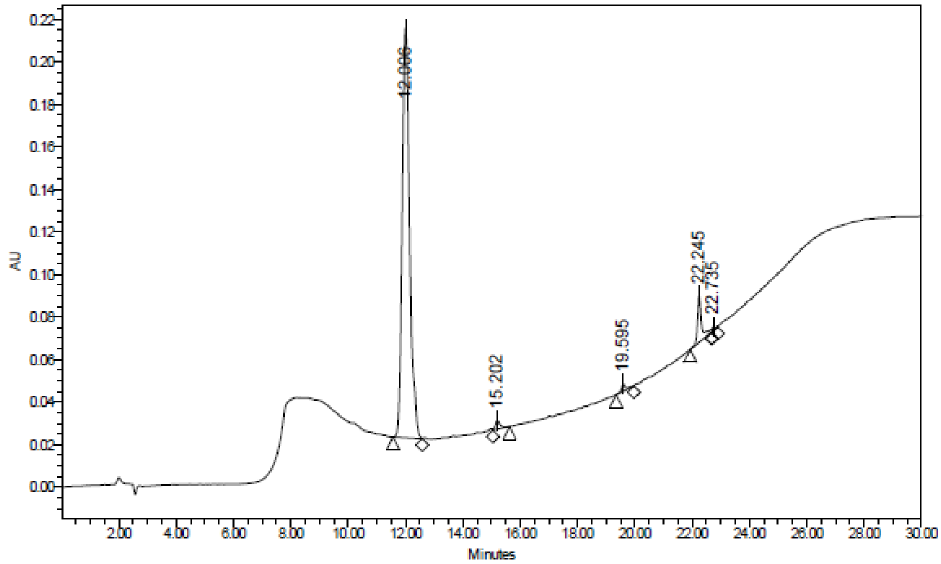

Peak Results

	Name	RT	Area	Height	% Area
1		12.006	3443437	192728	91.78

Compound 16. Charge: +5. t_R = 12.549 min. Gradient: 0-100% B over 20 min. B = 90% MeCN + 0.1% TFA. HRMS: Exact Mass: 1185.6021, Base peak ion: m/z 593. 8754 $(M+2H)^{2+}$

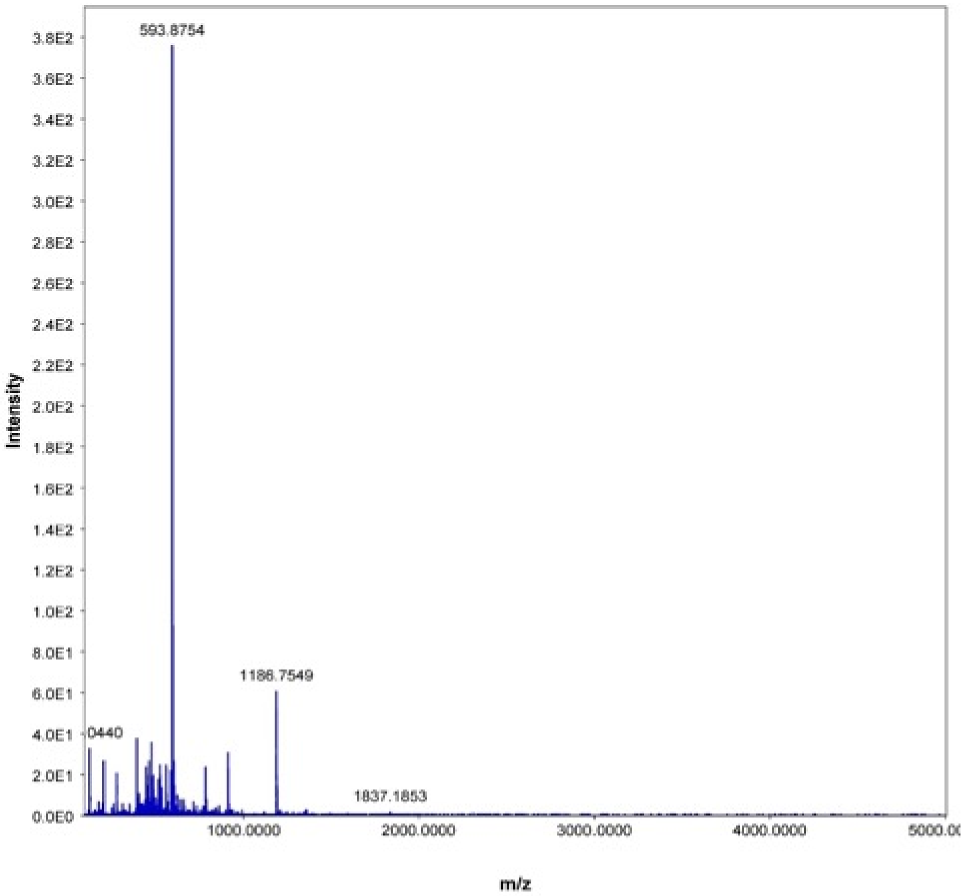

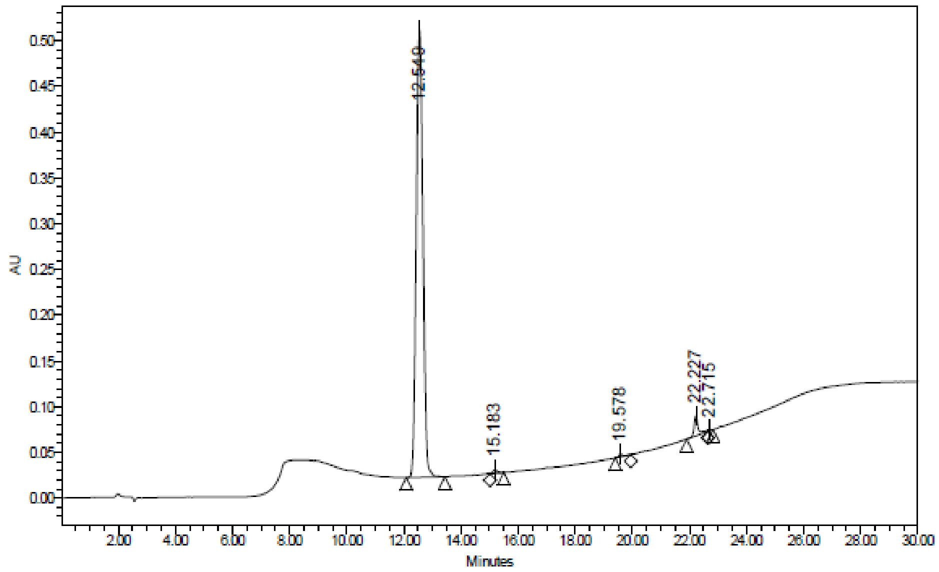

Peak Results

	Name	RT	Area	Height	% Area
1		12.549	7943127	489378	96.38

Compound 17. Charge: +5. t_R = 12.699 min. Gradient: 0-100% B over 20 min. B = 90% MeCN + 0.1% TFA. HRMS: Exact Mass: 1273.5011, Base peak ion: m/z 638.850 $(M+2H)^{2+}$

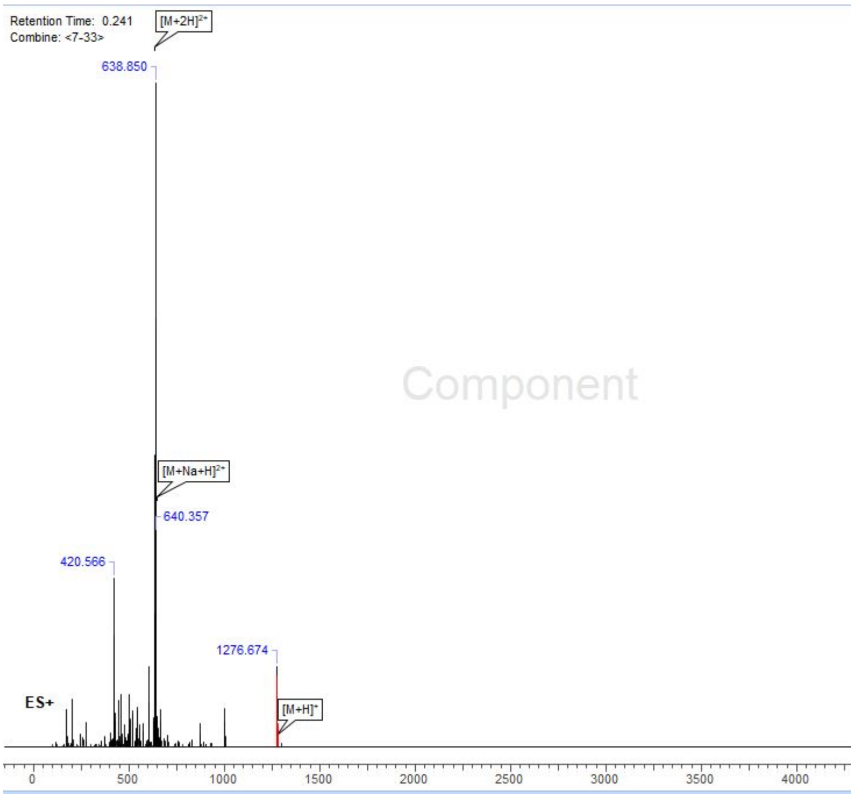

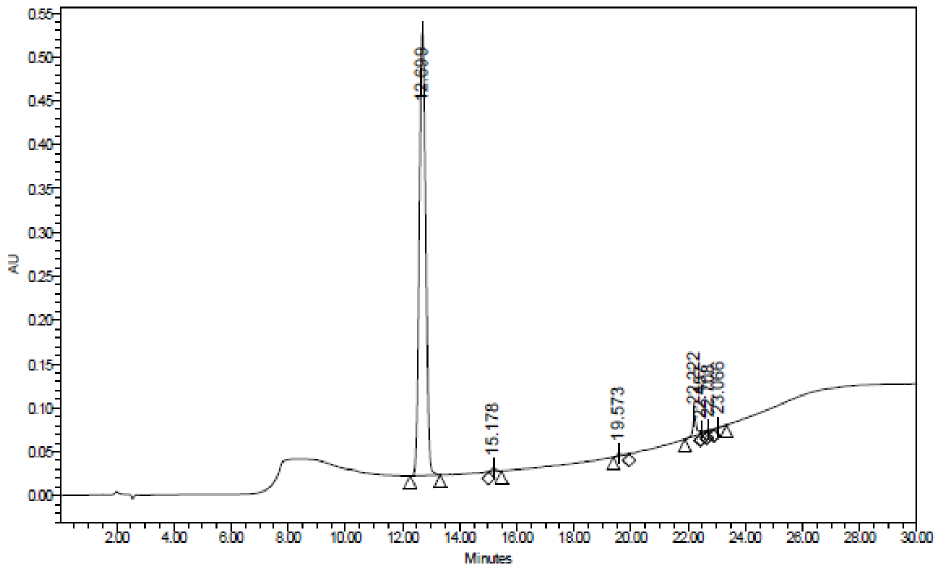

Peak Results

	Name	RT	Area	Height	% Area
1		12.699	7876776	507049	96.10

Compound 18. Charge: +5. t_R = 12.937 min. Gradient: 0-100% B over 20 min. B = 90% MeCN + 0.1% TFA. HRMS: Exact Mass: 1369.4733, Base peak ion: m/z 685.8213 $(M+2H)^{2+}$

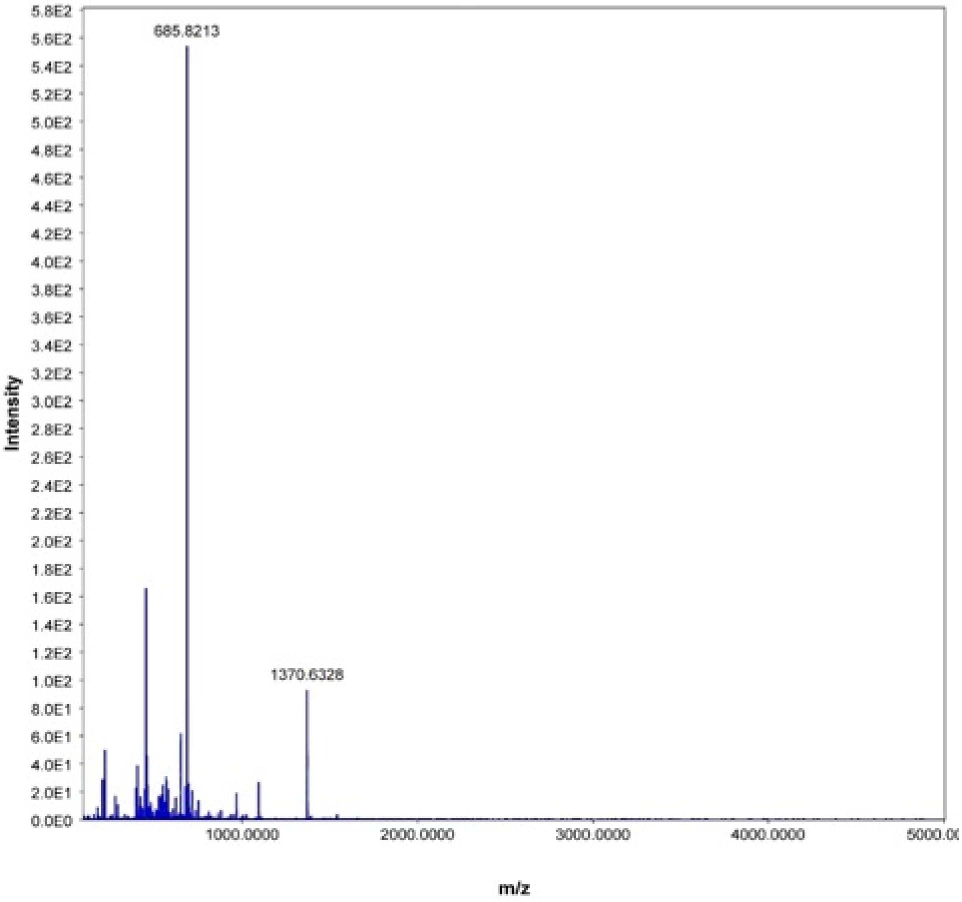

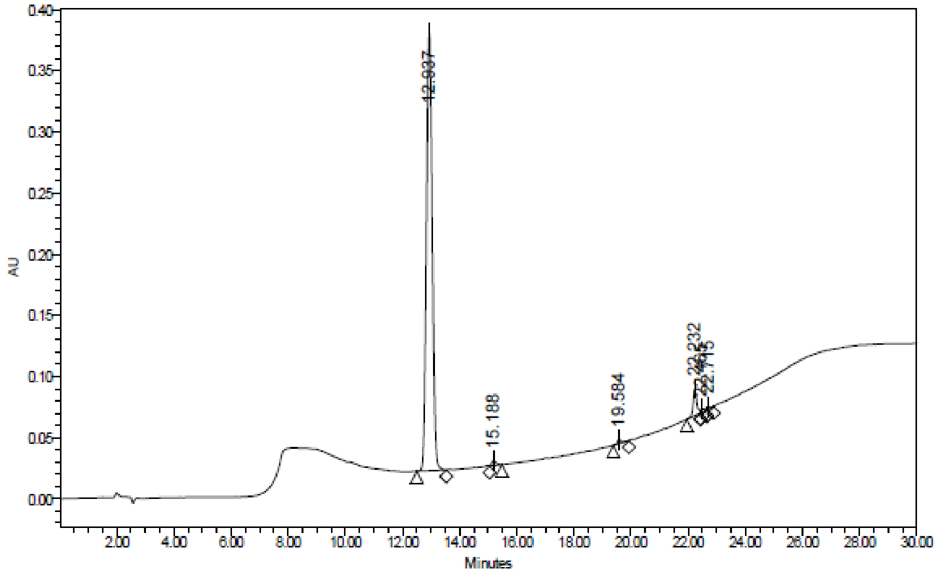

Peak Results

	Name	RT	Area	Height	% Area
1		12.937	5059246	359093	94.26

Compound 19. Charge: +6. t_{R} = 11.838 min. Gradient: 0-100% B over 20 min. B = 90% MeCN + 0.1% TFA. HRMS: Exact Mass: 1392.8434, Base peak ion: m/z 697.5248 $(M+2H)^{2+}$

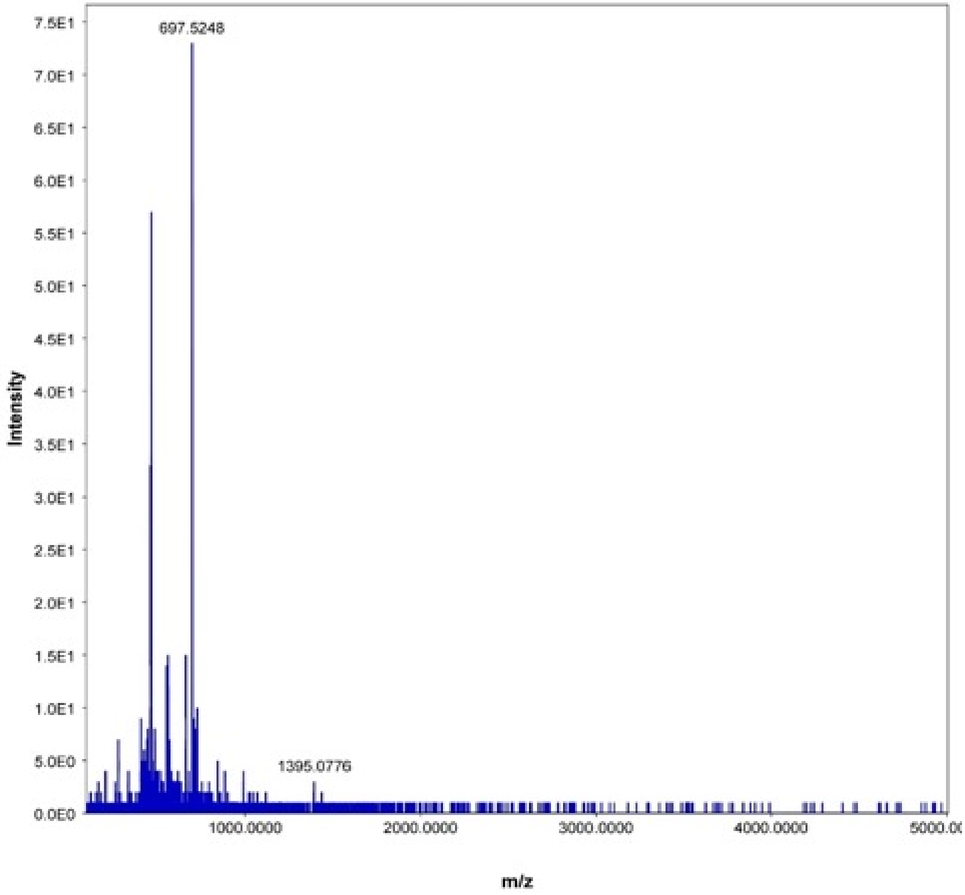

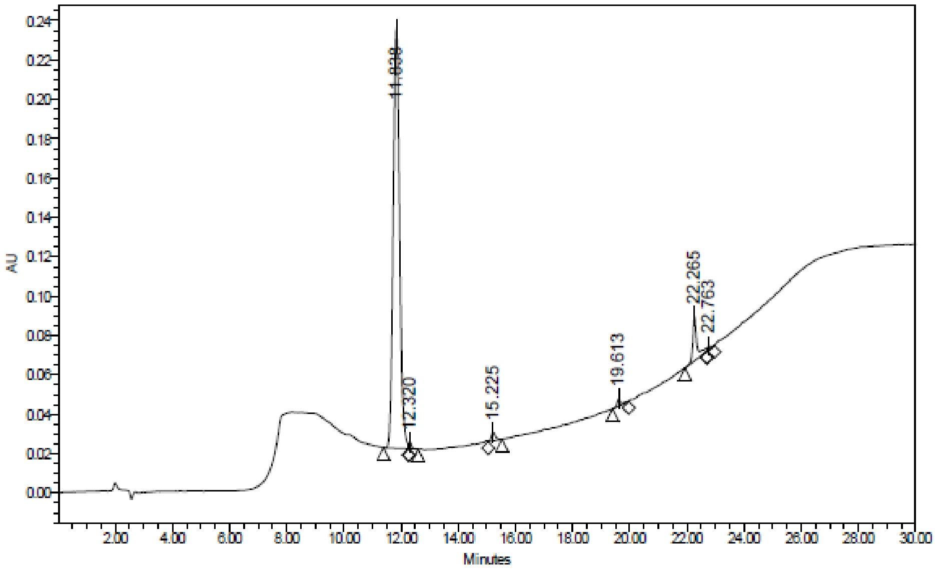

Peak Results

	Name	RT	Area	Height	% Area
1		11.838	3301038	213383	90.63

Compound 20. Charge: +6. t_R = 12.413 min. Gradient: 0-100% B over 20 min. B = 90% MeCN + 0.1% TFA. HRMS: Exact Mass: 1482.7963, Base peak Ion: m/z 742.4987 $(M+2H)^{2+}$

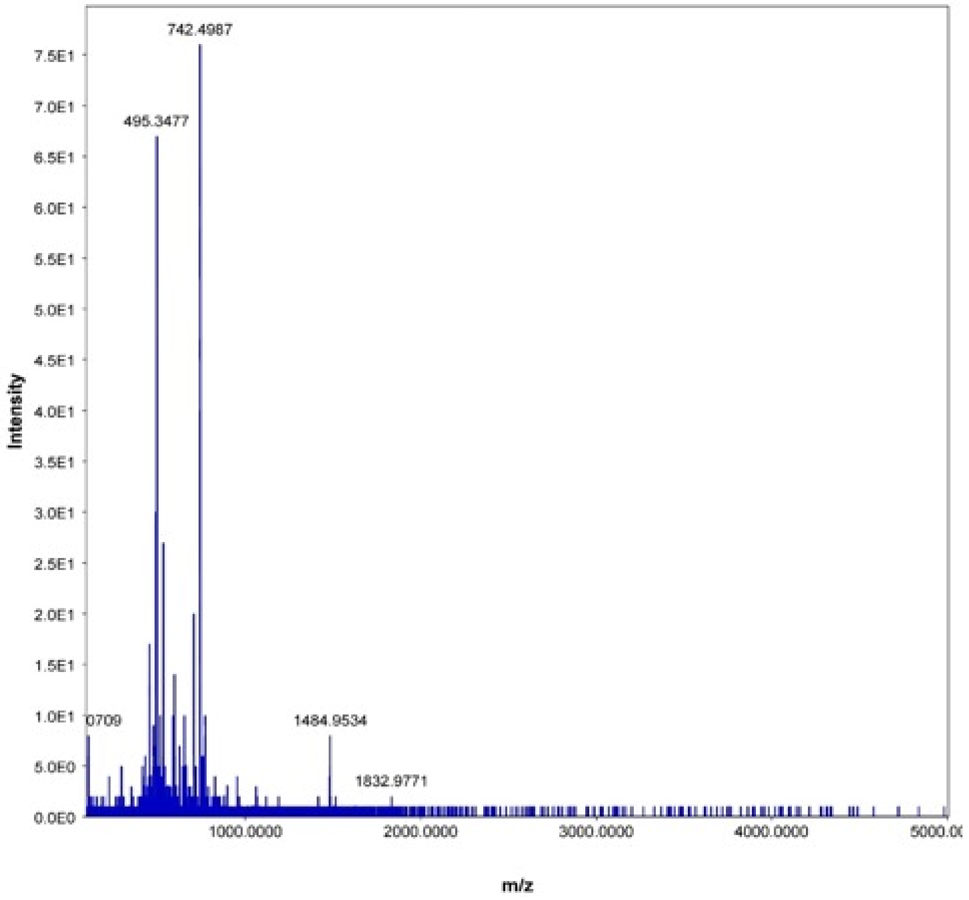

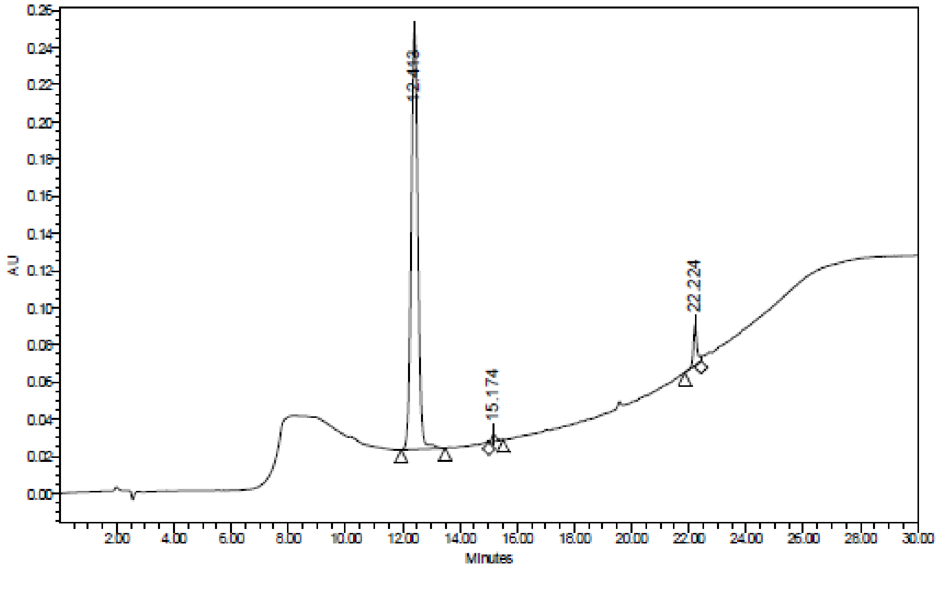

Peak Results

	Name	RT	Area	Height	% Area
1		12.413	3665237	225059	93.98

Compound 21. Charge: +6. t_{R} = 12.961 min. Gradient: 0-100% B over 20 min. B = 90% MeCN + 0.1% TFA. HRMS: Exact Mass: 1562.6486, Base peak ion: m/z 522.643 $(M+3H)^{3+}$

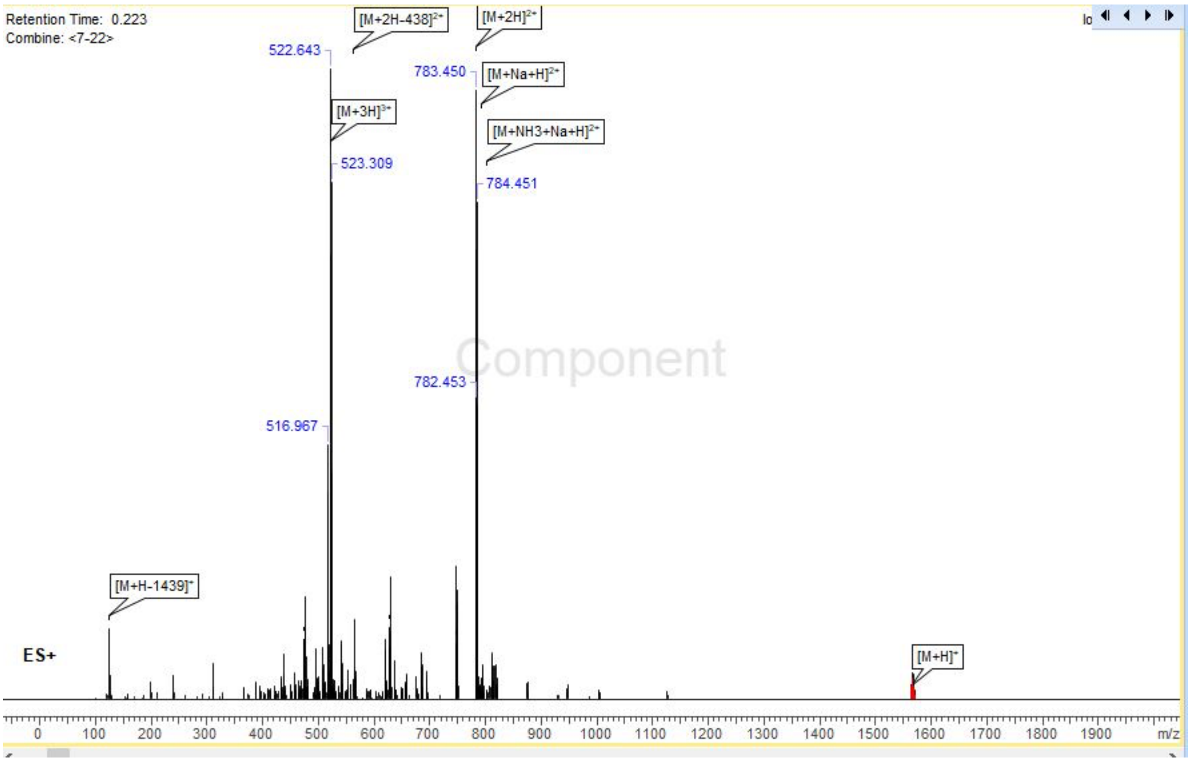

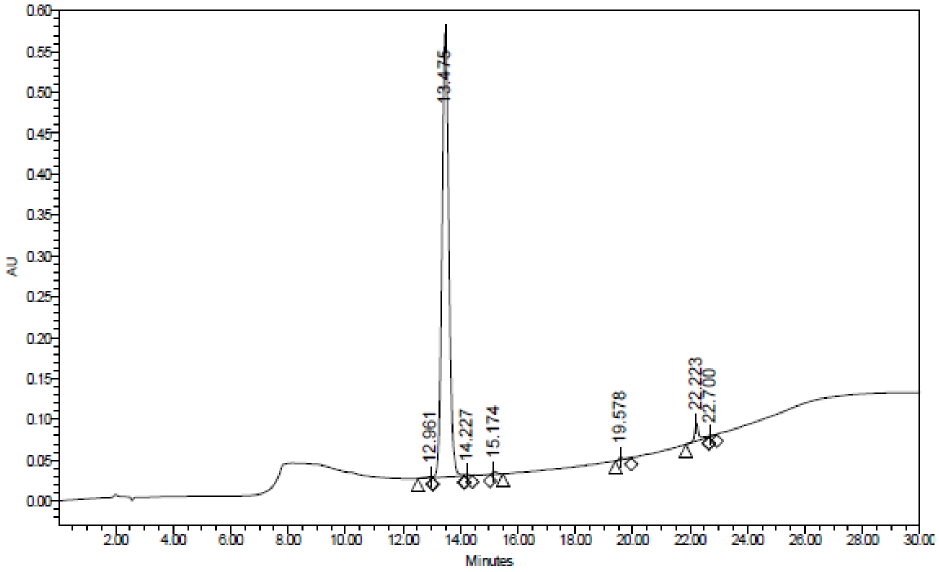

Peak Results

	Name	RT	Area	Height	% Area
1		12.961	29693	1757	0.31

Compound 22. Charge: +6. t_{R} = 13.752 min. Gradient: 0-100% B over 20 min. B = 90% MeCN + 0.1% TFA. HRMS: Exact Mass: 1782.3960, Base peak ion: m/z 597.236 $(M+3H)^{3+}$

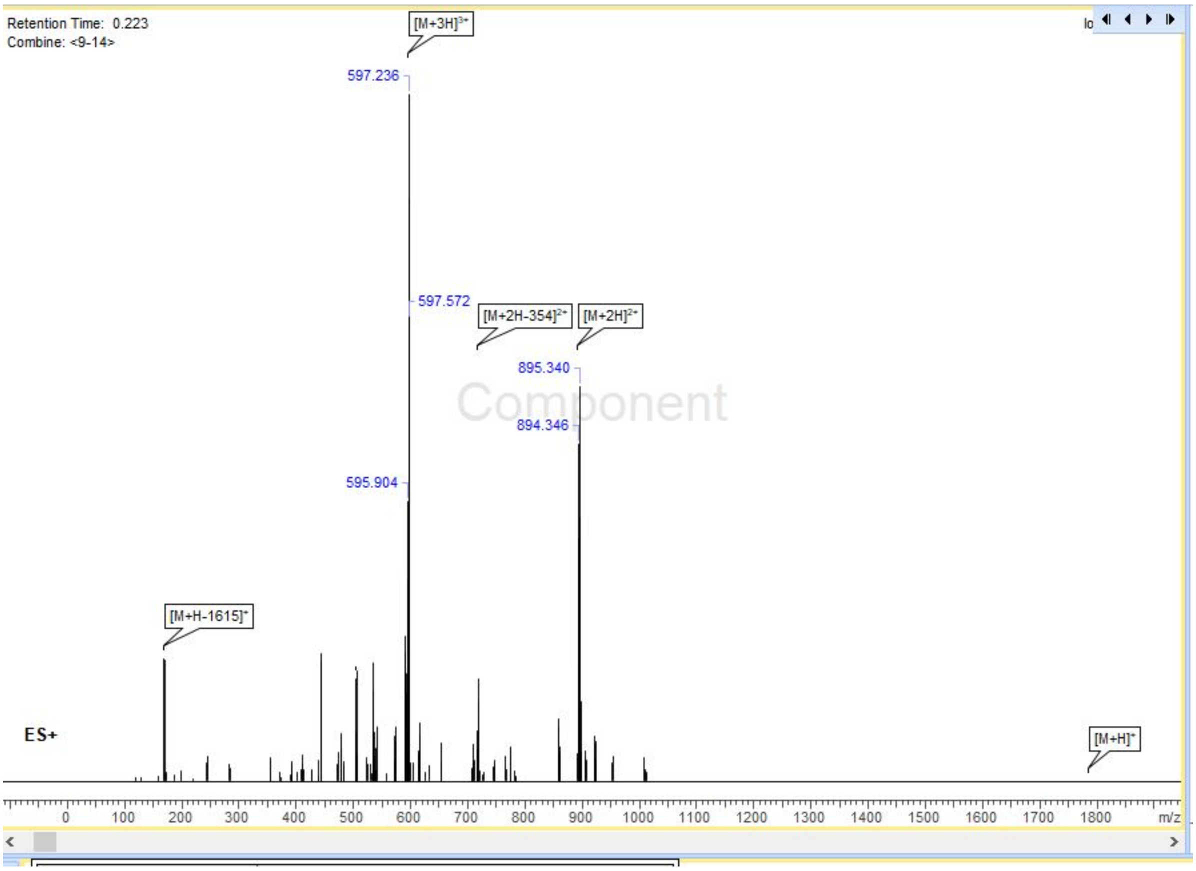

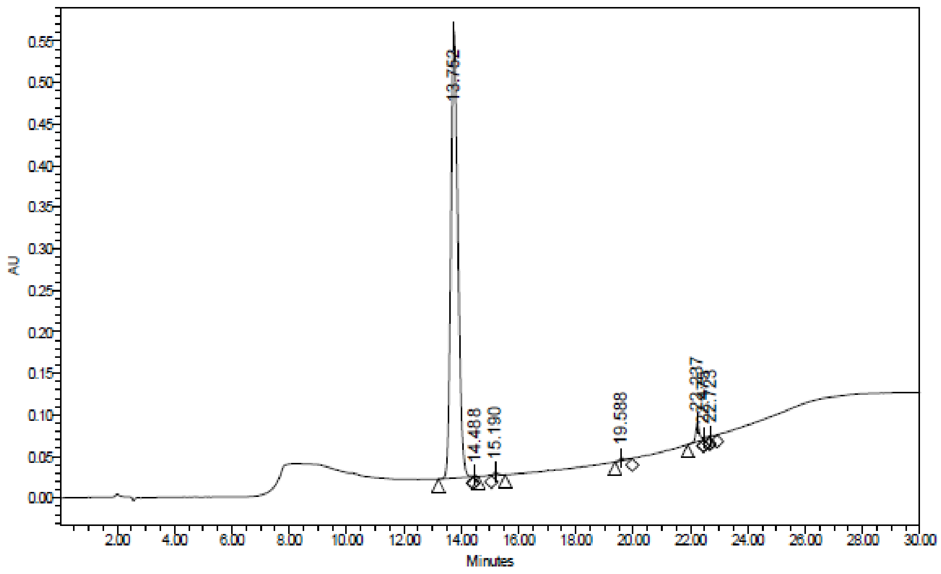

Peak Results

	Name	RT	Area	Height	% Area
1		13.752	9195249	537966	96.58

Compound 23. Charge: +6. t_{R} = 14.088 min. Gradient: 0-100% B over 20 min. B = 90% MeCN + 0.1% TFA. HRMS: Exact Mass: 2022.3267, Base peak ion: m/z 675.2048 $(M+3H)^{3+}$

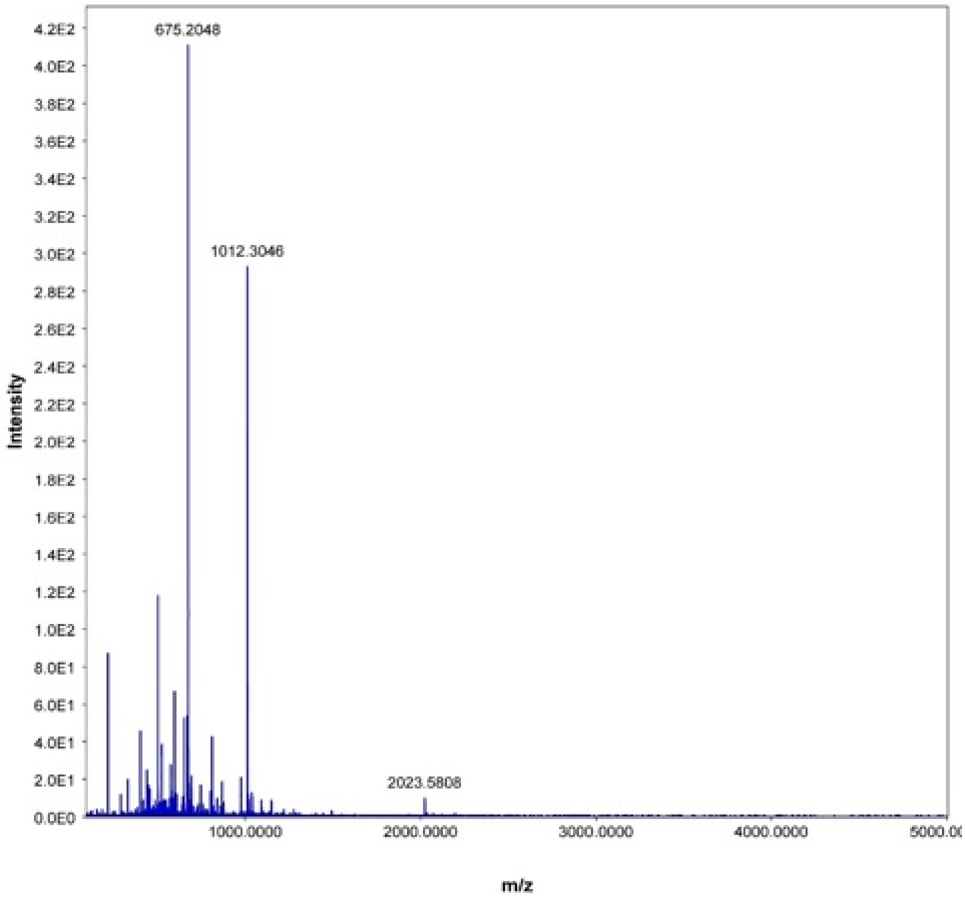

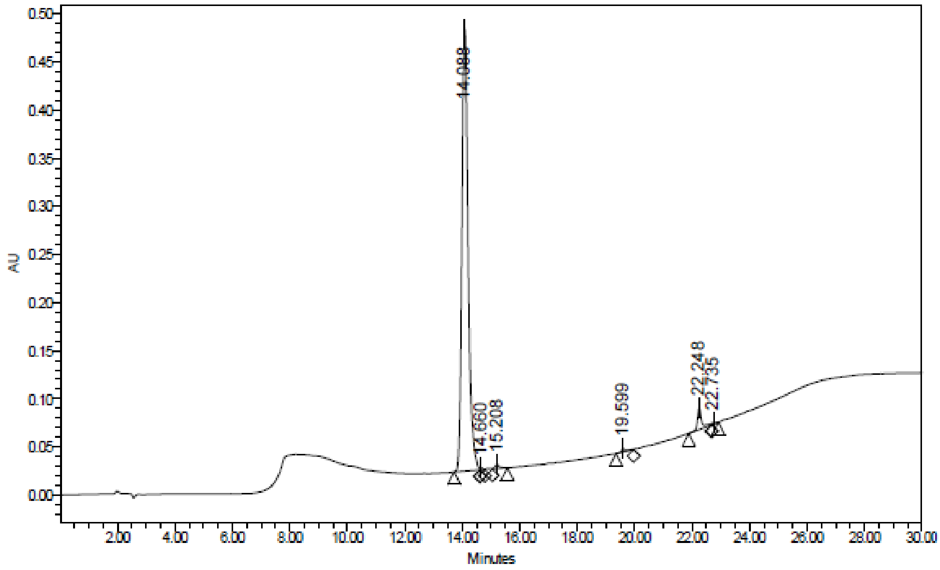

Peak Results

	Name	RT	Area	Height	% Area
1		14.088	6933336	460447	95.57

Compound 24. Charge: +6. t_R = 12.203 min. Gradient: 0-100% B over 20 min. B = 90% MeCN + 0.1% TFA. HRMS: Exact Mass: 1446.8152, Base peak ion: m/z 483.3226 $(M+3H)^{3+}$

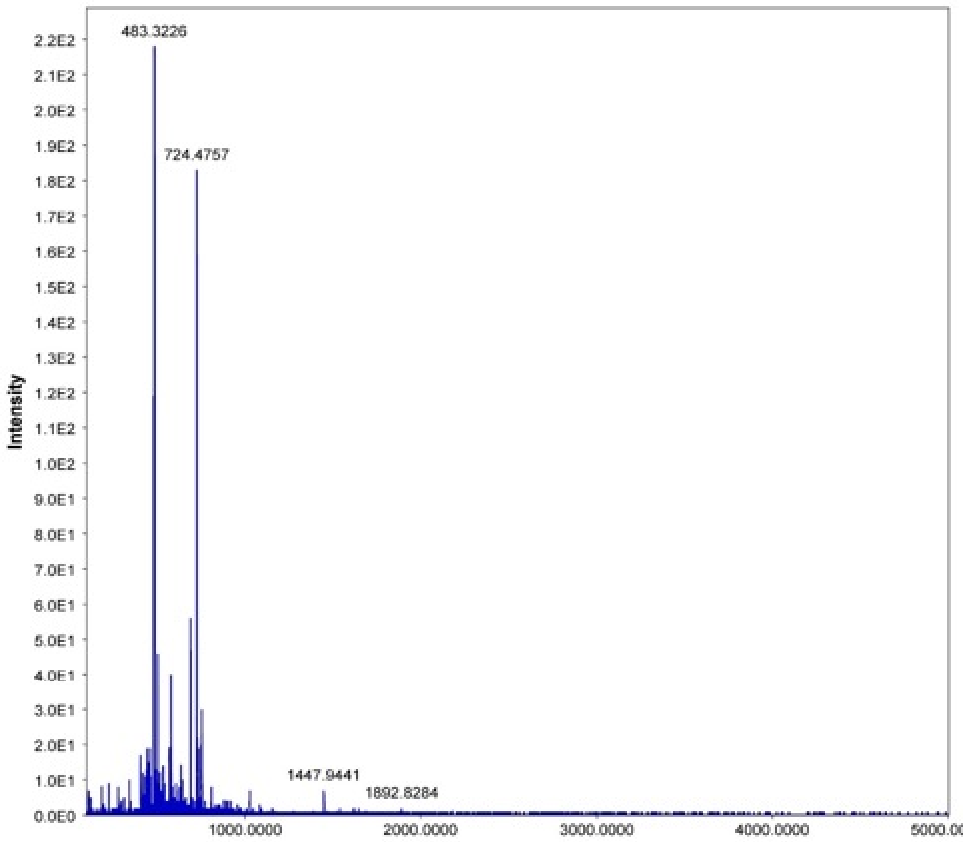

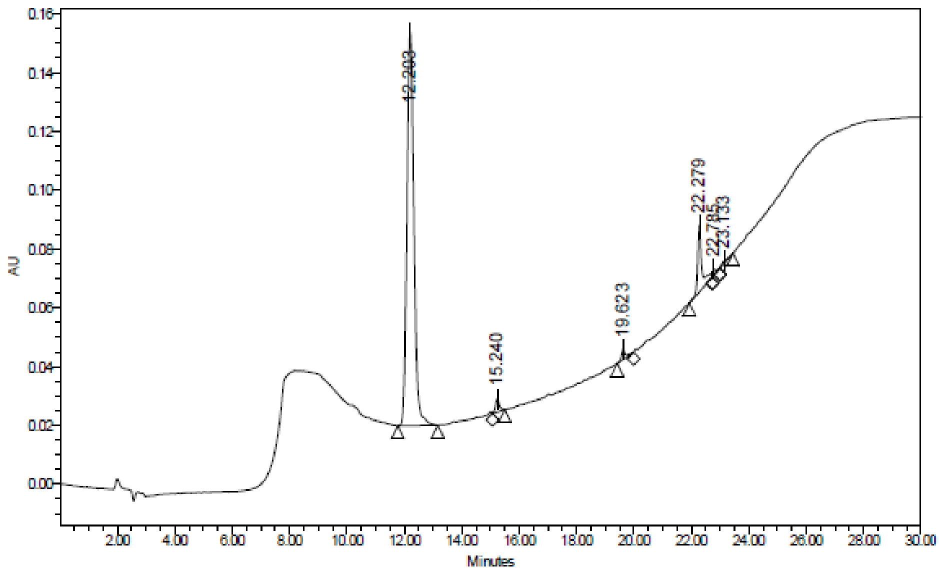

Peak Results

	Name	RT	Area	Height	% Area
1		12.203	2258914	134089	87.02

Compound 25. Charge: +6. t_R = 12.854 min. Gradient: 0-100% B over 20 min. B = 90% MeCN + 0.1% TFA. HRMS: Exact Mass: 1494.7265, Base peak ion: m/z 749.494 $(M+2H)^{2+}$

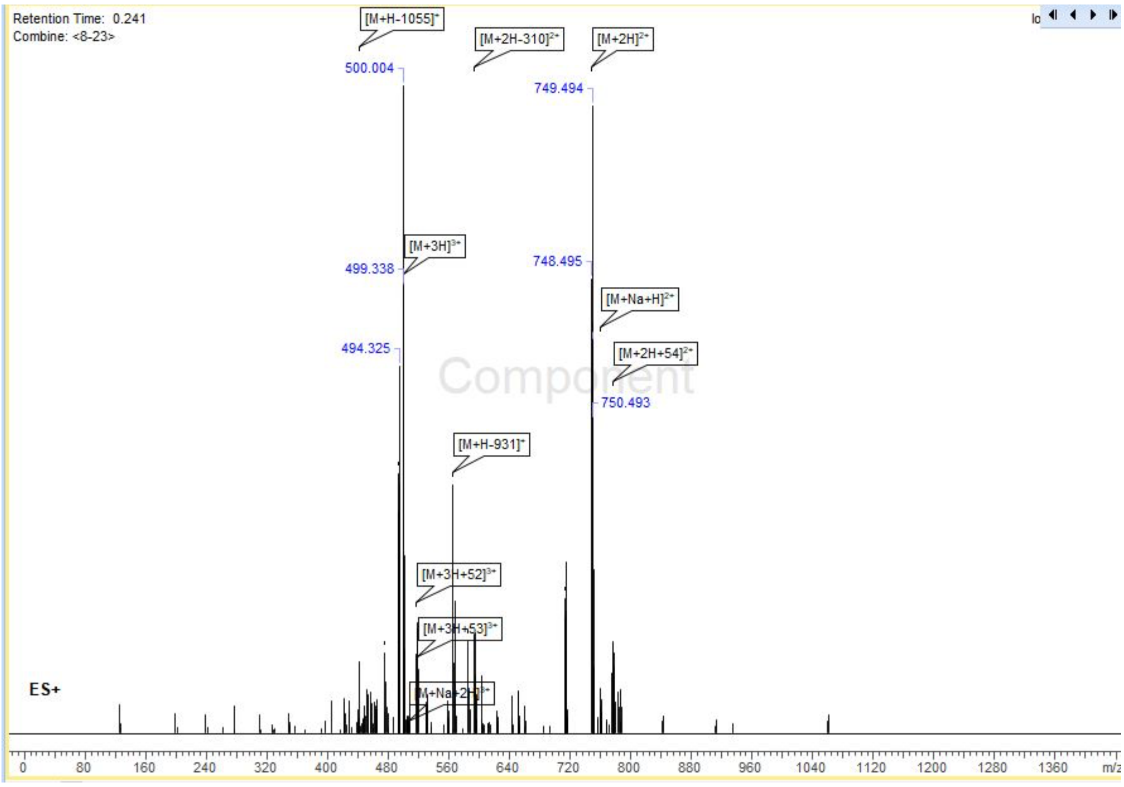

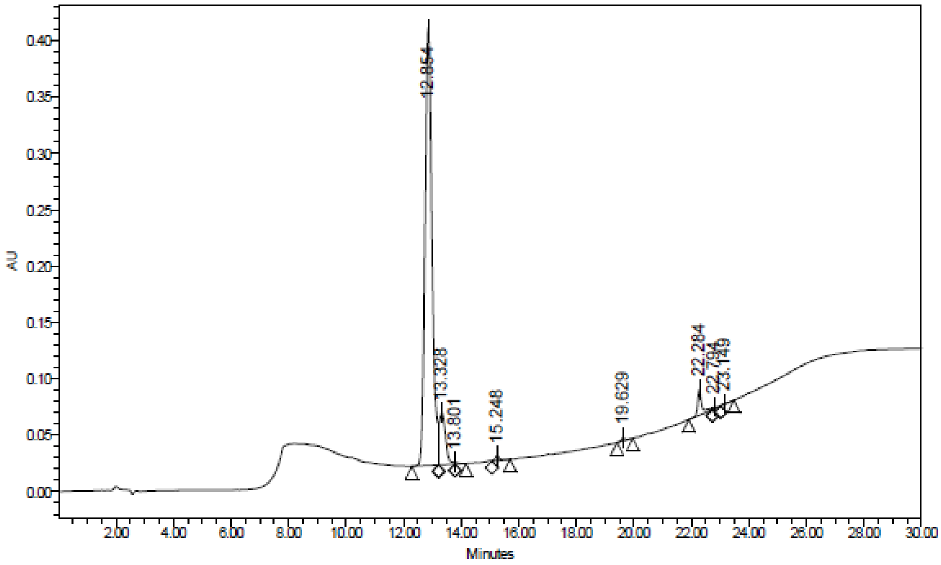

Peak Results

	Name	RT	Area	Height	% Area
1		12.854	6579714	388186	86.35

Compound 26. Charge: +6. t_{R} = 11.879 min. Gradient: 0-100% B over 20 min. B = 90% MeCN + 0.1% TFA. HRMS: Exact Mass: 1626.5750, Base peak ion: m/z 816.426 $(M+2H)^{2+}$

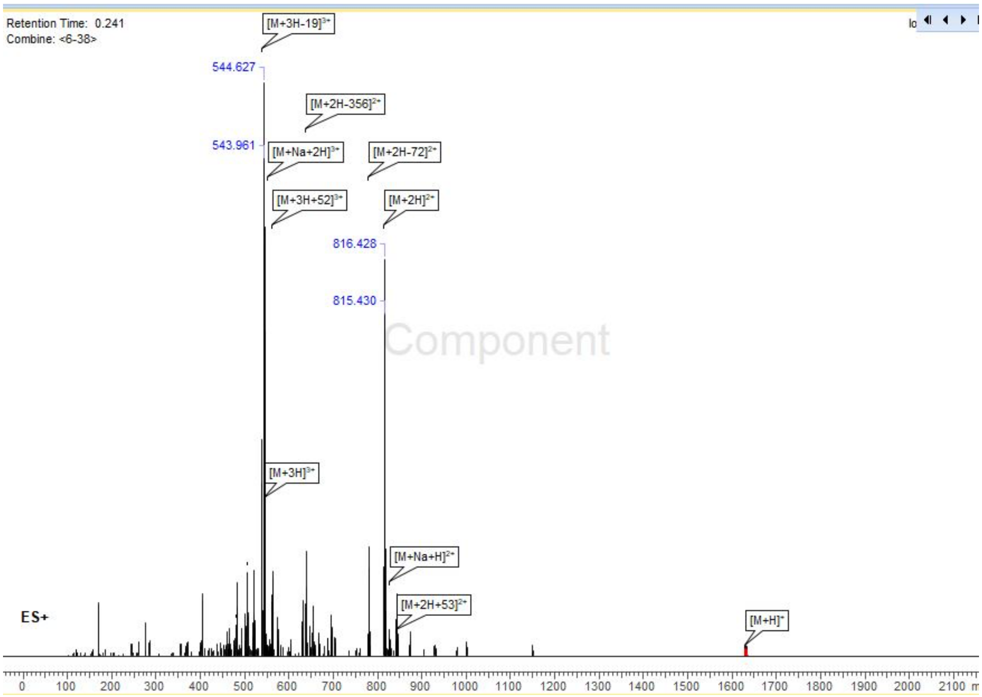

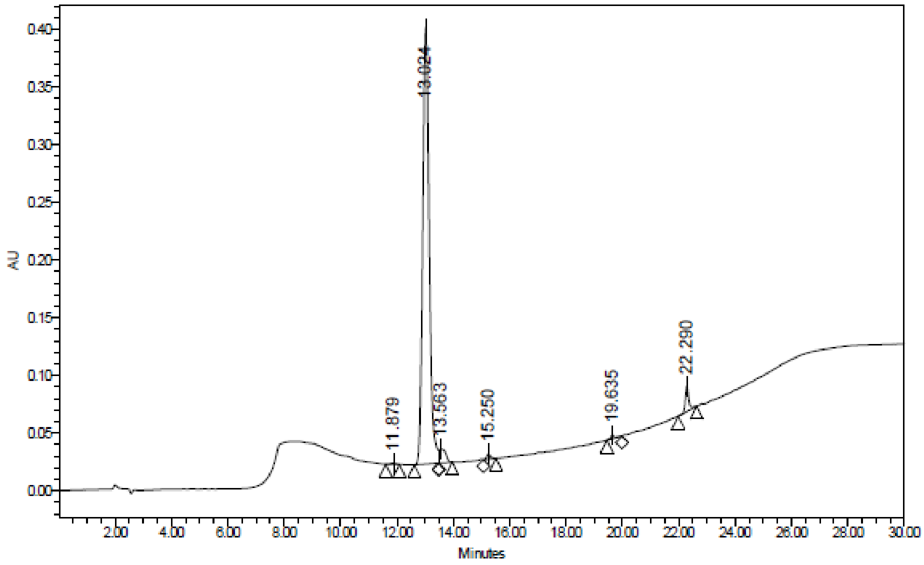

Peak Results

	Name	RT	Area	Height	% Area
1		11.879	15045	1093	0.23

Compound 27. Charge: +6. t_{R} = 13.254 min. Gradient: 0-100% B over 20 min. B = 90% MeCN + 0.1% TFA. HRMS: Exact Mass: 1770.5336, Base peak ion: m/z 591.2683 $(M+3H)^{3+}$

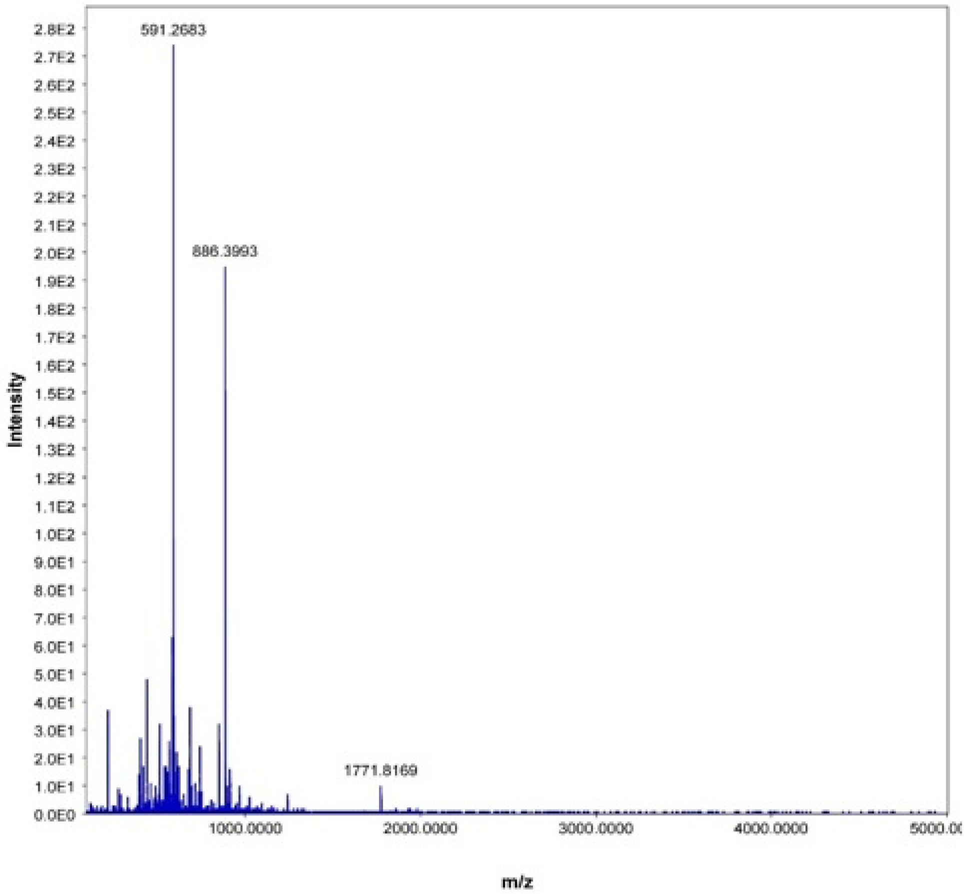

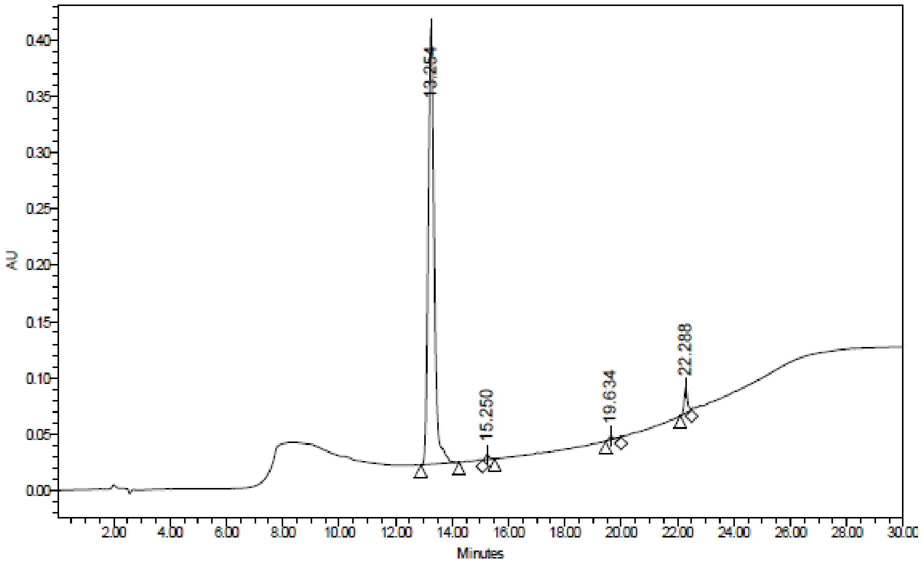

Peak Results

	Name	RT	Area	Height	% Area
1		13.254	5768036	387400	95.78

Compound 28. Charge: +7. t_{R} = 11.928 min. Gradient: 0-100% B over 20 min. B = 90% MeCN + 0.1% TFA. HRMS: Exact Mass: 1668.0068, Base peak ion: m/z 557.4275 $(M+3H)^{3+}$

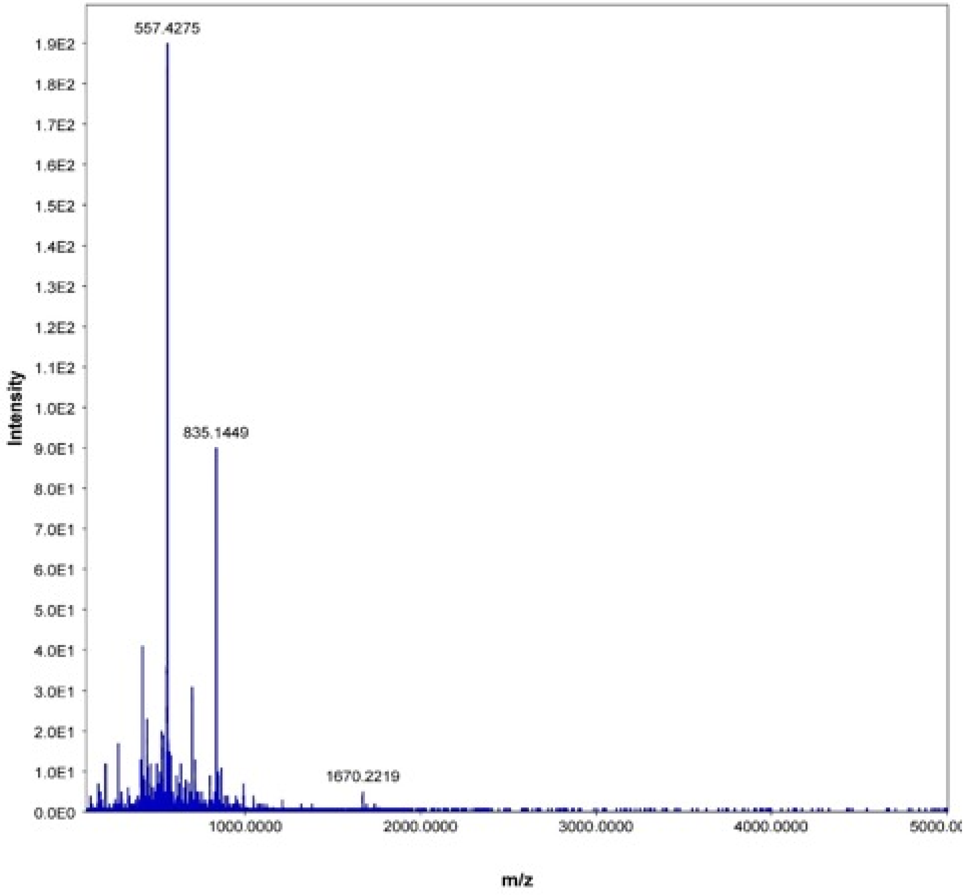

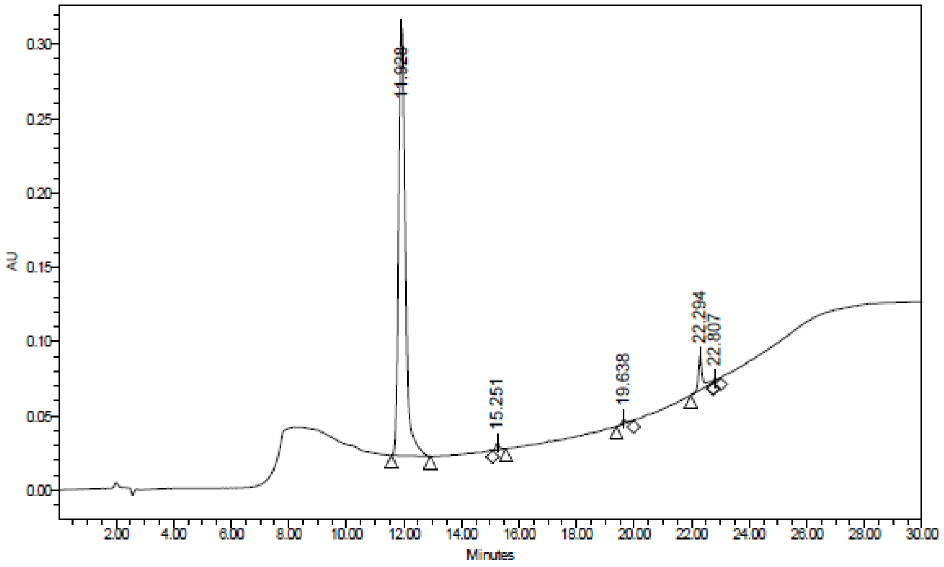

Peak Results

	Name	RT	Area	Height	% Area
1		11.928	4719187	287870	93.71

Compound 29. Charge: +7. t_{R} = 12.522 min. Gradient: 0-100% B over 20 min. B = 90% MeCN + 0.1% TFA. HRMS: Exact Mass: 1775.9503, Base peak ion: m/z 593.092 $(M+3H)^{3+}$

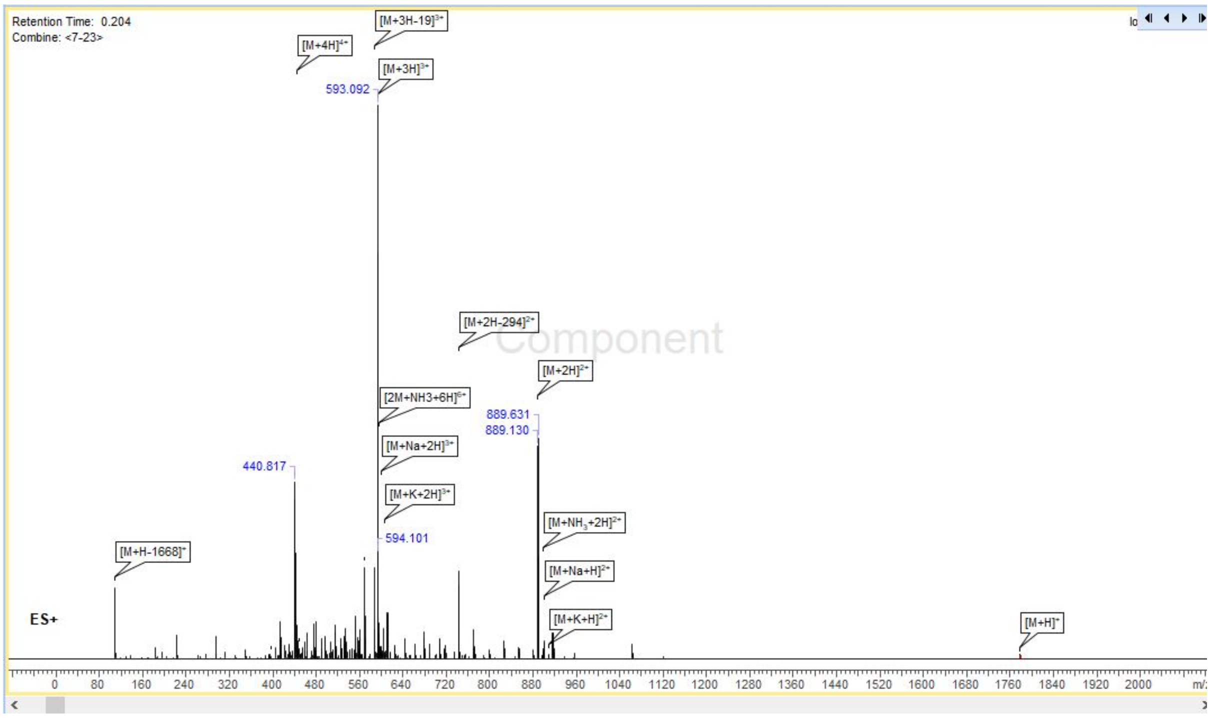

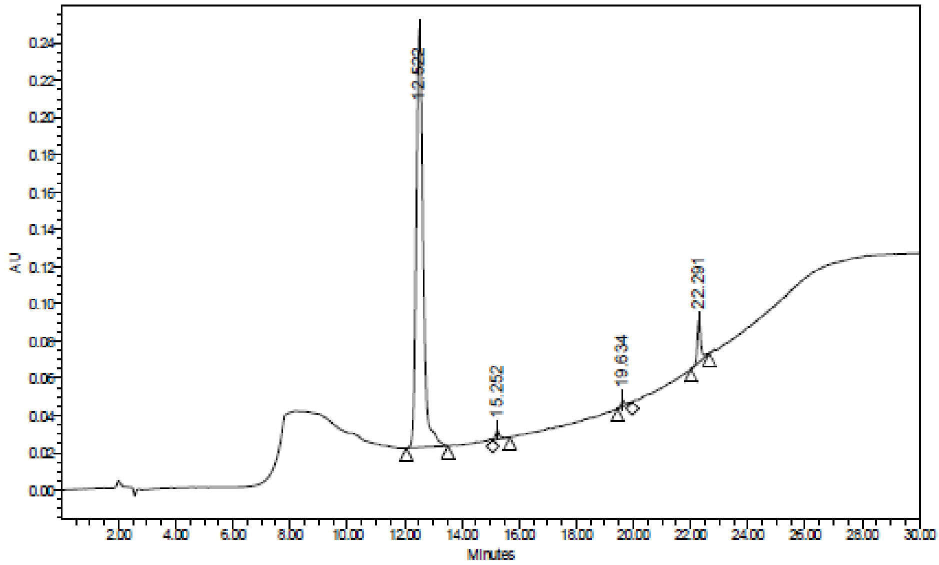

Peak Results

	Name	RT	Area	Height	% Area
1		12.522	3755050	224675	93.38

Compound 30. Charge: +7. t_R = 13.572 min. Gradient: 0-100% B over 20 min. B = 90% MeCN + 0.1% TFA. HRMS: Exact Mass: 1871.7730, Base peak ion: m/z 625.377 $(M+3H)^{3+}$

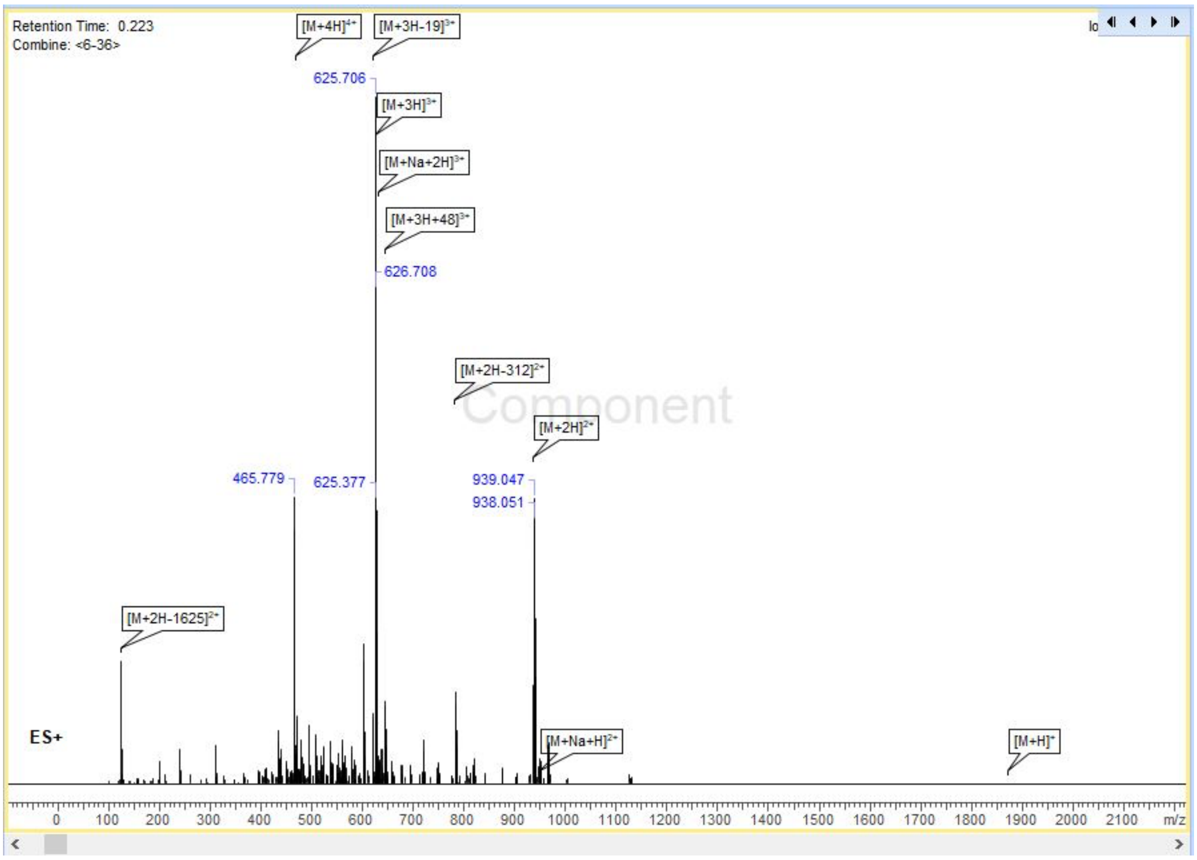

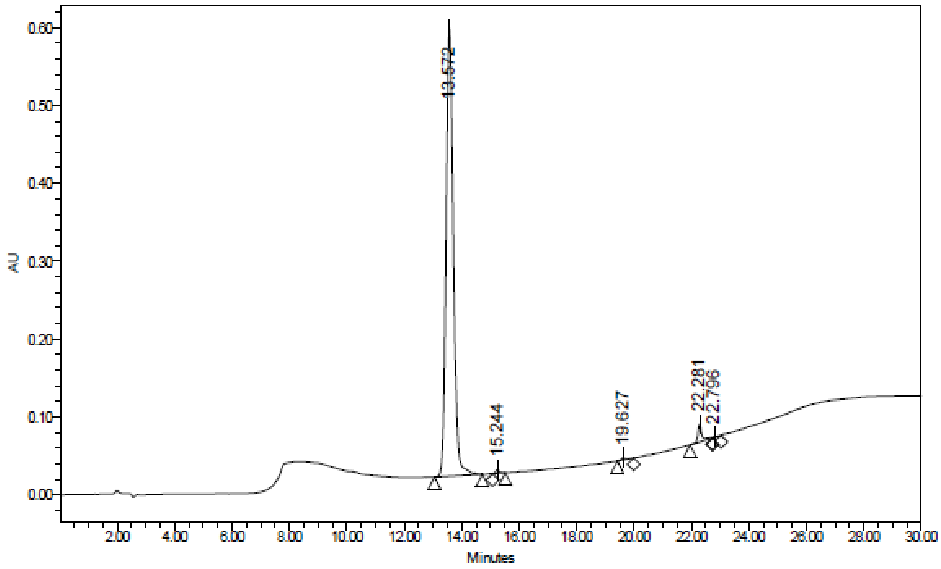

Peak Results

	Name	RT	Area	Height	% Area
1		13.572	10235886	574771	96.97

Compound 31. Charge: +7. t_R = 13.850 min. Gradient: 0-100% B over 20 min. B = 90% MeCN + 0.1% TFA. HRMS: Exact Mass: 2142.5190, Base peak ion: m/z 715.5967 $(M+3H)^{3+}$

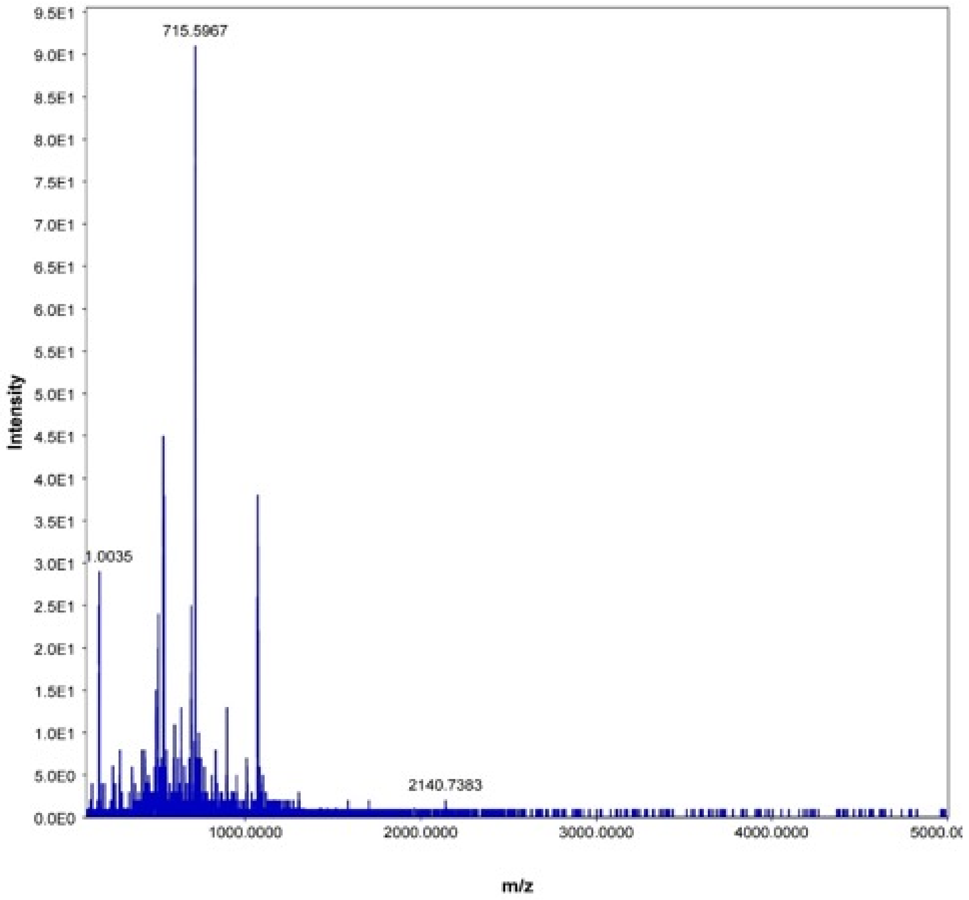

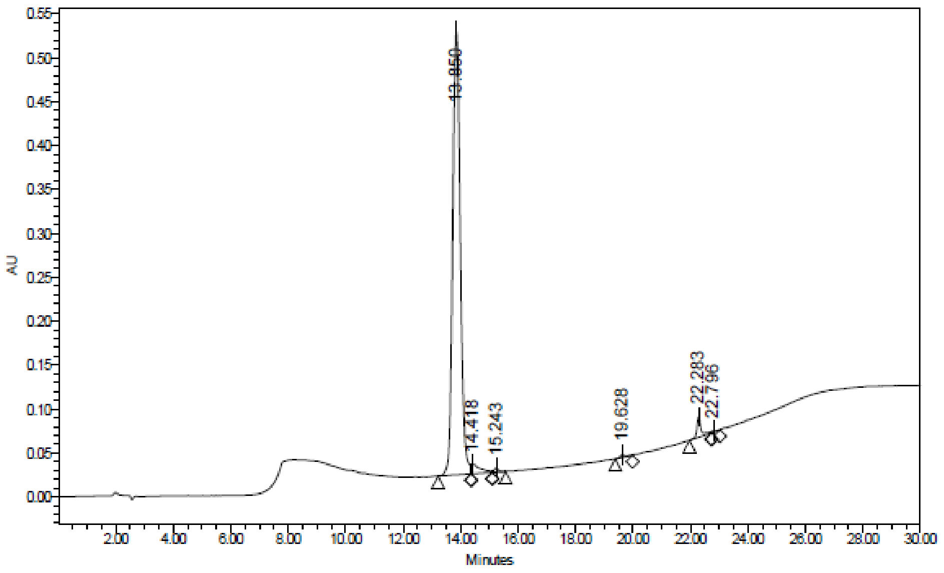

Peak Results

	Name	RT	Area	Height	% Area
1		13.850	9205728	504824	94.26

Compound 32. Charge: +7. $t_{R\,=\,.}$ Gradient: 0-100% B over 20 min. B = 90% MeCN + 0.1% TFA. HRMS: Exact Mass: 2423.3867, Base peak ion: m/z 808.9290 $(M+3H)^{3+}$

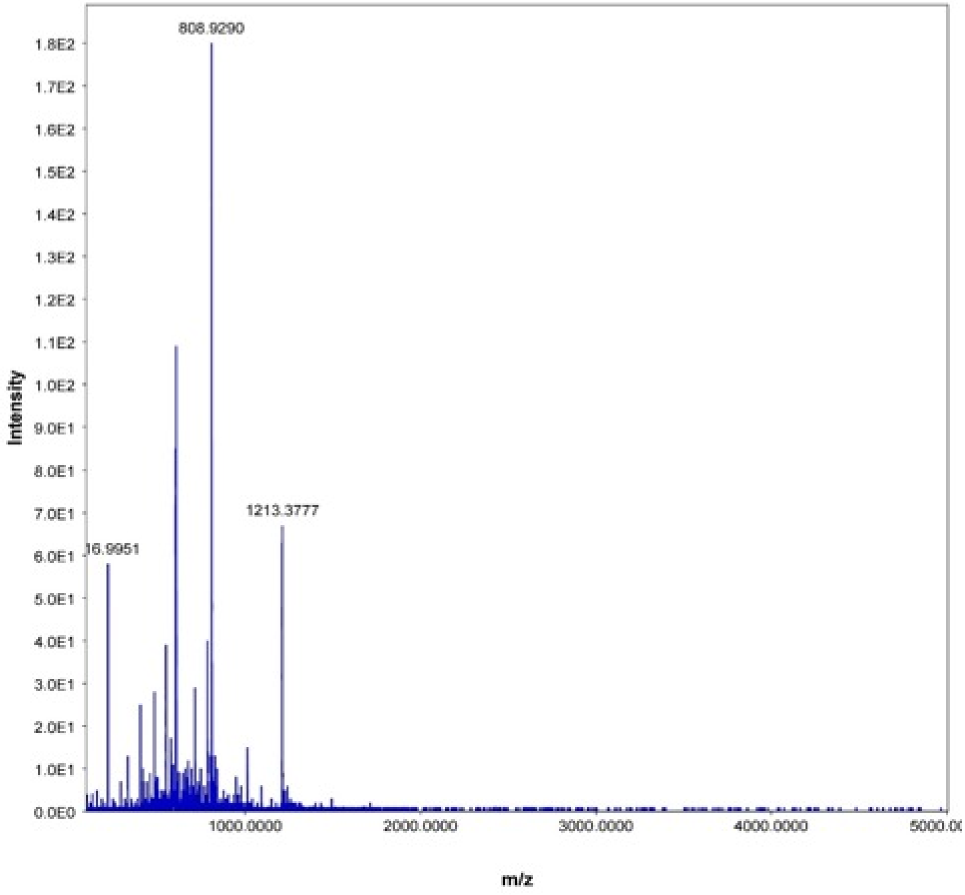

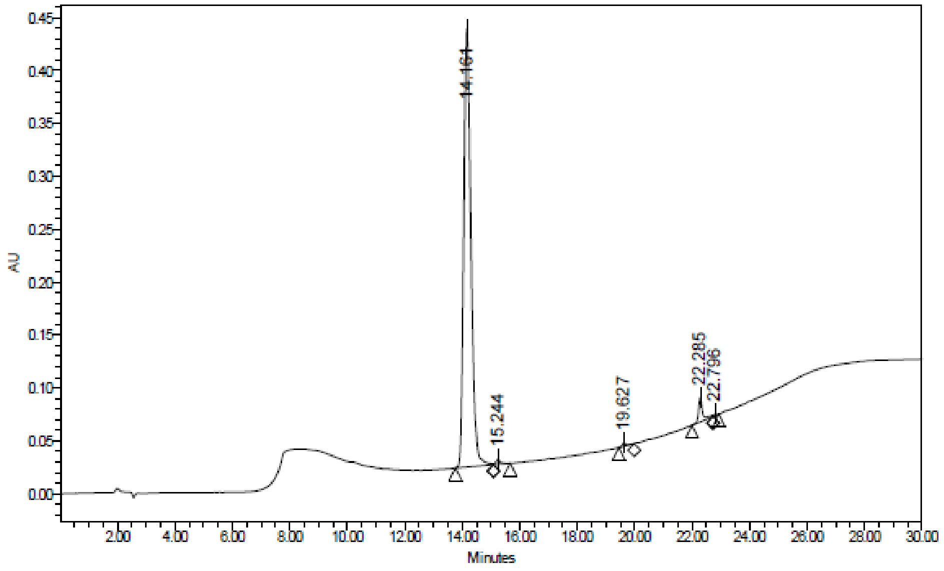

Peak Results

	Name	RT	Area	Height	% Area
1		14.161	7226255	414963	95.80

Compound 33. Charge: +7. t_R = 12.246 min. Gradient: 0-100% B over 20 min. B = 90% MeCN + 0.1% TFA. HRMS: Exact Mass: 1721.9786, Base peak ion: m/z 575.0908 $(M+3H)^{3+}$

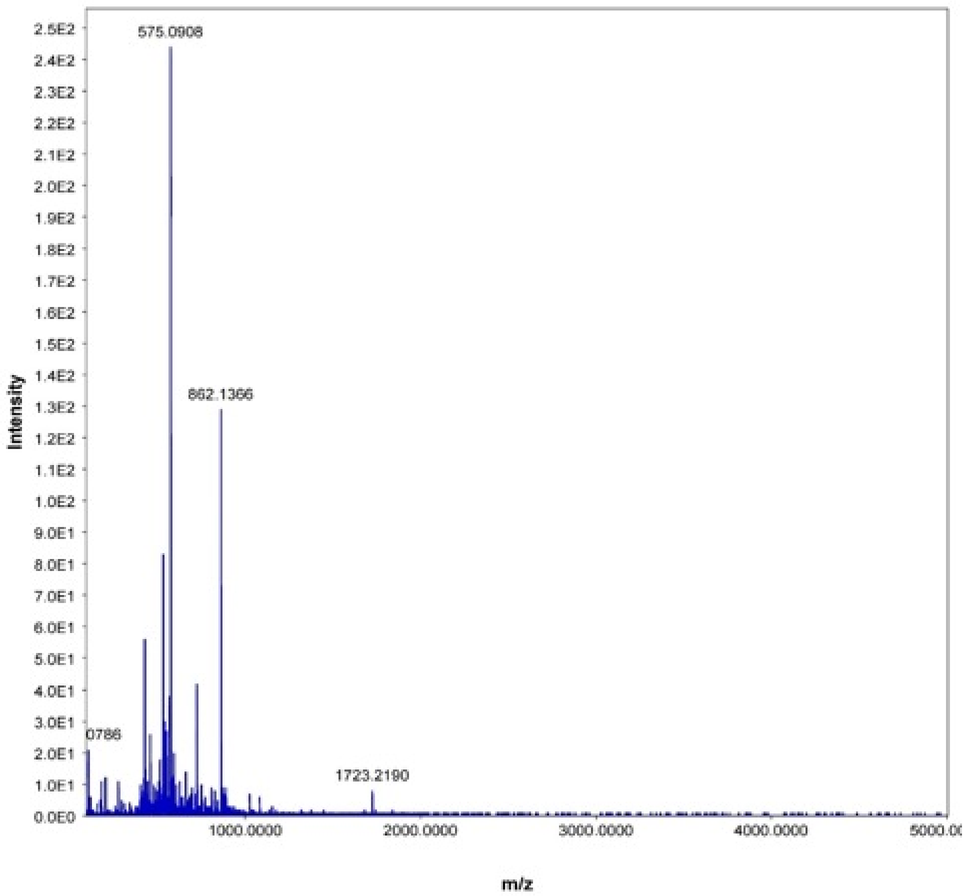

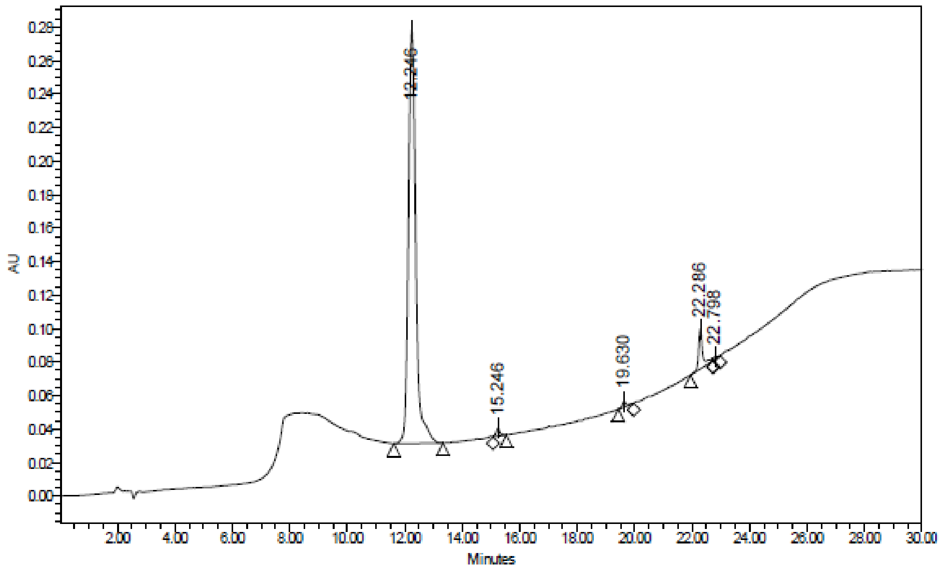

Peak Results

	Name	RT	Area	Height	% Area
1		12.246	4432659	247268	93.12

Compound 34. Charge: +7. Gradient: 0-100% B over 20 min. B = 90% MeCN + 0.1% TFA. HRMS: Exact Mass: 1769.8899, Base peak ion: m/z 591.076 $(M+3H)^{3+}$

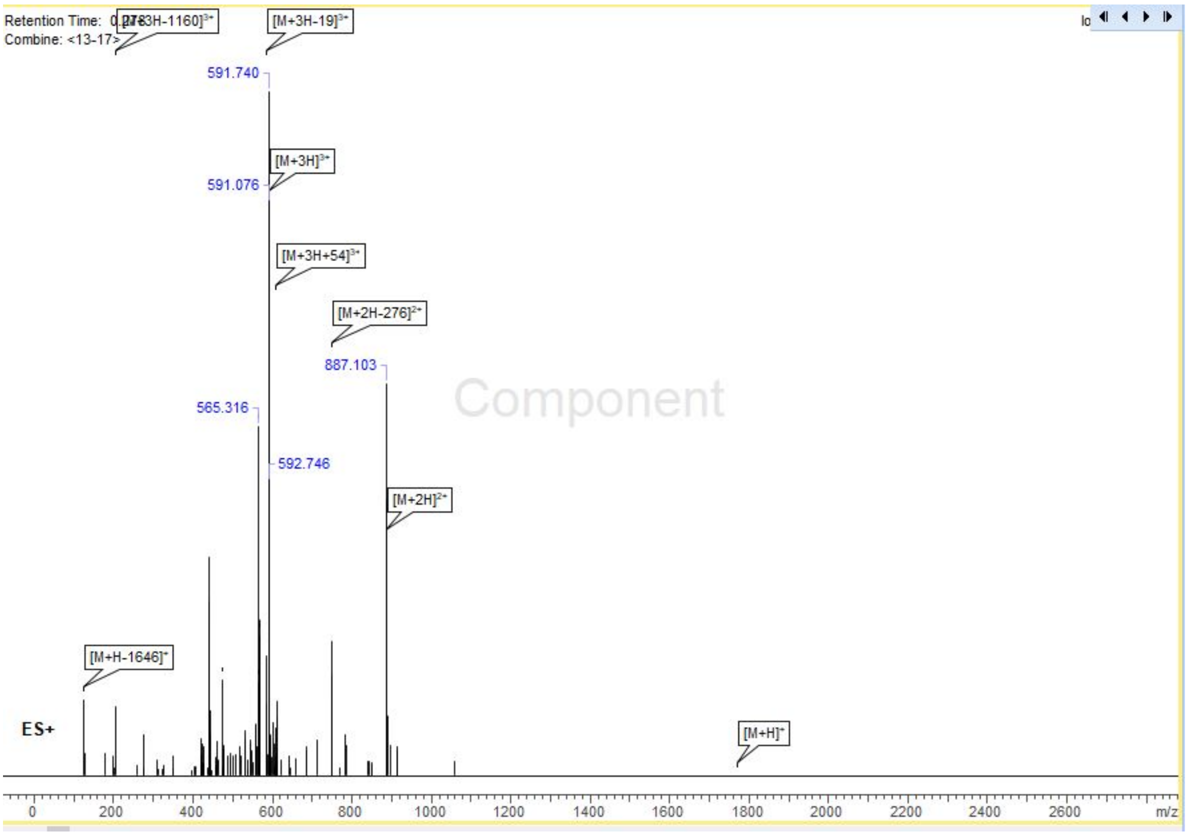

Compound 35. Charge: +7. t_R = 12.963 min. Gradient: 0-100% B over 20 min. B = 90% MeCN + 0.1% TFA. HRMS: Exact Mass: 1901.7384, Base peak ion: 636.3633 $(M+3H)^{3+}$

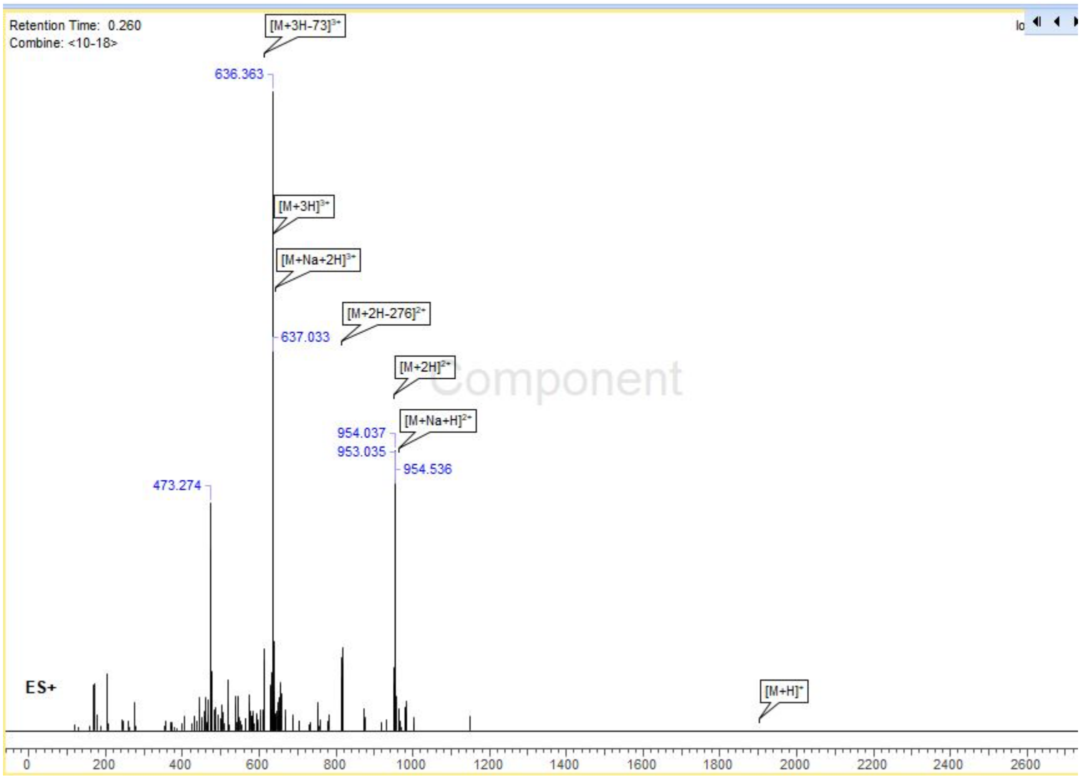

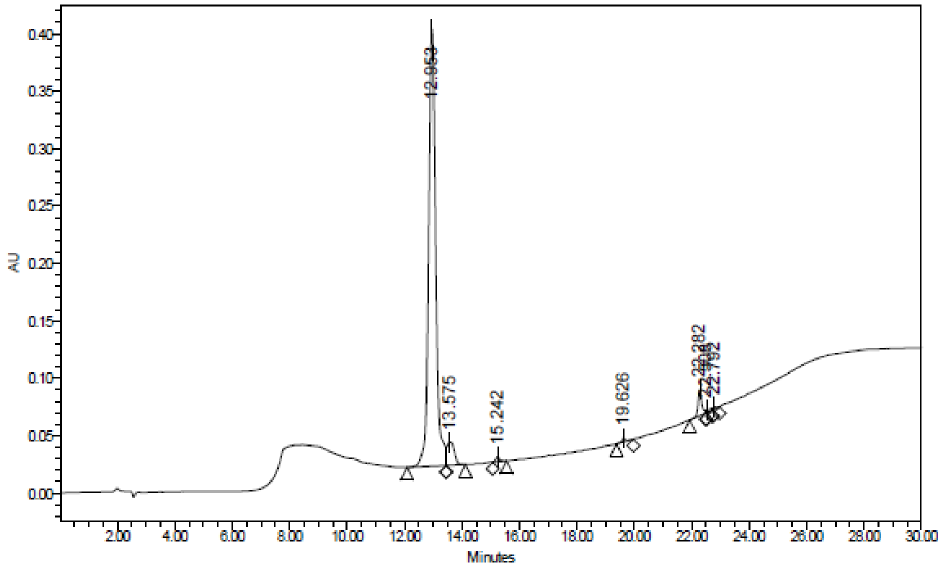

Peak Results

	Name	RT	Area	Height	% Area
1		12.963	6537822	381140	90.61

Compound 36. Charge: +7. t_R = 13.160 min. Gradient: 0-100% B over 20 min. B = 90% MeCN + 0.1% TFA. HRMS: Exact Mass: 2045.6967, Base peak ion: m/z 683.021 $(M+3H)^{3+}$

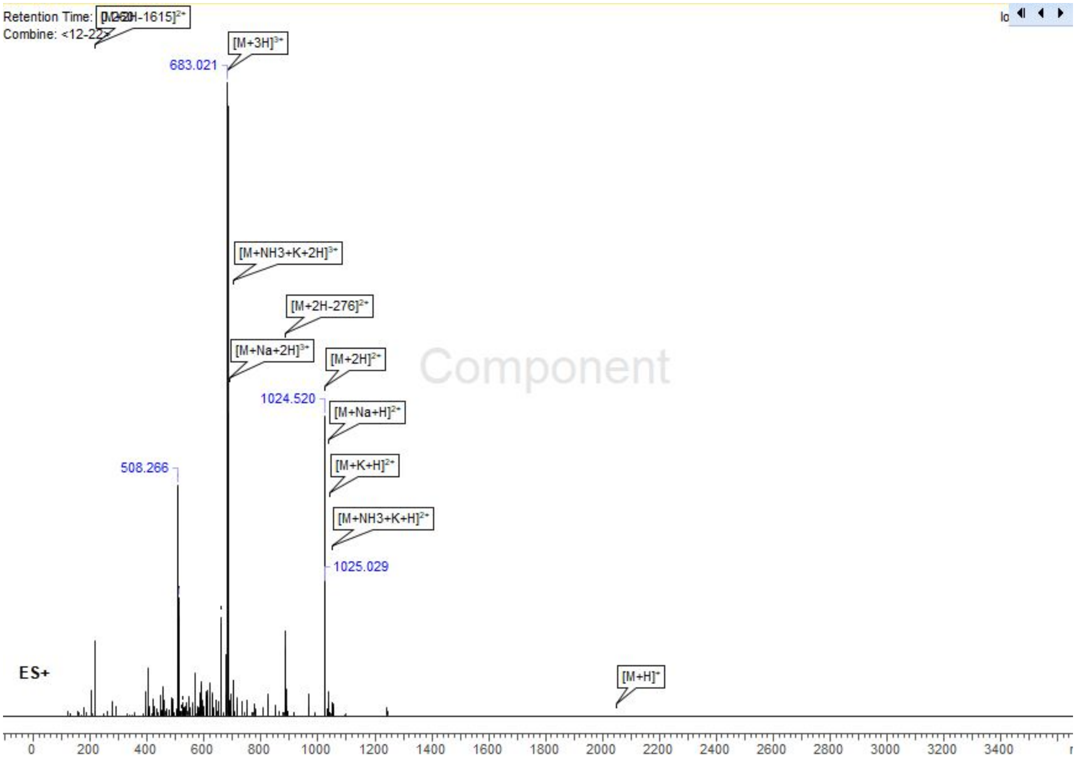

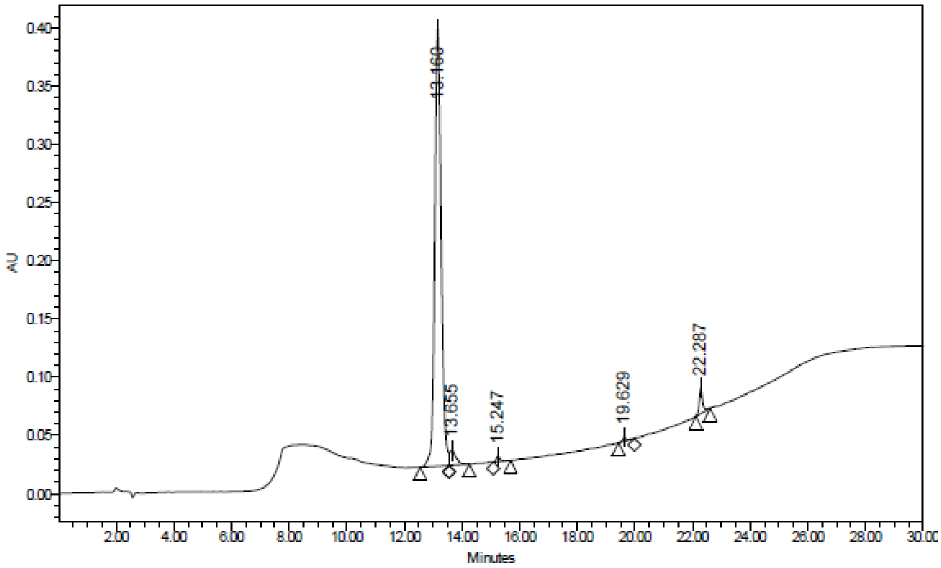

Peak Results

	Name	RT	Area	Height	% Area
1		13.160	5847500	376742	92.67

Compound 37. Charge: +5. t_R = 18.251 min. Gradient: 0-100% B over 20 min. B = 90% MeCN + 0.1% TFA. HRMS: Exact Mass 2089.7671, Base peak ion: m/z 699.047 $(M+3H)^{3+}$

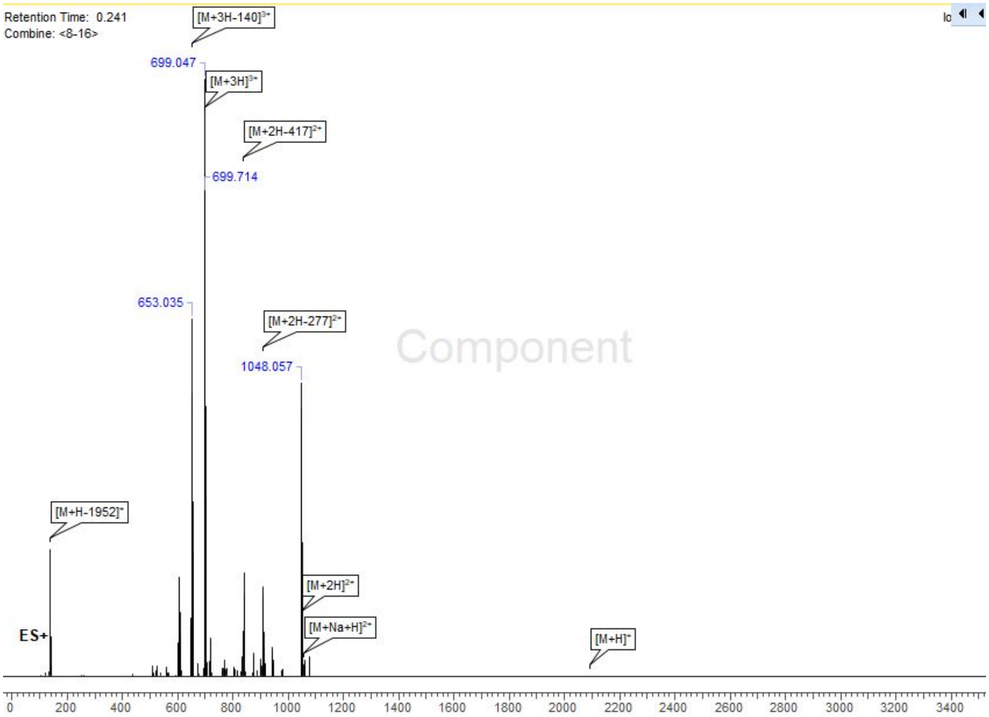

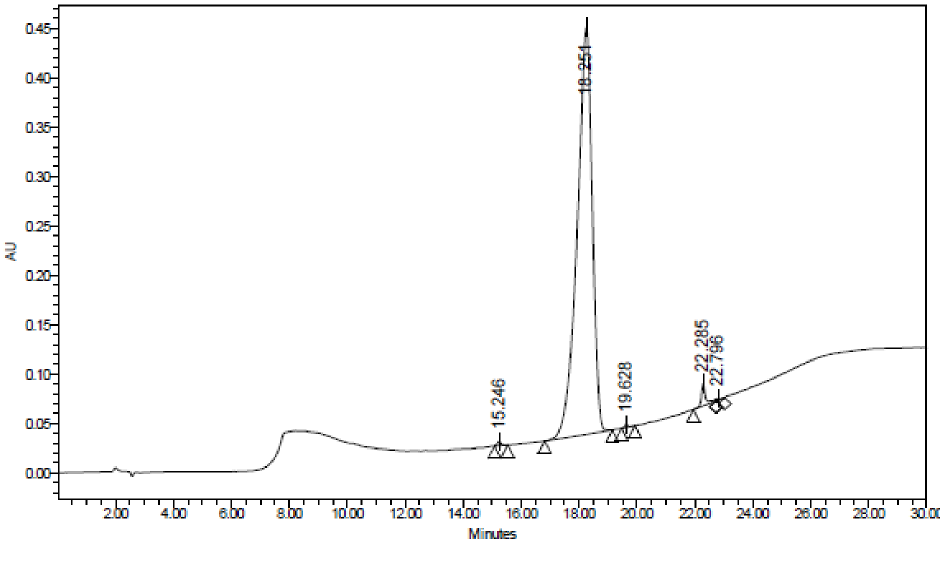

Peak Results

	Name	RT	Area	Height	% Area
1		15.246	38837	4401	0.26
2		18.251	14899909	411775	97.97

Compound 38. Charge: +5. t_R = 16.800 min. Gradient: 0-100% B over 20 min. B = 90% MeCN + 0.1% TFA. HRMS: Exact Mass: 1953.9230, Base peak ion: m/z 653.0874 $(M+3H)^{3+}$

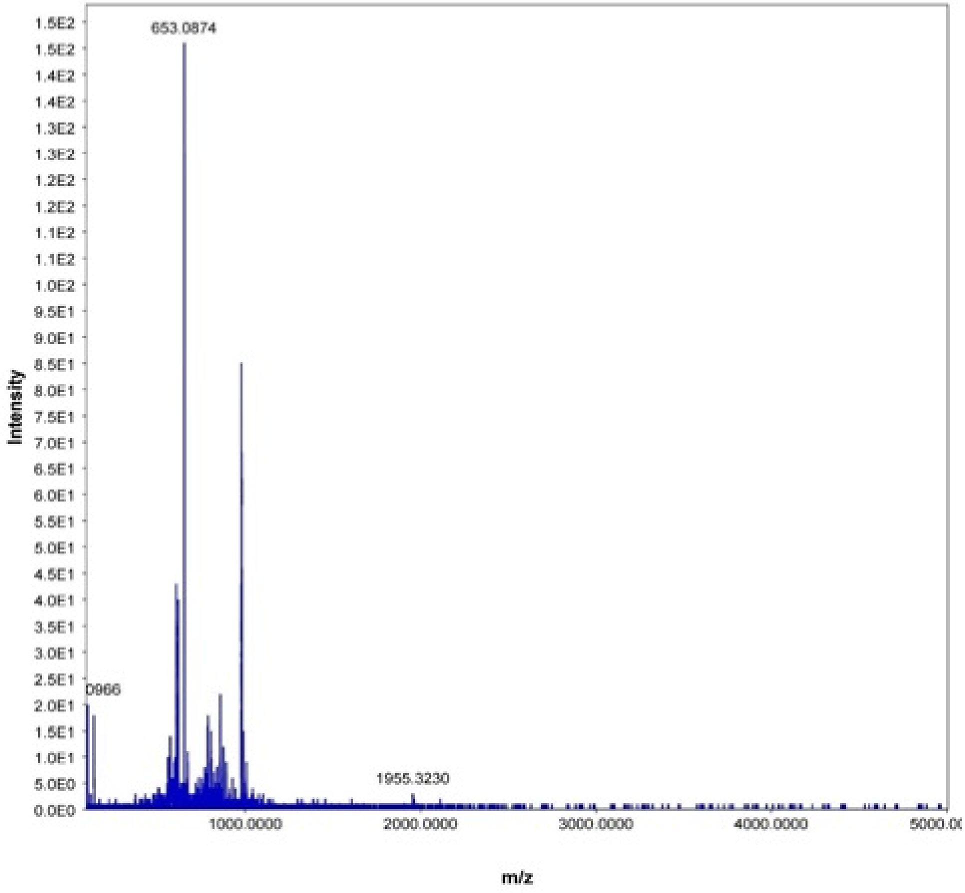

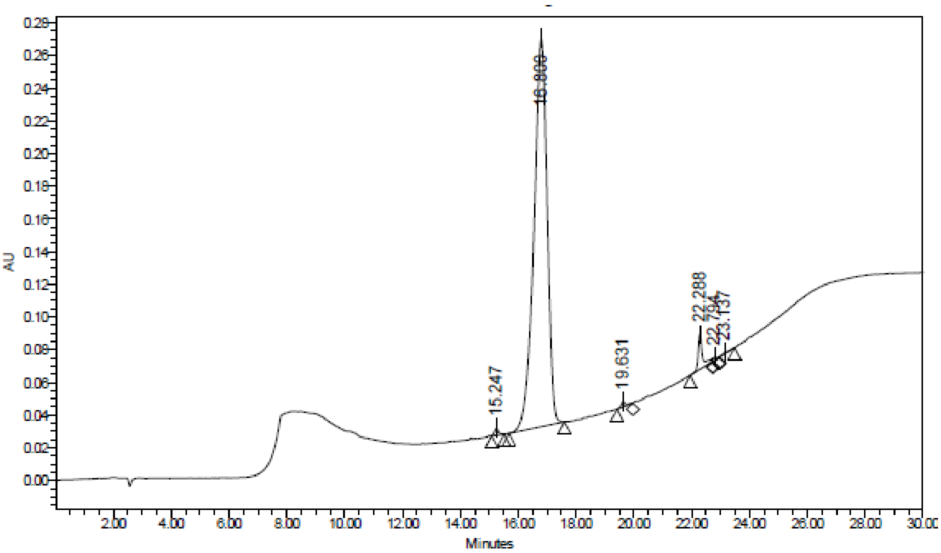

Peak Results

	Name	RT	Area	Height	% Area
1		15.247	38733	4308	0.49
2		16.800	7518533	237500	96.01

Compound 39. Charge: +5. t_R = 16.926 min. Gradient: 0-100% B over 20 min. B = 90% MeCN + 0.1% TFA. HRMS: Exact Mass: 1953.9230, Base peak ion: m/z 653.0874 $(M+3H)^{3+}$

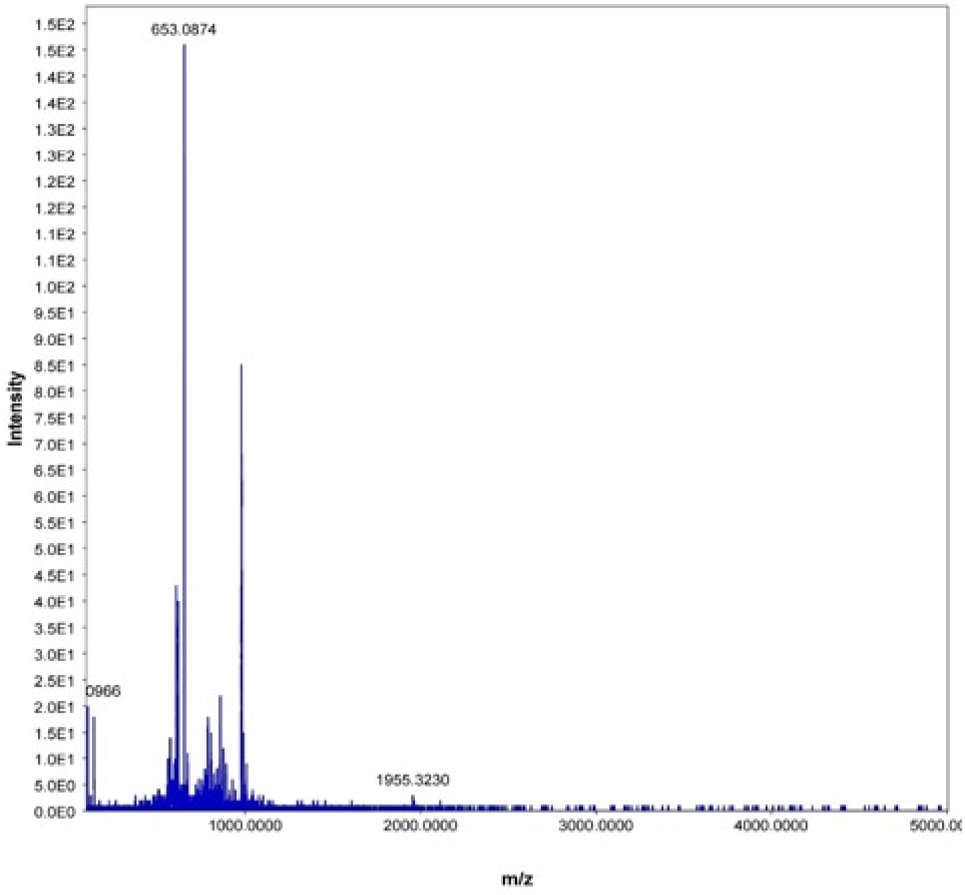

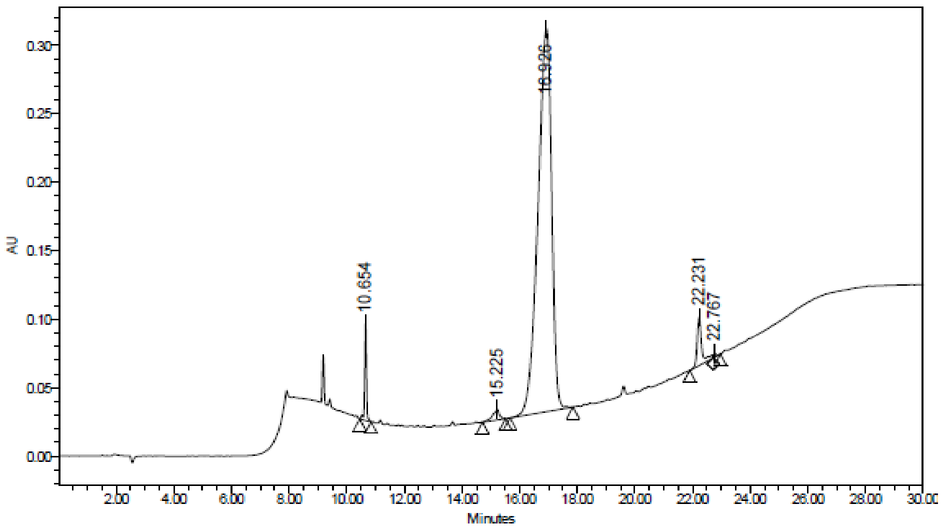

Peak Results

	Name	RT	Area	Height	% Area
1		10.654	329428	70802	3.27
2		15.225	124291	7742	1.23
3		16.926	9214410	279605	91.41

Compound 40. Charge: +5. t_R = 16.085 min. Gradient: 0-100% B over 20 min. B = 90% MeCN + 0.1% TFA. HRMS: Exact Mass: 1886.0010, Base peak ion: m/z 945.164 $(M+2H)^{2+}$

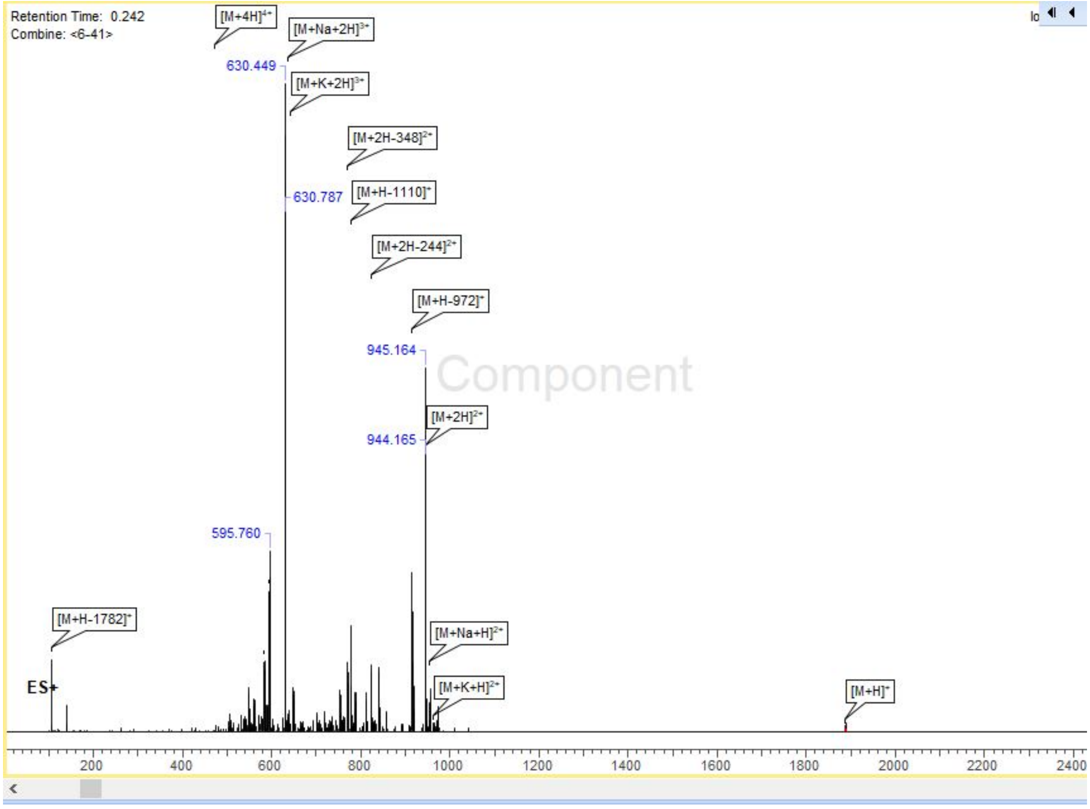

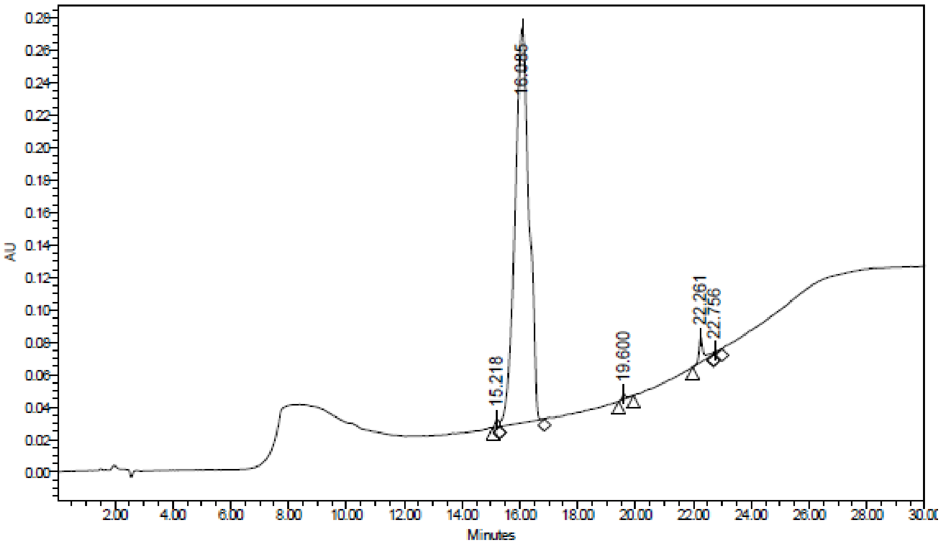

Peak Results

	Name	RT	Area	Height	% Area
1		15.218	38638	4612	0.46
2		16.085	8228734	243818	97.11

Compound 41. Charge: +5. t_R = 16.201 min. Gradient: 0-100% B over 20 min. B = 90% MeCN + 0.1% TFA. HRMS: Exact Mass: 1886.0010, Base peak ion: m/z 945.164 $(M+2H)^{2+}$

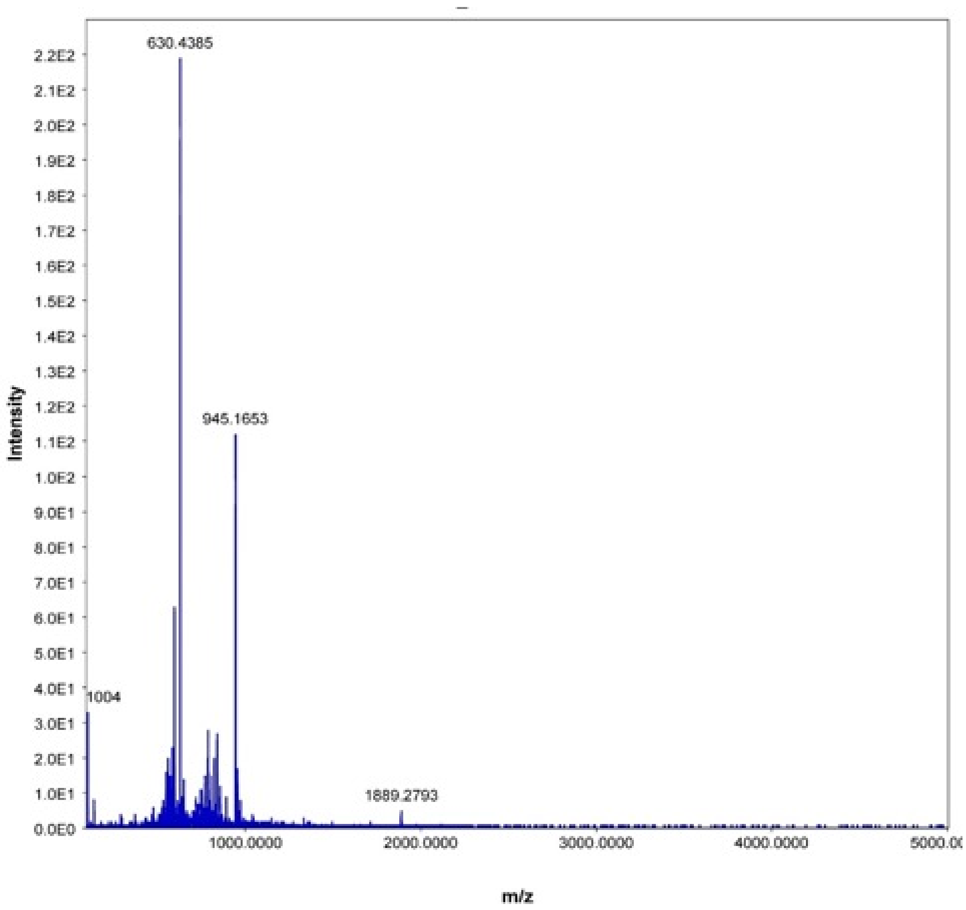

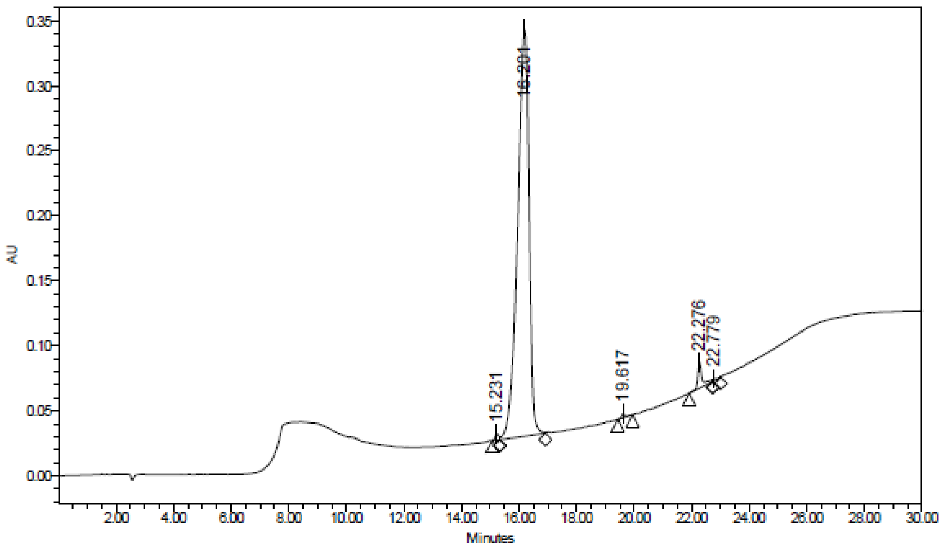

Peak Results

	Name	RT	Area	Height	% Area
1		15.231	38454	4461	0.43
2		16.201	8625379	312678	96.79

Compound 42. Charge: +5. t_R = 16.863 min. Gradient: 0-100% B over 20 min. B = 90% MeCN + 0.1% TFA. HRMS: Exact Mass: 1953.9230, Base peak ion: m/z 979.216 $(M+2H)^{2+}$

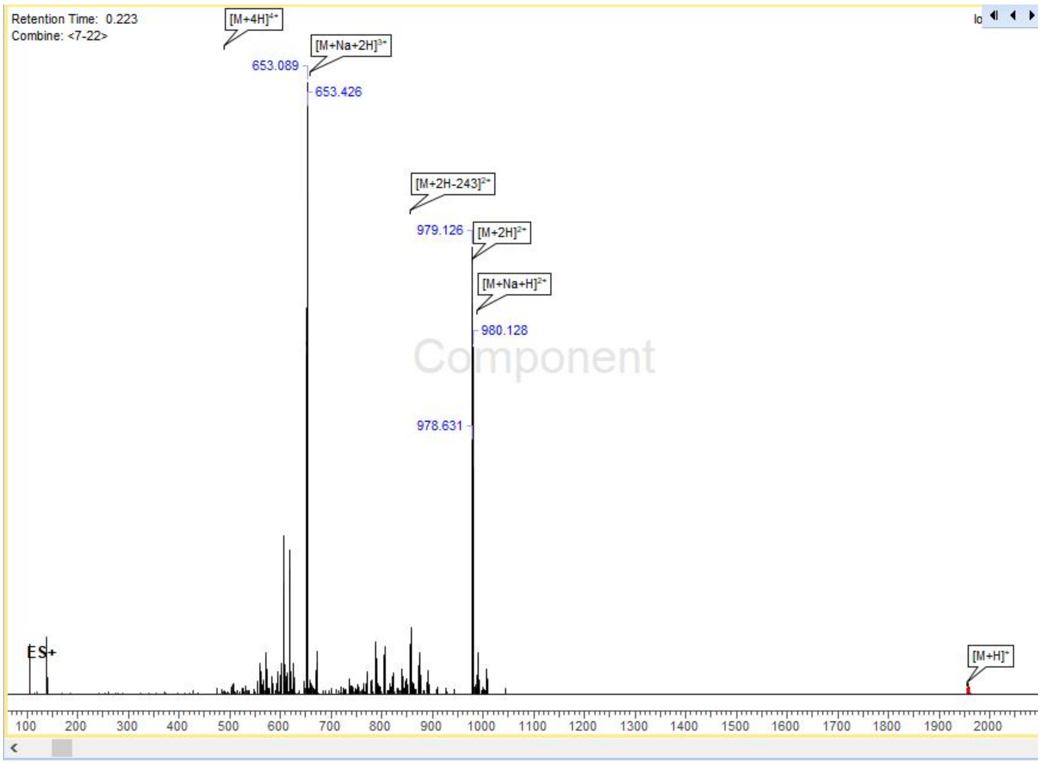

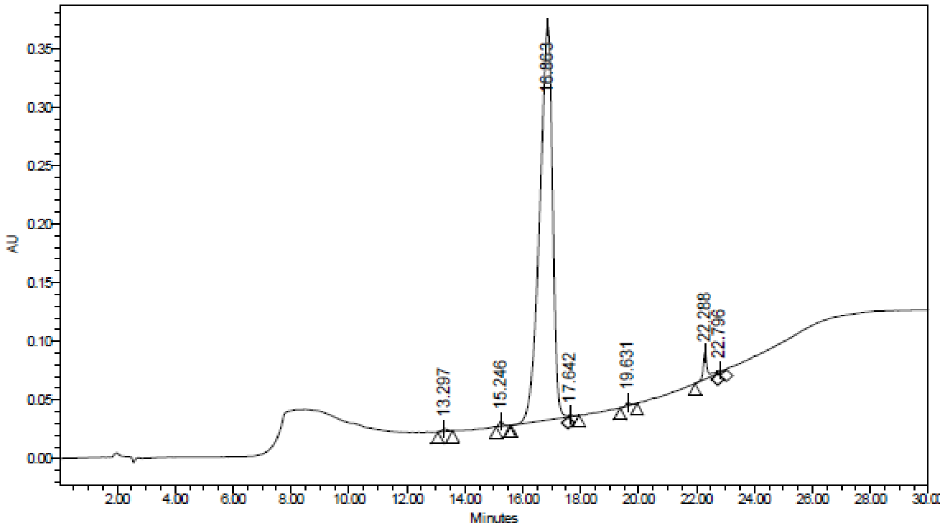

Peak Results

	Name	RT	Area	Height	% Area
1		13.297	30561	2263	0.28
2		15.246	39634	4410	0.37
3		16.863	10413808	335428	96.67

Compound 43. Charge: +5. t_R = 18.882 min. Gradient: 0-100% B over 20 min. B = 90% MeCN + 0.1% TFA. HRMS: Exact Mass: 2450.3630, Base peak ion: m/z 817.5964 $(M+3H)^{3+}$

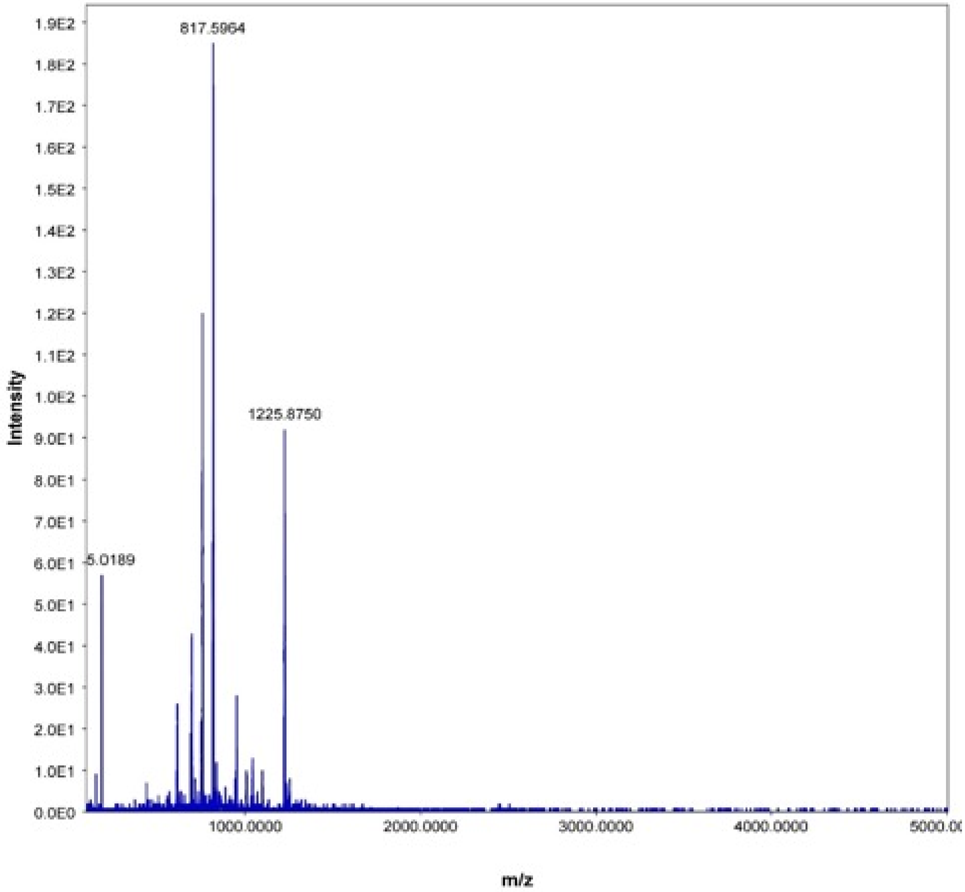

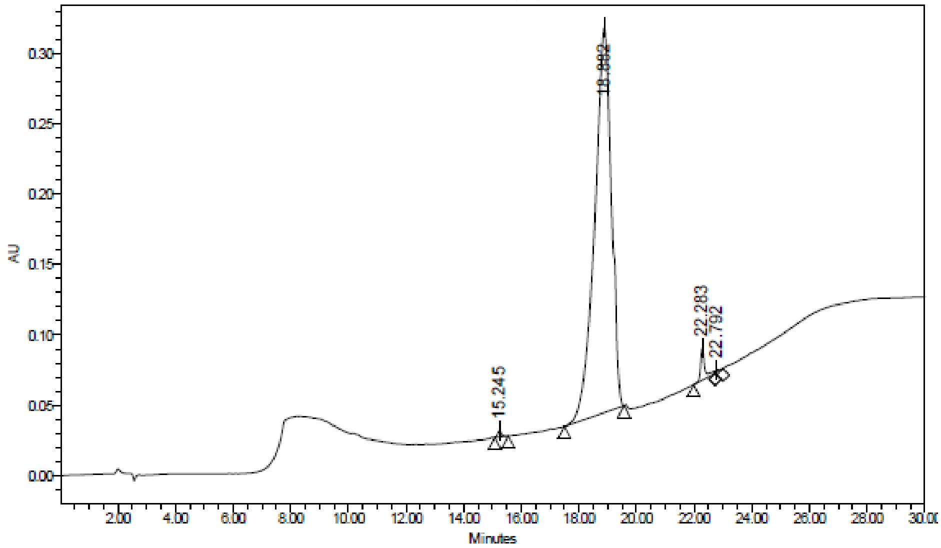

Peak Results

	Name	RT	Area	Height	% Area
1		15.245	39751	4454	0.35
2		18.882	10982208	273440	97.53

Compound 44. Charge: +5. t_R = 17.035 min. Gradient: 0-100% B over 20 min. B = 90% MeCN + 0.1% TFA. HRMS: Exact mass 2129.7210, Base peak ion: m/z 712.367 $(M+3H)^{3+}$

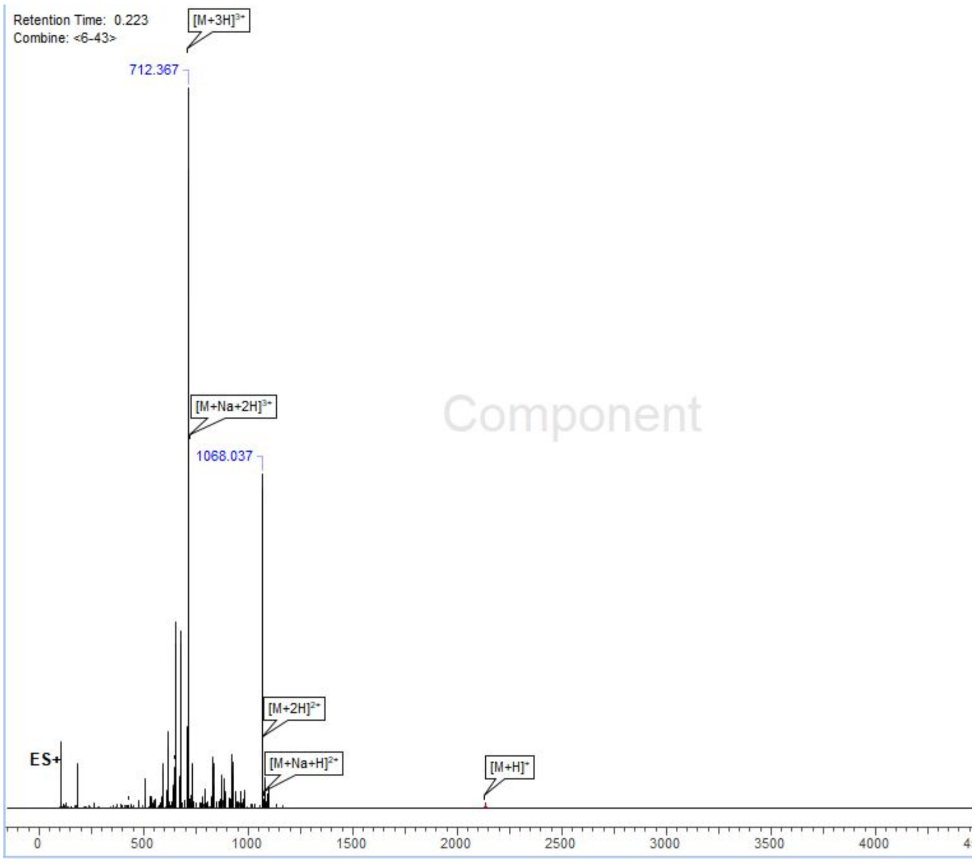

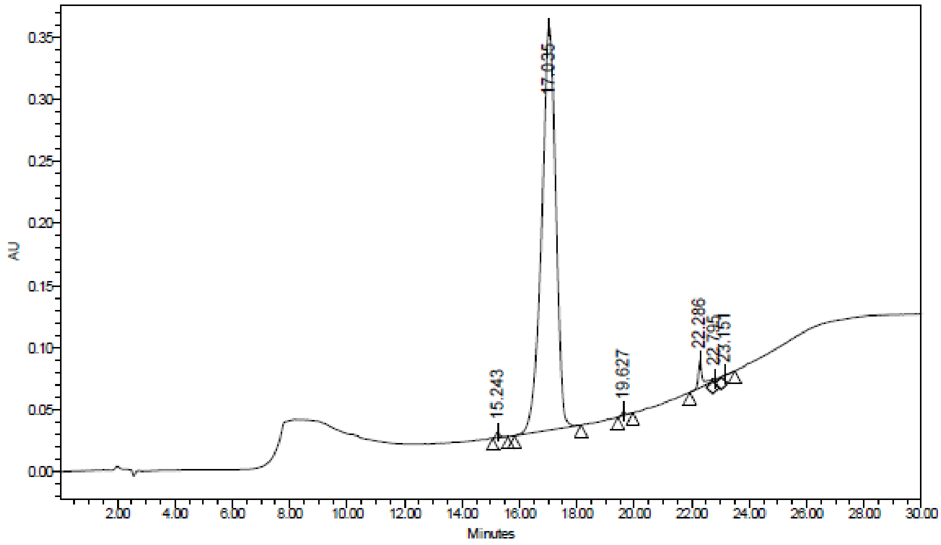

Peak Results

	Name	RT	Area	Height	% Area
1		15.243	41225	4499	0.36
2		17.035	11092031	324370	97.21

Compound 45. Charge: +5. t_R = 17.214 min. Gradient: 0-100% B over 20 min. B = 90% MeCN + 0.1% TFA. HRMS: Exact Mass: 2129.7210, Base peak ion: m/z 712.355 $(M+3H)^{3+}$

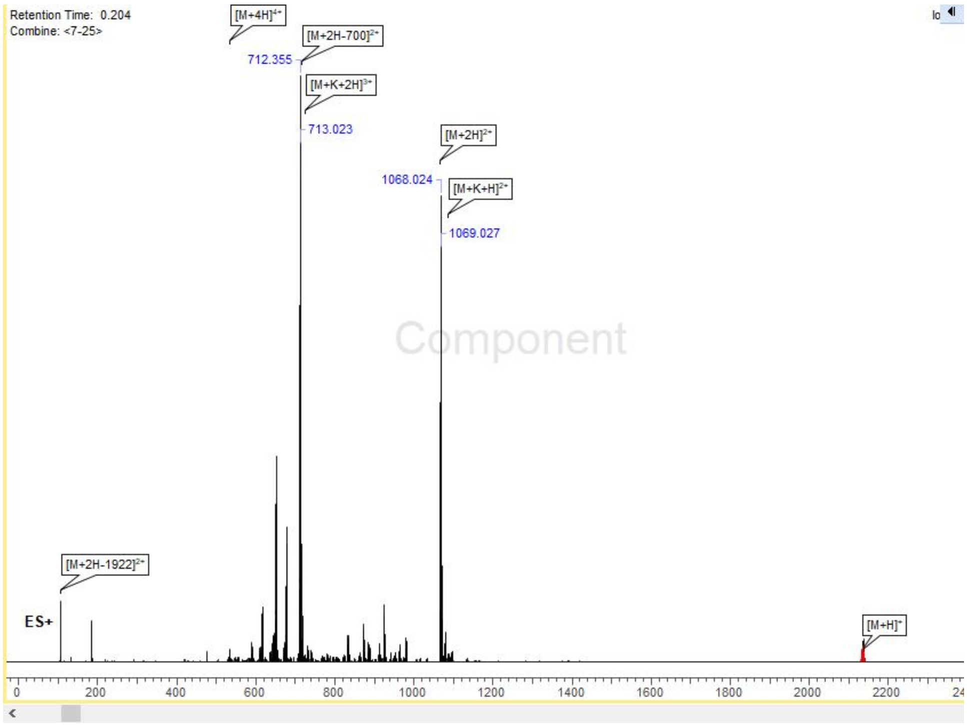

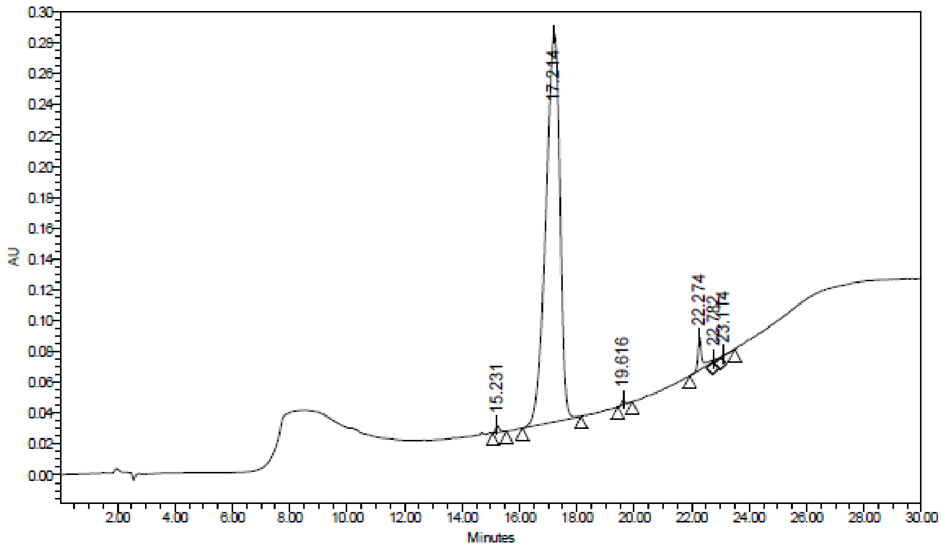

Peak Results

	Name	RT	Area	Height	% Area
1		15.231	40704	4409	0.46
2		17.214	8557592	251380	96.52

Compound 46. Charge: +5. t_R = 16.233 min. Gradient: 0-100% B over 20 min. B = 90% MeCN + 0.1% TFA. HRMS: Exact Mass: 1973.8999, Base peak ion: m/z 659.753 $(M+3H)^{3+}$

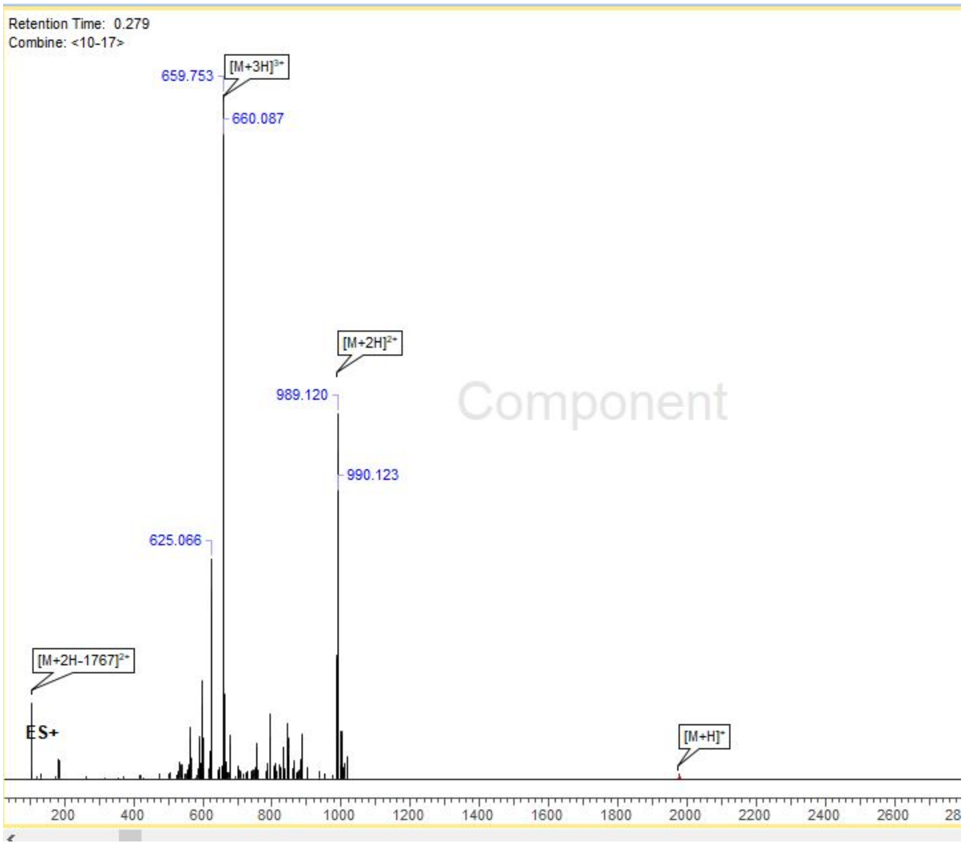

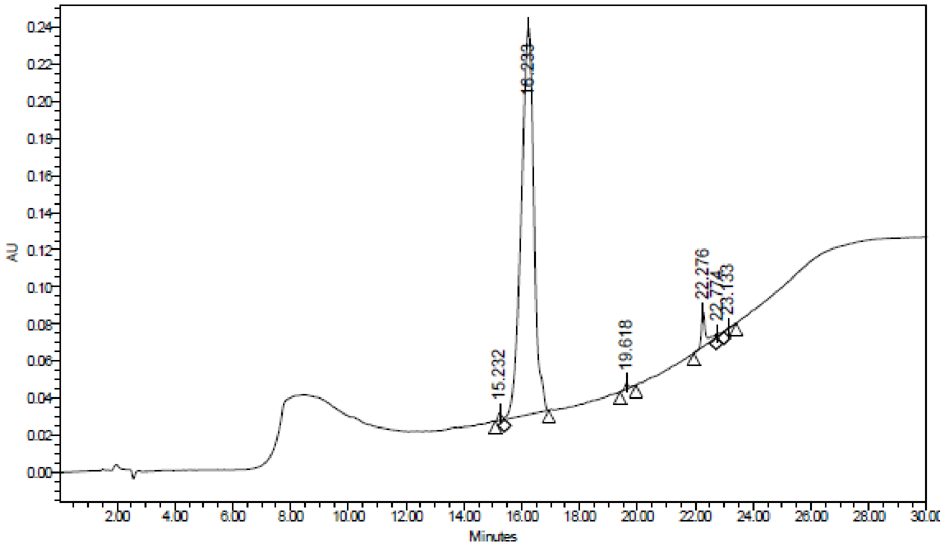

Peak Results

	Name	RT	Area	Height	% Area
1		15.232	38163	4049	0.58
2		16.233	6334455	208304	95.74

Compound 47. Charge: +5. t_R = 15.676 min. Gradient: 0-100% B over 20 min. B = 90% MeCN + 0.1% TFA.

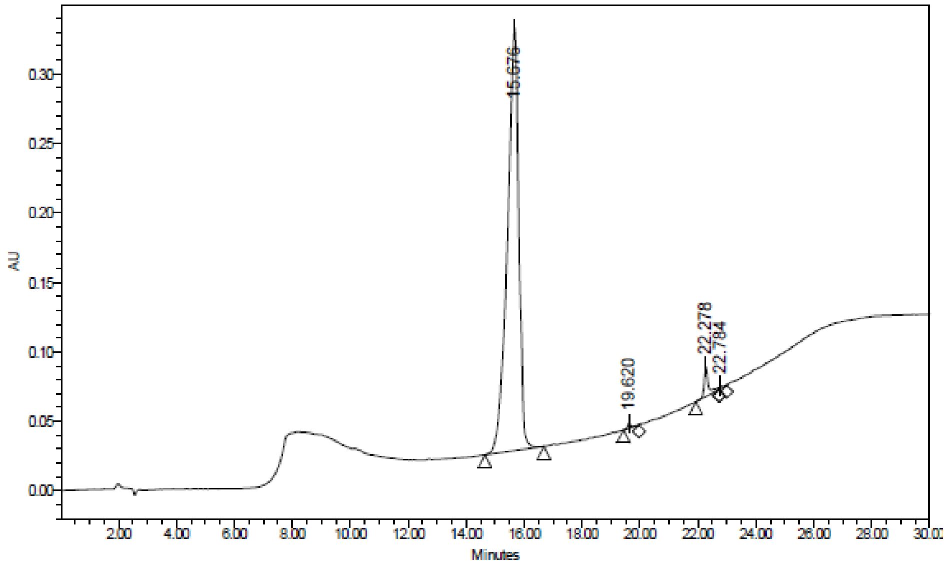

Peak Results

	Name	RT	Area	Height	% Area
1		15.676	8310514	304413	96.96

Compound 48. Charge: +5. t_R = 17.142 min. Gradient: 0-100% B over 20 min. B = 90% MeCN + 0.1% TFA. HRMS: Exact Mass: 2129.7210, Base peak ion: m/z 712.367 $(M+3H)^{3+}$

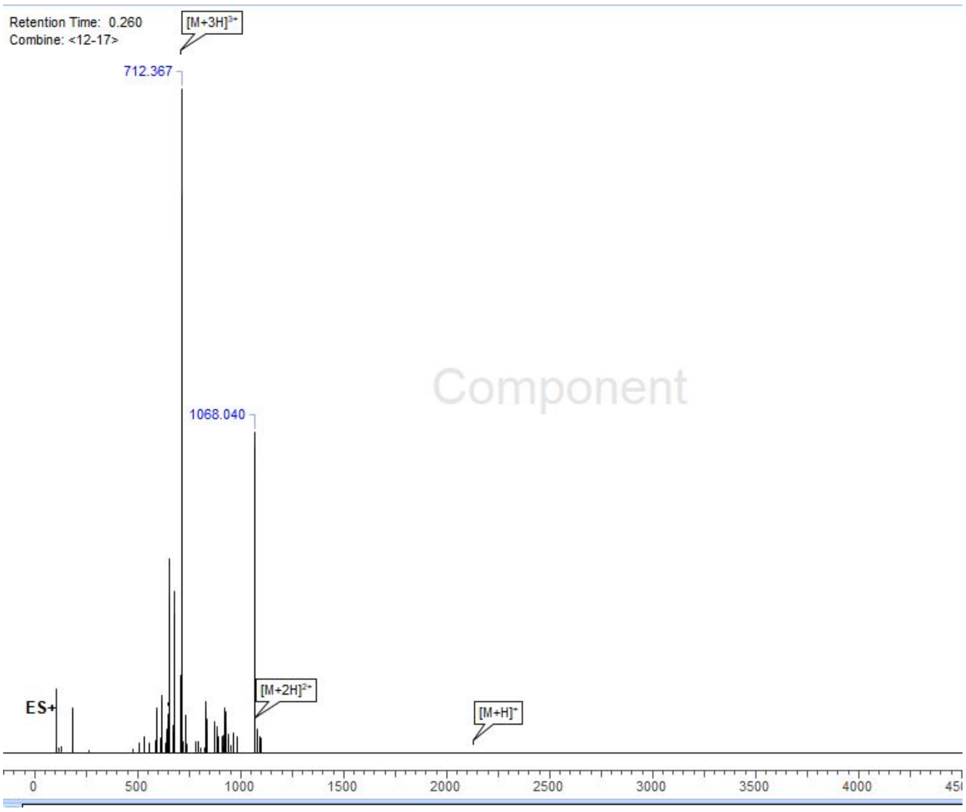

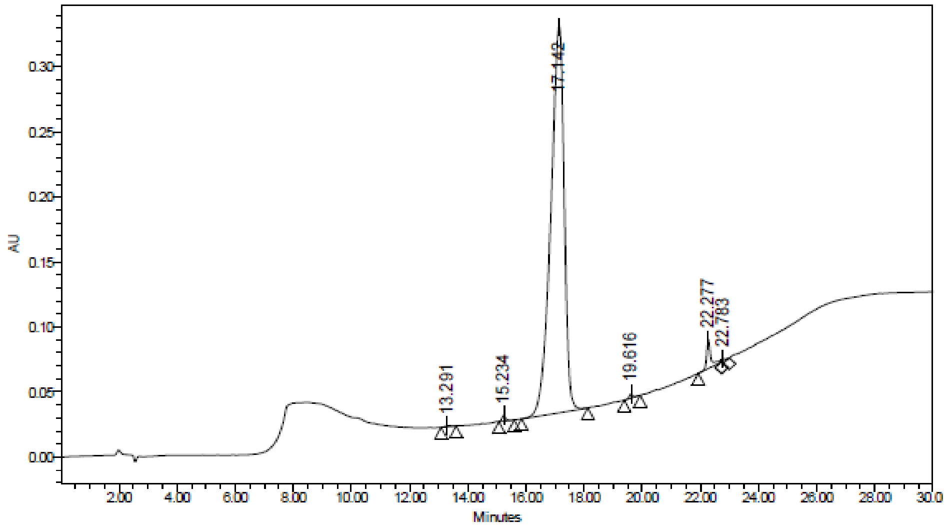

Peak Results

	Name	RT	Area	Height	% Area
1		13.291	11004	853	0.11
2		15.234	38887	4410	0.40
3		17.142	9561078	297473	96.84

Peptoid 1

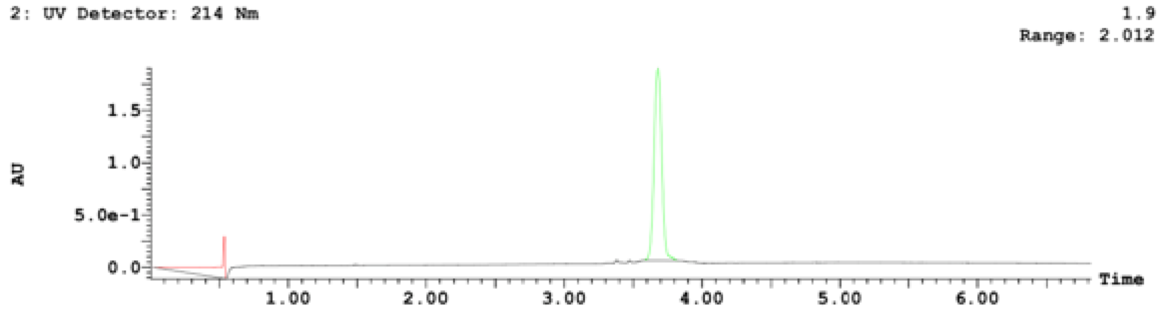

Peak ID	Time	Mass Found
2	3.91	2.62,455.91,607.52,910.64,1820.70

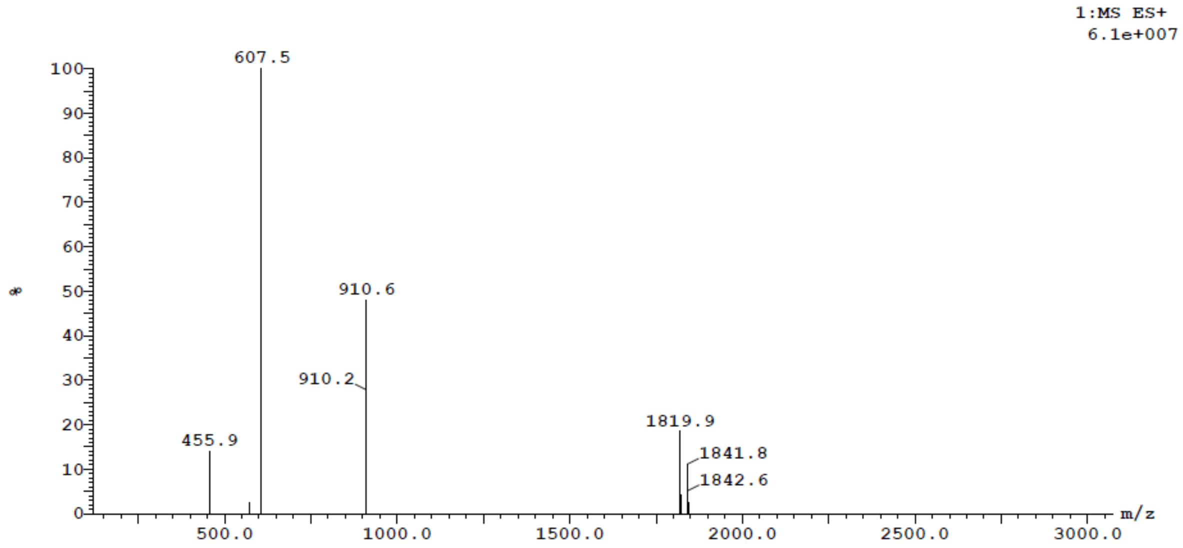

Pep1-6mer

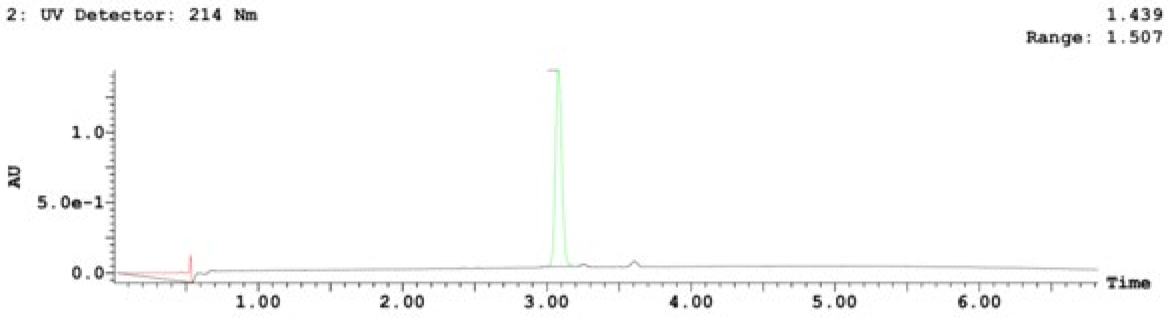

Peak ID	Time	Mass Found
4	3.09	459.68,940.60,459.68,918.61

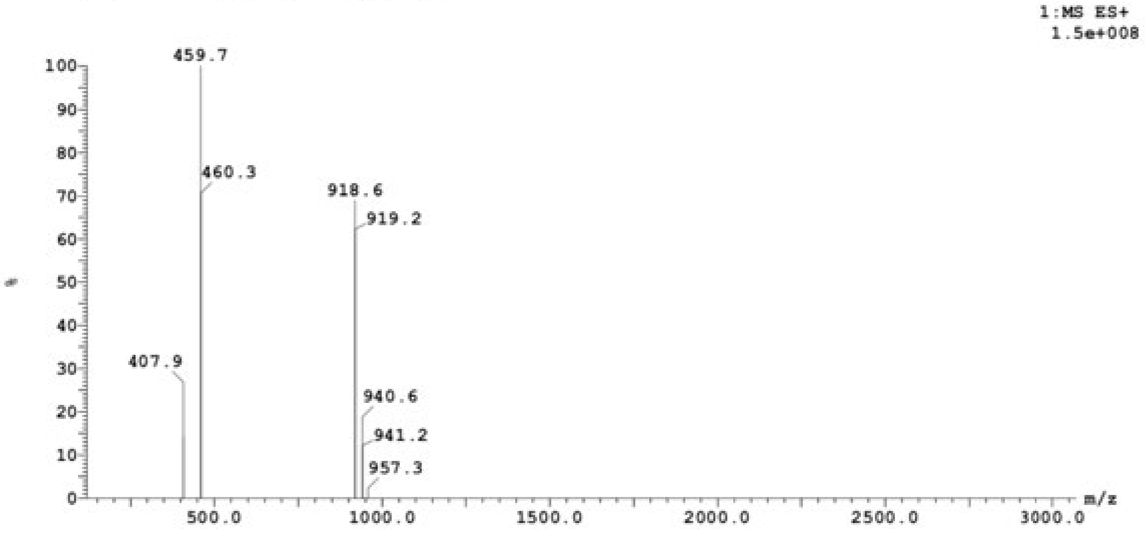

Compound 49

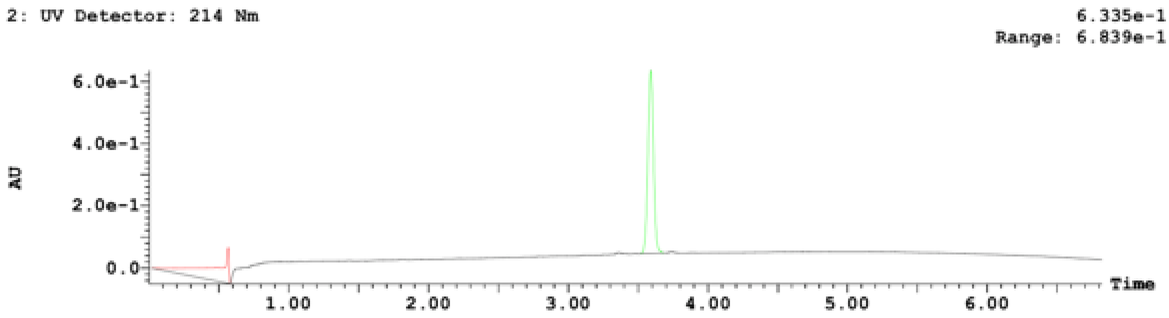

Peak ID	Time	Mass Found
2	3.60	[illegible].50,498.95,1020.46,499.83,998.46

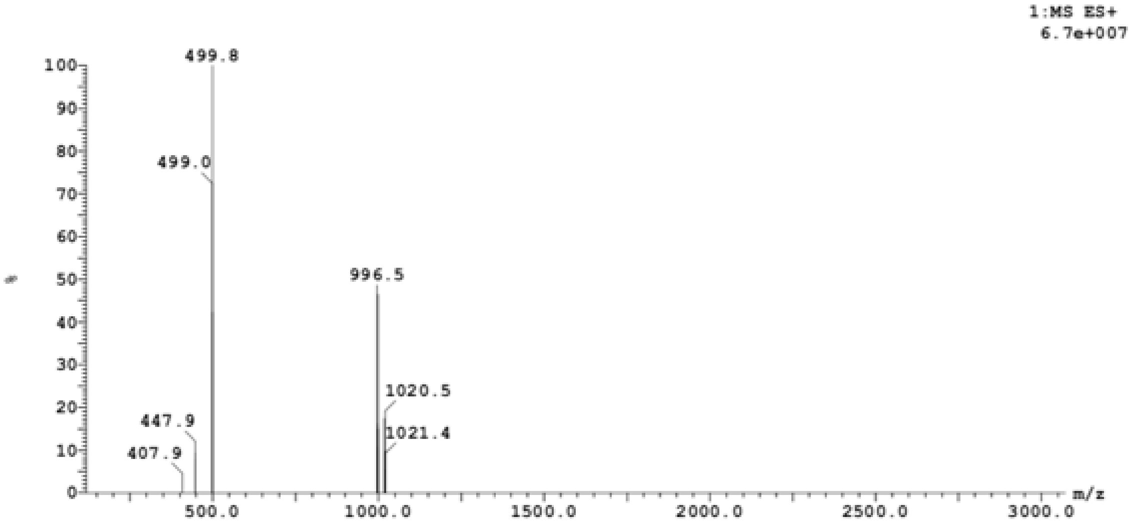

Compound 50

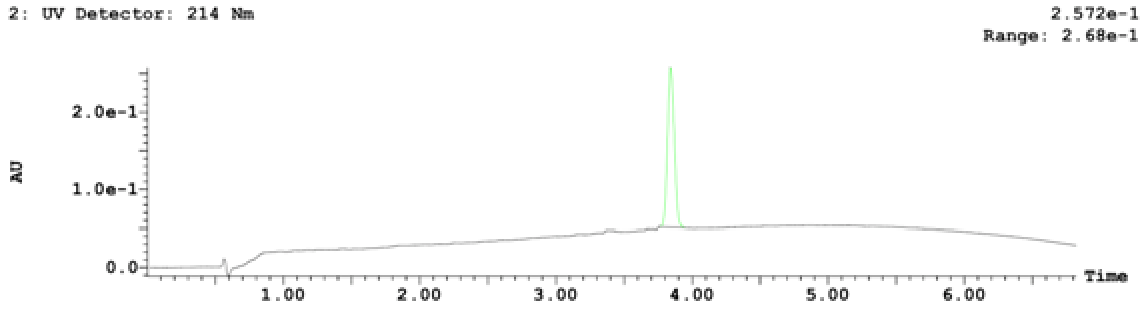

Peak ID	Time	Mass Found
6	3.58	33,537.84,1098.32,538.80,1076.32

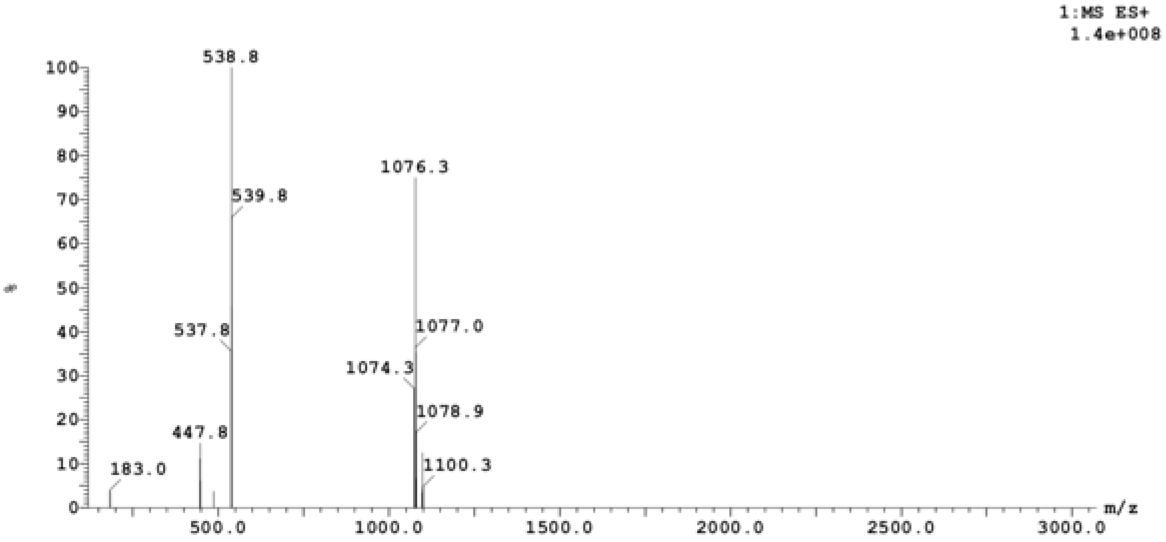

Compound 51

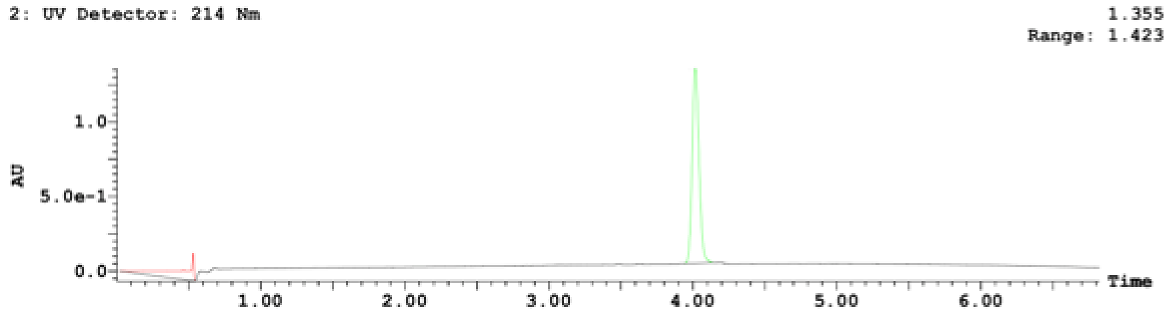

Peak ID	Time	Mass Found
2	4.02	4,1232.05,1256.04,617.70,1234.05

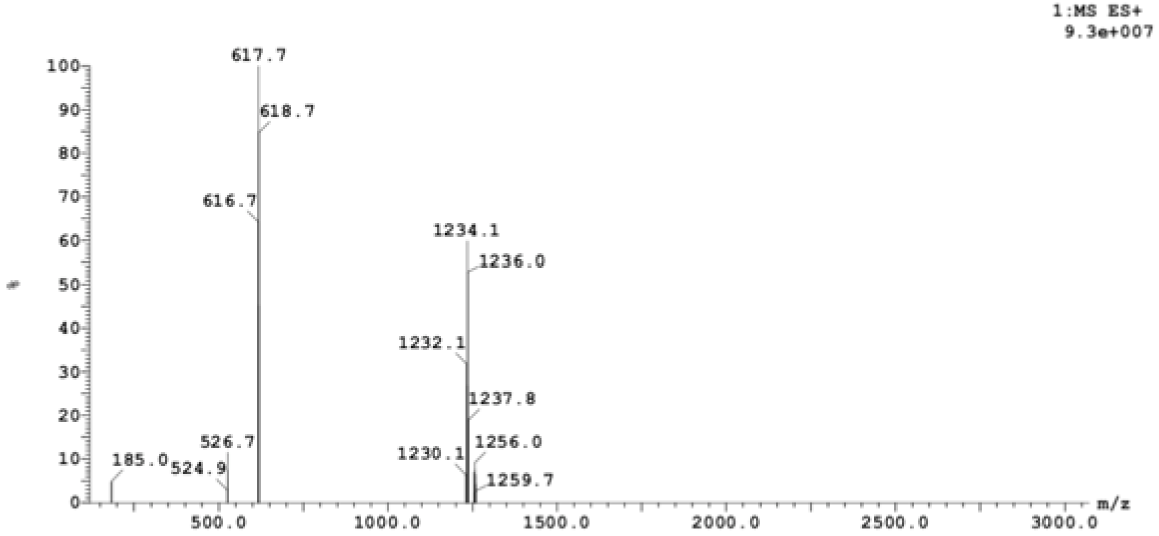

Supplement: Supplementary file 1 — Supplementary information [file 41598_2020_71771_MOESM1_ESM.pdf]
